# Supplementary material for: [1,5]-Hydride Shift Triggered N-Dealkylative Cyclization into 2-Oxo-1,2,3,4-tetrahydroquinoline-3-carboxylates via Boronate Complexes
Source: Molecules. 2022 Aug 18;27(16):5270. doi: 10.3390/molecules27165270 (PMC9414529; doi:10.3390/molecules27165270)
Supplement: Supplementary file 1 [file molecules-27-05270-s001.zip › molecules-1845883-supplementary-new.pdf]

Contents

1. General **S2**

2. Synthesis of starting aldehydes **S3**

3. Synthesis of benzylmalonates 1a-p **S6**

4. NMR study on the reaction intermediates **S12**

5. References **S16**

6. Copies of ^{1}H and ^{13}C NMR spectra **S17**

1. General

Commercially available reagents were used without additional purification. E. Merck Kieselgel 60 was used for column chromatography.

Thin-layer chromatography (TLC) was performed on silica gel 60 F254 glass-backed plates (MERCK). Visualization was performed using UV light (254 or 312 nm) or staining with $KMnO_4$.

NMR spectra (except NMR study from Part 4) were recorded on a 700 MHz Bruker Avance III NMR at 303K, Bruker Avance III 800 (with a 5-mm CPTXI cryoprobe) and Bruker Fourier 300. Chemical shifts were reported relative to residue peaks of DMSO-d_6 (2.51 ppm for ^{1}H and 39.5 ppm for ^{13}C).

Melting points were measured on a SMP 30 apparatus without correction.

High-resolution mass spectra (HRMS) spectra were recorded on AB Sciex TripleTOF® 5600+ System using electrospray ionization (ESI). The measurements were done in a positive ion mode (interface capillary voltage – 5500 V); mass range from m/z 50 to *m/z* 3000; external or internal calibration was done with ESI Tuning Mix, Agilent. A syringe injection was used for solutions in acetonitrile, methanol, or water (flow rate 20 μl/min). Nitrogen was applied as a dry gas; interface temperature was set at 180 °C. IUPAC compound names were generated using ChemDraw Software.

2. Synthesis of starting aldehydes

General method

Mixture of the corresponding 2-fluorobenzaldehyde (10 mmol), benzylamine (13 mmol) and K_2CO_3 (2.07 g, 15 mmol) in of freshly distilled DMF (50 mL) was heated at 100°C for 12 h. EtOAc (200 mL) was added and the resulted mixture was washed with brine (3×30 mL). Organic layer was dried over anhydrous Na_2SO_4, all volatiles were removed in vacuo and the residue was purified by flash chromatography (eluent – mixture of hexane and EtOAc, v/v 10:1).

2-(Benzyl(methyl)amino)benzaldehyde, 2-(benzyl(methyl)amino)-5-methylbenzaldehyde,[1] 2-(benzyl(ethyl)amino)benzaldehyde,[2] 2-(benzyl(methyl)amino)-5-methoxybenzaldehyde,[3] 2-(diethylamino)benzaldehyde[4] and 2-(benzyl(methyl)amino)-5-nitrobenzaldehyde[5] were synthesized by the same method, the spectral properties corresponded to the literature data.

2-(Benzyl(methyl)amino)-3-chlorobenzaldehyde

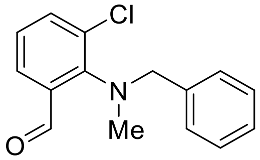

Yield 1.35 g (52%), yellow viscous oil.

^{1}H NMR (700 MHz, DMSO-*d*$_6$) δ ppm: 2.88 (s, 3H), 4.35 (s, 2H), 7.21 - 7.25 (m, 1H), 7.27 - 7.33 (m, 5H), 7.62 (dd, *J*=7.7, 1.6 Hz, 1H), 7.75 (dd, *J*=7.8, 1.7 Hz, 1H), 10.27 (d, *J*=0.6 Hz, 1H).

^{13}C NMR (75 MHz, DMSO-*d*$_6$) δ ppm: 41.3, 59.3, 126.7, 127.2, 127.3, 128.3, 128.6, 133.9, 135.8, 136.3, 138.3, 150.6, 191.6.

HRMS (ESI-TOF) found, m/z: 260.0836 $[M+H]^+$. $C_{15}H_{15}ClNO^+$. Calculated, m/z: 260.0837.

2-(Benzyl(methyl)amino)-5-chlorobenzaldehyde

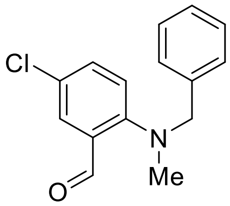

Yield 2.43 g (94%), yellow viscous oil.

^{1}H NMR (700 MHz, DMSO-*d*$_6$) δ ppm: 2.81 (s, 3H), 4.38 (s, 2H), 7.20 (d, *J*=9.0 Hz, 1H), 7.25 - 7.28 (m, 3H), 7.33 (t, *J*=7.5 Hz, 2H), 7.54 (dd, *J*=8.9, 2.8 Hz, 1H), 7.64 (d, *J*=2.7 Hz, 1H), 10.17 (s, 1H).

^{13}C NMR (75 MHz, DMSO-*d*$_6$) δ ppm: 42.5, 60.7, 121.7, 125.0, 127.3, 127.7, 128.0, 128.5, 129.0, 134.1, 137.3, 153.3, 189.5.

HRMS (ESI-TOF) found, m/z: 260.0838 $[M+H]^+$. $C_{15}H_{15}ClNO^+$. Calculated, m/z: 260.0837.

2-(Benzyl(methyl)amino)-4-bromobenzaldehyde

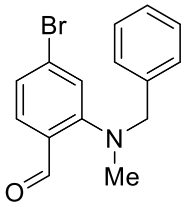

Yield 2.45 g (81%), yellow viscous oil.

^{1}H NMR (700 MHz, DMSO-d_6) δ ppm: 2.81 (s, 3H), 4.41 (s, 2H), 7.22 (dd, *J*=8.3, 1.1 Hz, 1H), 7.25 - 7.29 (m, 3H), 7.32 (d, *J*=1.3 Hz, 1H), 7.35 (t, *J*=7.6 Hz, 2H), 7.61 (d, *J*=8.2 Hz, 1H), 10.13 (s, 1H).

^{13}C NMR (176 MHz, DMSO-d_6) δ ppm: 42.2, 60.4, 122.0, 123.5, 125.7, 127.2, 127.6, 128.4, 128.6, 132.1, 137.2, 155.2, 189.7.

HRMS (ESI-TOF) found, m/z: 304.0337 $[M+H]^+$. $C_{15}H_{15}BrNO^+$. Calculated, m/z: 304.0332.

2-(Benzyl(methyl)amino)-4-(trifluoromethyl)benzaldehyde

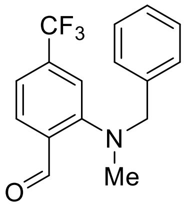

Yield 2.20 g (75%), yellow viscous oil.

^{1}H NMR (700 MHz, DMSO-d_6) δ ppm: 2.81 (s, 3H), 4.41 (s, 2H), 7.22 (dd, *J*=8.3, 1.1 Hz, 1H), 7.25 - 7.29 (m, 3H), 7.32 (d, *J*=1.3 Hz, 1H), 7.35 (t, *J*=7.6 Hz, 2H), 7.61 (d, *J*=8.2 Hz, 1H), 10.13 (s, 1H).

^{13}C NMR (201 MHz, DMSO-d_6) δ ppm: 42.4, 60.2, 115.7 (q, *J*=4.4 Hz), 116.4 (q, *J*=4.4 Hz), 123.6 (q, *J*=272.9 Hz), 127.3, 127.6, 128.5, 129.1, 131.4, 133.7 (q, *J*=32.3 Hz), 137.2, 154.2, 190.1.

HRMS (ESI-TOF) found, m/z: 294.1102 $[M+H]^+$. $C_{16}H_{15}F_3NO^+$. Calculated, m/z: 294.1100.

2-(Benzyl(ethyl)amino)-5-bromobenzaldehyde

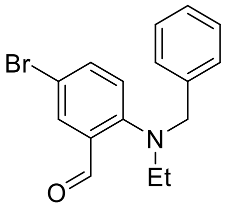

Yield 2.95 g (93%), yellow viscous oil.

^{1}H NMR (700 MHz, DMSO-d_6) δ ppm: 1.04 (t, *J*=7.1 Hz, 3H), 3.18 (q, *J*=7.1 Hz, 2H), 4.36 (s, 2H), 7.20 - 7.24 (m, 2H), 7.26 - 7.30 (m, 4H), 7.66 (dd, *J*=8.8, 2.5 Hz, 1H), 7.72 (d, *J*=2.5 Hz, 1H), 10.21 (s, 1H).

^{13}C NMR (75 MHz, DMSO-d_6) δ ppm: 11.8, 50.3, 56.3, 114.1, 124.5, 127.1, 128.1, 128.4, 130.7, 131.0, 136.7, 137.8, 152.7, 189.7.

HRMS (ESI-TOF) found, m/z: 318.0486 $[M+H]^+$. $C_{16}H_{17}BrNO^+$. Calculated, m/z: 318.0488.

2-(Benzyl(ethyl)amino)-4-methoxybenzaldehyde

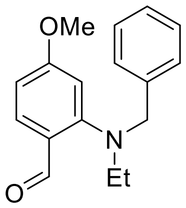

Yield 2.37 g (88%), yellow viscous oil.

^{1}H NMR (700 MHz, DMSO-d_6) δ ppm: 1.04 (t, *J*=7.1 Hz, 3H), 3.18 (q, *J*=7.1 Hz, 2H), 3.79 (s, 3H), 4.35 (s, 2H), 6.67 (dd, *J*=8.6, 2.1 Hz, 1H), 6.70 (d, *J*=2.3 Hz, 1H), 7.21 (t, *J*=7.1 Hz, 1H), 7.27 - 7.32 (m, 4H), 7.65 (d, *J*=8.6 Hz, 1H), 10.15 (s, 1H).

^{13}C NMR (75 MHz, DMSO-d_6) δ ppm: 11.7, 50.4, 55.5, 56.0, 106.9, 108.4, 123.0, 127.0, 128.1, 128.3, 131.3, 138.3, 155.8, 164.3, 189.1.

HRMS (ESI-TOF) found, m/z: 270.1491 $[M+H]^+$. $C_{17}H_{20}NO_2^+$. Calculated, m/z: 270.1489.

3. Synthesis of benzylmalonates 1a-p

General method

5 Mmol of corresponding aldehyde and 5 mmol of dimethyl or diethyl malonate were dissolved in 40 mL of toluene, 20 mg of benzoic acid and 20 mg of piperidine were added and the resulted mixture was refluxed in argon for 24 h. All volatiles were removed in vacuo and the residue was purified by flash chromatography (eluent – mixture of hexane and EtOAc, v/v 8:1).

Dimethyl 2-(2-(benzyl(methyl)amino)benzylidene)malonate (**1a**)[2] and dimethyl 2-(2-(benzyl(ethyl)amino)benzylidene)malonate (**1o**)[6] were synthesized by the same method, the spectral properties corresponded to the literature data.

Diethyl 2-(2-(benzyl(methyl)amino)benzylidene)malonate (1b)

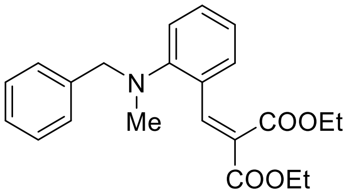

Yield 1.27 g (69%), yellow viscous oil.

^{1}H NMR (700 MHz, DMSO-d_6) δ ppm: 1.13 (t, *J*=7.1 Hz, 3H), 1.23 (t, *J*=7.1 Hz, 3H), 2.62 (s, 3H), 4.08 (s, 2H), 4.20 (qd, *J*=7.1, 5.3 Hz, 4H), 7.05 (t, *J*=7.4 Hz, 1H), 7.12 (d, *J*=7.8 Hz, 1H), 7.22 - 7.29 (m, 4H), 7.32 (t, *J*=7.4 Hz, 2H), 7.36 - 7.40 (m, 1H), 8.04 (s, 1H).

^{13}C NMR (75 MHz, DMSO-d_6) δ ppm: 13.7, 14.0, 40.4, 61.0, 61.2, 61.3, 119.7, 122.3, 125.4, 126.7, 127.3, 128.2, 128.3, 128.6, 131.3, 137.5, 141.0, 152.4, 163.6, 165.7.

HRMS (ESI-TOF) found, m/z: 368.1865 $[M+H]^+$. $C_{22}H_{26}NO_4^+$. Calculated, m/z: 368.1856.

Dimethyl 2-(2-(benzyl(methyl)amino)-5-methylbenzylidene)malonate (1c)

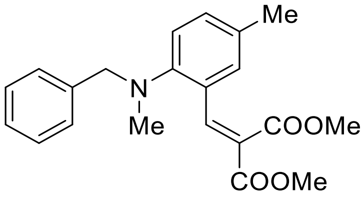

Yield 1.32 g (75%), yellow viscous oil.

^{1}H NMR (700 MHz, DMSO-d_6) δ ppm: 2.24 (s, 3H), 2.59 (s, 3H), 3.72 (s, 3H), 3.75 (s, 3H), 4.03 (s, 2H), 7.02 (s, 1H), 7.04 (d, *J*=8.2 Hz, 1H), 7.21 (dd, *J*=8.3, 1.4 Hz, 1H), 7.23 - 7.28 (m, 3H), 7.32 (t, *J*=7.4 Hz, 2H), 8.08 (s, 1H).

^{13}C NMR (75 MHz, DMSO-d_6) δ ppm: 20.3, 41.0, 52.5, 52.6, 61.1, 120.0, 124.5, 126.6, 127.2, 128.3, 128.5, 131.5, 132.1, 137.6, 141.2, 150.3, 164.1, 166.3.

HRMS (ESI-TOF) found, m/z: 354.1701 $[M+H]^+$. $C_{21}H_{24}NO_4^+$. Calculated, m/z: 354.1700.

Dimethyl 2-(2-(benzyl(methyl)amino)-3-chlorobenzylidene)malonate (1d)

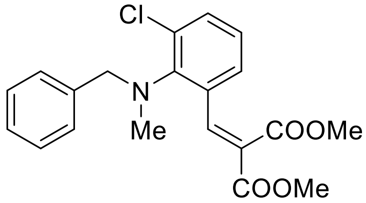

Yield 1.31 g (70%), yellow solid, m.p. 61–63 °C.

^{1}H NMR (700 MHz, DMSO-d_6) δ ppm: 2.72 (s, 3H), 3.66 (s, 3H), 3.80 (s, 3H), 4.22 (s, 2H), 7.17 (dd, *J*=7.8, 0.8 Hz, 1H), 7.23 (t, *J*=7.8 Hz, 1H), 7.25 - 7.28 (m, 1H), 7.30 - 7.34 (m, 4H), 7.53 (dd, *J*=8.0, 1.1 Hz, 1H), 8.04 (s, 1H).

^{13}C NMR (75 MHz, DMSO-d_6) δ ppm: 40.0, 52.5, 52.8, 59.0, 126.6, 126.8, 127.2, 127.3, 128.3, 128.5, 132.8, 133.2, 134.6, 138.4, 141.8, 147.6, 163.7, 165.7.

HRMS (ESI-TOF) found, m/z: 374.1158 $[M+H]^+$. $C_{20}H_{21}ClNO_4^+$. Calculated, m/z: 374.1154.

Dimethyl 2-(2-(benzyl(methyl)amino)-4-chlorobenzylidene)malonate (1e)

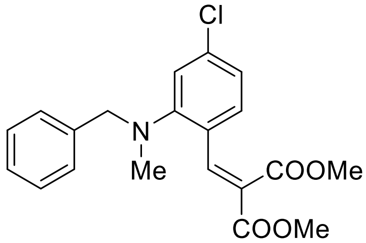

Yield 1.73 g (93%), yellow solid, m.p. 62–64 °C.

^{1}H NMR (700 MHz, DMSO-d_6) δ ppm: 2.65 (s, 3H), 3.72 (s, 3H), 3.73 (s, 3H), 4.13 (s, 2H), 7.09 - 7.13 (m, 2H), 7.20 (d, *J*=8.0 Hz, 1H), 7.24 - 7.29 (m, 3H), 7.32 - 7.36 (m, 2H), 7.92 (s, 1H).

^{13}C NMR (75 MHz, DMSO-d_6) δ ppm: 40.4, 52.6, 52.6, 60.5, 119.7, 122.1, 125.0, 125.0, 127.4, 128.1, 128.4, 130.0, 135.8, 137.1, 140.6, 153.6, 163.9, 166.0

HRMS (ESI-TOF) found, m/z: 374.1158 $[M+H]^+$. $C_{20}H_{21}ClNO_4^+$. Calculated, m/z: 374.1154.

Dimethyl 2-(2-(benzyl(methyl)amino)-5-chlorobenzylidene)malonate (1f)

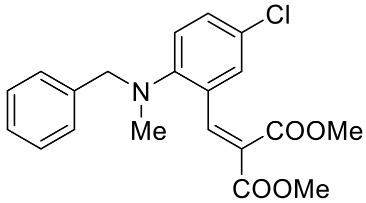

Yield 1.29 g (69%), yellow viscous oil.

^{1}H NMR (700 MHz, DMSO-d_6) δ ppm: 2.63 (s, 3H), 3.73 (s, 3H), 3.75 (s, 3H), 4.10 (s, 2H), 7.12 (d, *J*=8.8 Hz, 1H), 7.18 (d, *J*=2.5 Hz, 1H), 7.24 (d, *J*=7.1 Hz, 2H), 7.25 - 7.28 (m, 1H), 7.33 (t, *J*=7.4 Hz, 2H), 7.43 (dd, *J*=8.6, 2.5 Hz, 1H), 7.94 (s, 1H).

^{13}C NMR (75 MHz, DMSO-d_6) δ ppm: 40.6, 52.6, 52.7, 60.6, 121.7, 125.8, 125.9, 127.3, 127.6, 128.1, 128.2, 128.4, 130.8, 137.1, 140.1, 151.1, 163.7, 165.8.

HRMS (ESI-TOF) found, m/z: 374.1159 $[M+H]^+$. $C_{20}H_{21}ClNO_4^+$. Calculated, m/z: 374.1154.

Dimethyl 2-(2-(benzyl(methyl)amino)-6-chlorobenzylidene)malonate (1f)

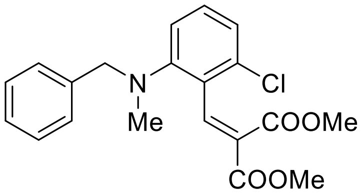

Yield 0.97 g (52%), yellow viscous oil.

^{1}H NMR (700 MHz, DMSO-*d*6) δ ppm: 2.58 (s, 3H), 3.61 (s, 3H), 3.74 (s, 3H), 4.06 (s, 2H), 7.04 (d, *J*=8.0 Hz, 1H), 7.15 (d, *J*=8.0 Hz, 1H), 7.19 (d, *J*=7.3 Hz, 2H), 7.22 - 7.27 (m, 1H), 7.27 - 7.35 (m, 3H), 7.84 (s, 1H).

^{13}C NMR (75 MHz, DMSO-*d*6) δ ppm: 39.7, 52.2, 52.6, 60.3, 118.4, 123.0, 126.4, 127.1, 127.9, 128.2, 128.5, 130.6, 132.0, 137.3, 142.3, 153.0, 164.3, 164.5

HRMS (ESI-TOF) found, m/z: 374.1159 [M+H]$^+$. $C_{20}H_{21}ClNO_4^+$. Calculated, m/z: 374.1154.

Dimethyl 2-(2-(benzyl(methyl)amino)-4-bromobenzylidene)malonate (1g)

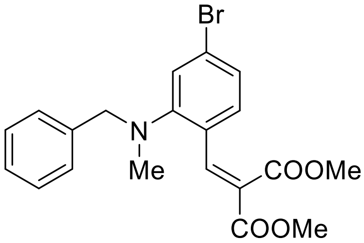

Yield 1.88 g (90%), yellow viscous oil.

^{1}H NMR (700 MHz, DMSO-*d*6) δ ppm: 2.65 (s, 3H), 3.72 (s, 3H), 3.73 (s, 3H), 4.13 (s, 2H), 7.11 - 7.14 (m, 1H), 7.23 - 7.30 (m, 5H), 7.34 (t, *J*=7.4 Hz, 2H), 7.91 (s, 1H).

^{13}C NMR (75 MHz, DMSO-*d*6) δ ppm: 40.4, 52.6, 52.6, 60.5, 122.6, 124.7, 125.1, 125.4, 127.4, 128.1, 128.4, 130.1, 137.1, 140.7, 153.6, 163.9, 165.9.

HRMS (ESI-TOF) found, m/z: 418.0651 [M+H]$^+$. $C_{20}H_{21}BrNO_4^+$. Calculated, m/z: 418.0648.

Dimethyl 2-(2-(benzyl(methyl)amino)-5-bromobenzylidene)malonate (1h)

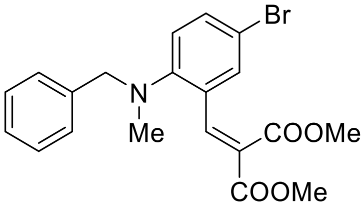

Yield 1.71 g (82%), yellow viscous oil.

^{1}H NMR (700 MHz, DMSO-*d*6) δ ppm: 2.63 (s, 3H), 3.73 (s, 3H), 3.75 (s, 3H), 4.10 (s, 2H), 7.06 (d, *J*=8.8 Hz, 1H), 7.24 (d, *J*=7.3 Hz, 2H), 7.26 (t, *J*=7.3 Hz, 1H), 7.31 (d, *J*=2.3 Hz, 1H), 7.33 (t, *J*=7.4 Hz, 2H), 7.54 (dd, *J*=8.8, 2.3 Hz, 1H), 7.93 (s, 1H).

^{13}C NMR (75 MHz, DMSO-*d*6) δ ppm: 40.5, 52.6, 52.7, 60.5, 113.7, 122.0, 125.8, 127.3, 128.2, 128.4, 128.4, 130.6, 133.7, 137.1, 140.1, 151.5, 163.7, 165.9

HRMS (ESI-TOF) found, m/z: 418.0653 [M+H]$^+$. $C_{20}H_{21}BrNO_4^+$. Calculated, m/z: 418.0648.

Dimethyl 2-(2-(benzyl(methyl)amino)-4-methoxybenzylidene)malonate (1i)

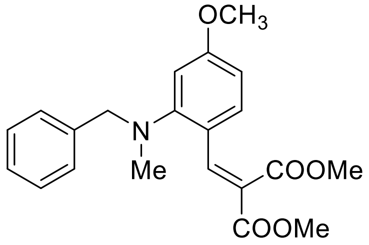

Yield 1.55 g (84%), yellow solid, m.p. 64–66 °C.

^{1}H NMR (700 MHz, DMSO-d_6) δ ppm: 2.63 (s, 3H), 3.72 (s, 3H), 3.73 (s, 3H), 3.77 (s, 3H), 4.10 (s, 2H), 6.63 (d, *J*=2.5 Hz, 1H), 6.67 (dd, *J*=8.7, 2.4 Hz, 1H), 7.19 (d, *J*=8.6 Hz, 1H), 7.25 - 7.31 (m, 3H), 7.34 (t, *J*=7.4 Hz, 2H), 8.01 (s, 1H).

^{13}C NMR (75 MHz, DMSO-d_6) δ ppm: 40.9, 52.4, 52.4, 55.3, 60.9, 105.9, 108.1, 118.7, 122.1, 127.3, 128.2, 128.4, 129.9, 137.5, 140.7, 154.7, 162.1, 164.4, 166.7.

HRMS (ESI-TOF) found, m/z: 370.1657 $[M+H]^+$. $C_{21}H_{24}NO_5^+$. Calculated, m/z: 370.1649.

Dimethyl 2-(2-(benzyl(methyl)amino)-4-(trifluoromethyl)benzylidene)malonate (1j)

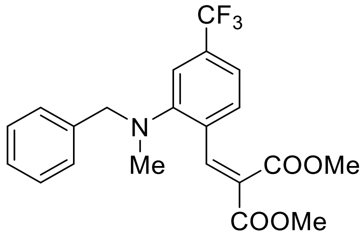

Yield 1.32 g (65%), yellow viscous oil.

^{1}H NMR (700 MHz, DMSO-d_6) δ ppm: 2.70 (s, 3H), 3.72 (s, 3H), 3.74 (s, 3H), 4.18 (s, 2H), 7.25 - 7.29 (m, 3H), 7.32 - 7.36 (m, 3H), 7.39 (s, 2H), 7.96 (s, 1H).

^{13}C NMR (176 MHz, DMSO-d_6) δ ppm: 40.3, 52.5, 52.6, 60.3, 115.8 (q, *J*=3.7 Hz), 118.3 (q, *J*=3.3 Hz), 123.8 (q, *J*=272.7 Hz), 126.5, 127.3, 128.0, 128.3, 129.5, 130.2, 130.9 (q, *J*=31.8 Hz), 137.0, 140.6, 152.5, 163.6, 165.5.

HRMS (ESI-TOF) found, m/z: 408.1423 $[M+H]^+$. $C_{21}H_{21}F_3NO_4^+$. Calculated, m/z: 408.1417.

Dimethyl 2-(2-(benzyl(methyl)amino)-5-(trifluoromethyl)benzylidene)malonate (1k)

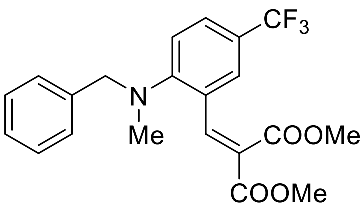

Yield 0.96 g (47%), yellow viscous oil.

^{1}H NMR (700 MHz, DMSO-*d*6) δ ppm: 2.75 (s, 3H), 3.70 (s, 3H), 3.72 (s, 3H), 4.26 (s, 2H), 7.23 (d, *J*=8.6 Hz, 1H), 7.25 (d, *J*=7.1 Hz, 2H), 7.28 (t, *J*=7.3 Hz, 1H), 7.35 (t, *J*=7.5 Hz, 2H), 7.45 (d, *J*=1.3 Hz, 1H), 7.70 (dd, *J*=8.8, 1.9 Hz, 1H), 7.90 (s, 1H).

^{13}C NMR (176 MHz, DMSO-*d*6) δ ppm: 40.2, 52.4, 52.7, 59.9, 119.5, 121.2 (q, *J*=32.5 Hz), 124.2 (q, *J*=271.0 Hz), 125.3, 125.4 (q, *J*=3.7 Hz), 125.8, 127.3, 127.8 (q, *J*=3.7 Hz), 127.8, 128.4, 137.0, 140.8, 155.0, 163.6, 165.8.

HRMS (ESI-TOF) found, m/z: 408.1415 [M+H]$^+$. $C_{21}H_{21}F_3NO_4^+$. Calculated, m/z: 408.1417.

Dimethyl 2-(2-(benzyl(ethyl)amino)-5-bromobenzylidene)malonate (1l)

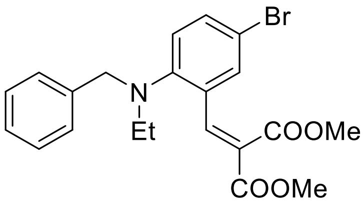

Yield 1.53 g (71%), yellow viscous oil.

^{1}H NMR (700 MHz, DMSO-*d*6) δ ppm: 1.01 (t, *J*=7.0 Hz, 3H), 3.00 (q, *J*=7.0 Hz, 2H), 3.72 (s, 3H), 3.78 (s, 3H), 4.20 (s, 2H), 7.11 (d, *J*=8.8 Hz, 1H), 7.21 (t, *J*=7.3 Hz, 1H), 7.24 (d, *J*=7.1 Hz, 2H), 7.27 - 7.31 (m, 3H), 7.50 (dd, *J*=8.7, 2.4 Hz, 1H), 7.94 (s, 1H).

^{13}C NMR (75 MHz, DMSO-*d*6) δ ppm: 12.1, 48.2, 52.6, 52.8, 56.1, 114.0, 123.7, 125.8, 127.1, 128.1, 128.3, 129.9, 130.5, 133.3, 137.8, 140.0, 149.9, 163.7, 165.9.

HRMS (ESI-TOF) found, m/z: 432.0812 [M+H]$^+$. $C_{21}H_{23}BrNO_4^+$. Calculated, m/z: 432.0805.

Dimethyl 2-(2-(benzyl(ethyl)amino)-4-methoxybenzylidene)malonate (1m)

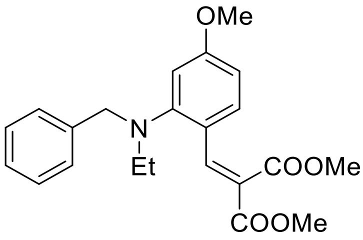

Yield 1.72 g (90%), yellow viscous oil.

^{1}H NMR (700 MHz, DMSO-*d*6) δ ppm: 1.02 (t, *J*=7.0 Hz, 3H), 3.00 (q, *J*=7.0 Hz, 2H), 3.72 (s, 3H), 3.73 (s, 3H), 3.76 (s, 3H), 4.22 (s, 2H), 6.64 (dd, *J*=8.6, 2.5 Hz, 1H), 6.67 (d, *J*=2.5 Hz, 1H), 7.18 (d, *J*=8.6 Hz, 1H), 7.19 - 7.23 (m, 1H), 7.27 - 7.31 (m, 4H), 8.03 (s, 1H).

^{13}C NMR (75 MHz, DMSO-*d*6) δ ppm: 12.0, 48.8, 52.4, 55.3, 56.0, 107.5, 108.4, 120.1, 122.0, 127.0, 128.0, 128.3, 129.7, 138.3, 140.6, 153.0, 161.8, 164.4, 166.8.

HRMS (ESI-TOF) found, m/z: 384.1813 [M+H]$^+$. $C_{22}H_{26}NO_5^+$. Calculated, m/z: 384.1805.

Dimethyl 2-(2-(diethylamino)benzylidene)malonate (1n)

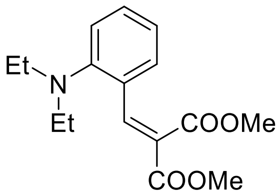

Yield 1.32 g (91%), yellow viscous oil.

^{1}H NMR (700 MHz, DMSO-d_6) δ ppm: 0.98 (t *J*=7.1 Hz, 6H), 3.03 (q, *J*=7.1 Hz, 4H), 3.71 (s, 3H), 3.79 (s, 3H), 7.05 (t, *J*=7.3 Hz, 1H), 7.19 (d, *J*=8.0 Hz, 1H), 7.23 (d, *J*=6.9 Hz, 1H), 7.38 - 7.43 (m, 1H), 7.93 (s, 1H).

^{13}C NMR (75 MHz, DMSO-d_6) δ ppm: 12.4, 47.3, 52.4, 52.5, 121.0, 122.3, 124.1, 128.0, 128.4, 131.0, 141.3, 151.2, 164.2, 166.4.

HRMS (ESI-TOF) found, m/z: 292.1543 $[M+H]^+$. $C_{16}H_{22}NO_4^+$. Calculated, m/z: 292.1543.

Dimethyl 2-(2-(benzyl(methyl)amino)-5-nitrobenzylidene)malonate (1p)

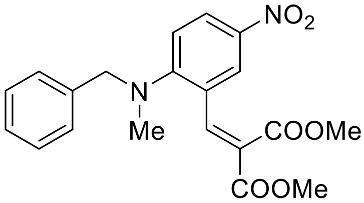

Yield 1.40 g (73%), yellow solid, m.p. 89–91 °C.

^{1}H NMR (700 MHz, DMSO-d_6) δ ppm: 2.89 (s, 3H), 3.69 (s, 3H), 3.75 (s, 3H), 4.46 (s, 2H), 7.15 (d, *J*=9.2 Hz, 1H), 7.23 (d, *J*=7.4 Hz, 2H), 7.29 (t, *J*=7.3 Hz, 1H), 7.36 (t, *J*=7.5 Hz, 2H), 7.78 (s, 1H), 8.02 (d, *J*=2.7 Hz, 1H), 8.16 (dd, *J*=9.2, 2.7 Hz, 1H).

^{13}C NMR (75 MHz, DMSO-d_6) δ ppm: 40.3, 52.6, 52.7, 59.4, 118.0, 122.8, 125.0, 125.7, 126.2, 127.4, 128.6, 136.8, 139.2, 141.1, 156.8, 163.5, 165.6.

HRMS (ESI-TOF) found, m/z: 385.1399 $[M+H]^+$. $C_{20}H_{21}N_2O_6^+$. Calculated, m/z: 385.1394.

Dimethyl 2-(2-(dimethylamino)benzylidene)malonate (1r)

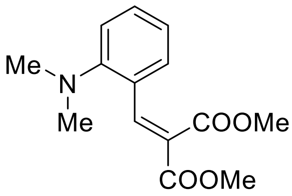

Yield 1.20 g (91%), yellow solid, m.p. 79–81 °C.

^{1}H NMR (700 MHz, DMSO-d_6) δ ppm: 2.70 (s, 6 H), 3.71 (s, 3 H), 3.79 (s, 3 H), 6.99 - 7.04 (m, 1 H), 7.13 (d, *J*=7.6 Hz, 1 H), 7.20 (dd, *J*=7.8, 1.3 Hz, 1 H), 7.37 - 7.42 (m, 1 H), 7.85 (s, 1 H).

^{13}C NMR (75 MHz, DMSO-d_6) δ ppm: 44.5, 52.4, 52.6, 118.2, 121.8, 124.0, 125.6, 128.5, 131.5, 141.3, 153.6, 164.3, 166.3.

HRMS (ESI-TOF) found, m/z: 264.1232 $[M+H]^+$. $C_{14}H_{18}NO_4^+$. Calculated, m/z: 264.1230.

4. NMR study on the reaction intermediates

1a (20.0 mg, 0.059 mmol, 1 eq.) was dissolved in dry degassed $C_2H_4Cl_2$ (0.6 mL) in a standard NMR tube in argon. Then freshly distilled $BF_3 \cdot Et_2O$ (16.7 mg, 0.118 mmol, 2 eq.) was carefully added and the reaction vessel was sealed. The resulted solution was shaken for 3 min and the sample was stored for 24 h at 25 °C. ^{1}H NMR spectrum of the reaction mixture was recorded on a 300 MHz Bruker Avance Neo at 293 K without any additional deuterated solvents. ^{11}B and ^{19}F NMR spectra were recorded on a 300 MHz Bruker Avance III-HD at 293 K. 3D shimming was made on $C_2H_4Cl_2$ signal.

We also recorded ^{1}H NMR spectrum of **1a** in $C_2H_4Cl_2$ and ^{11}B and ^{19}F NMR spectra of $BF_3 \cdot Et_2O$ in $C_2H_4Cl_2$ in the similar manner.

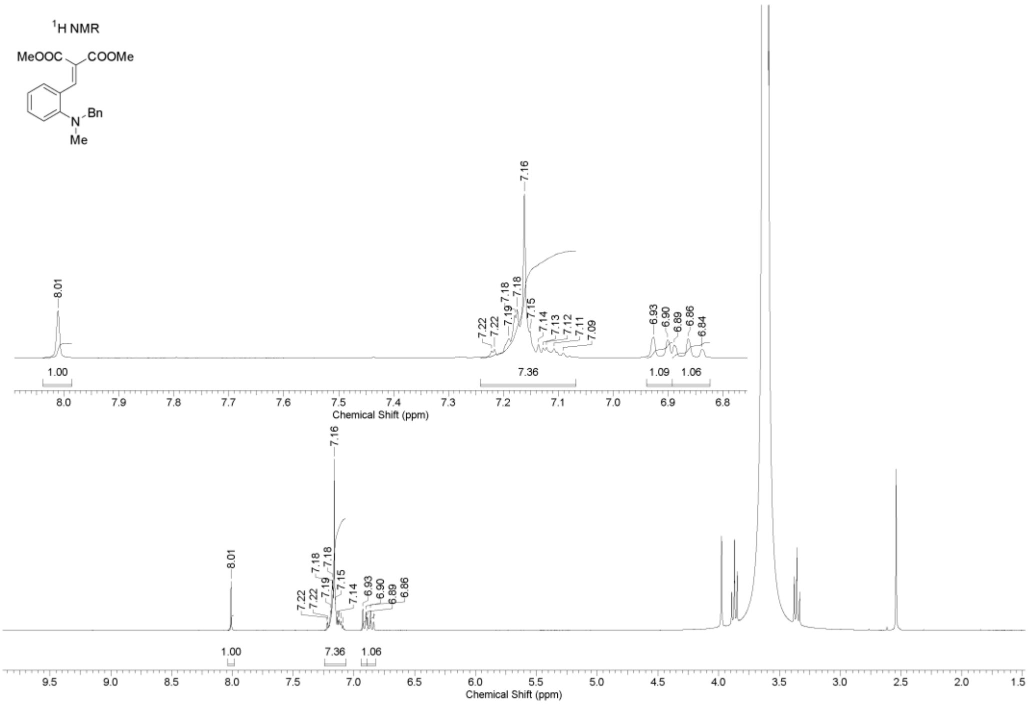

Figure S1. ^{1}H NMR spectrum of the compound **1a** in $C_2H_4Cl_2$.

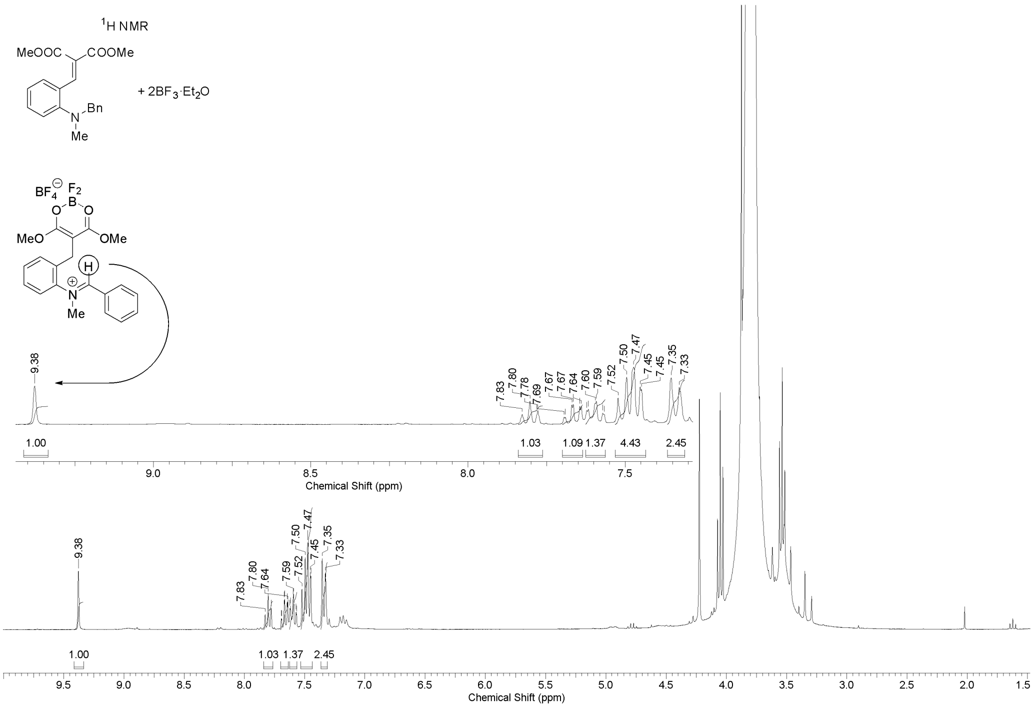

Figure S2. ^{1}H NMR spectrum of the reaction mixture in $C_2H_4Cl_2$.

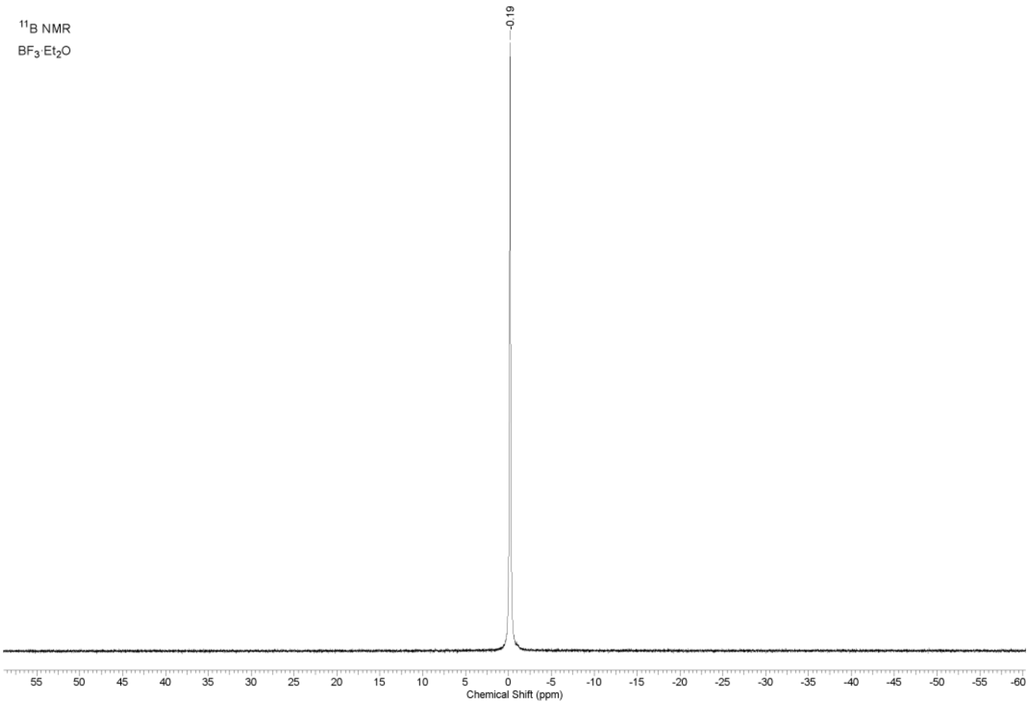

Figure S3. ^{11}B NMR spectrum of BF_3 in $C_2H_4Cl_2$.

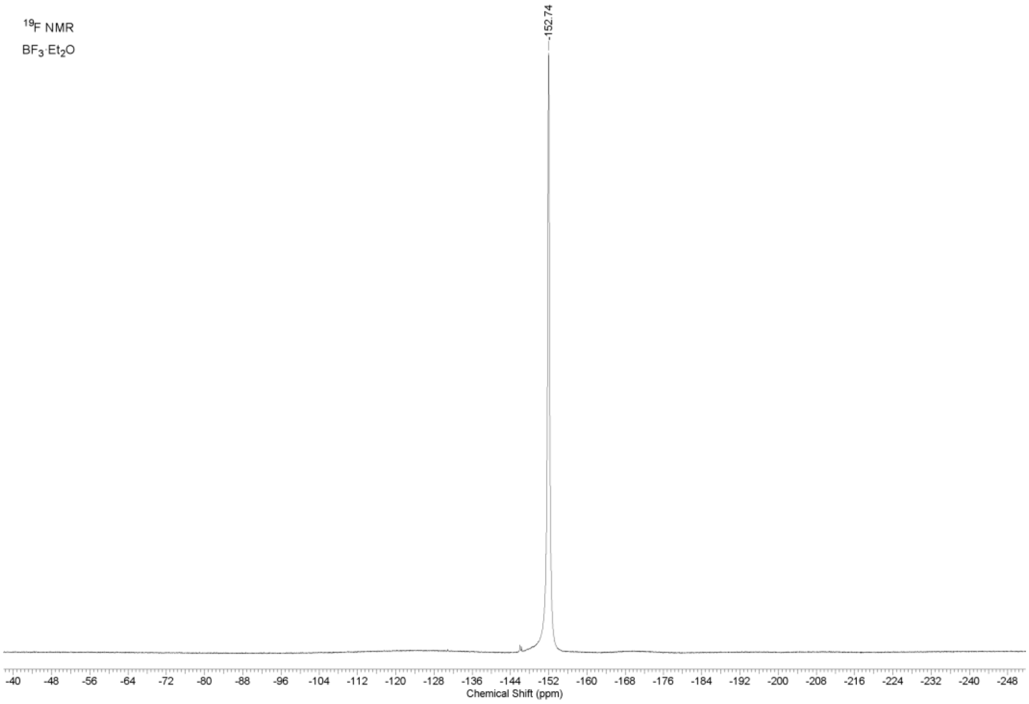

Figure S4. ^{19}F NMR spectrum of BF_3 in $C_2H_4Cl_2$.

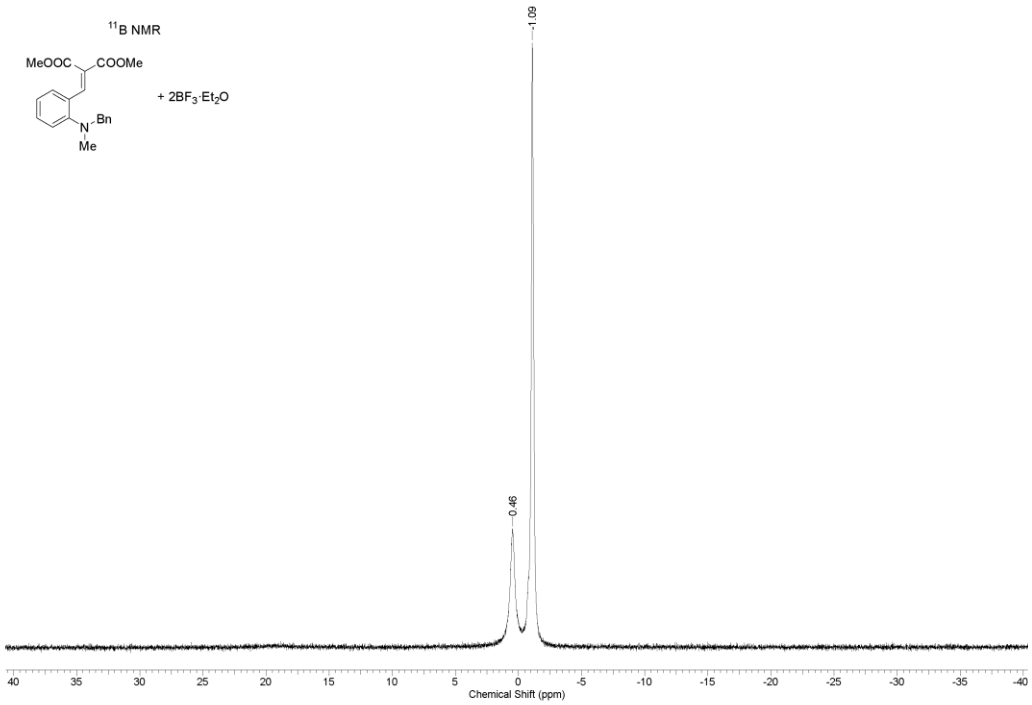

Figure S5. ^{11}B NMR spectrum of the reaction mixture in $C_2H_4Cl_2$.

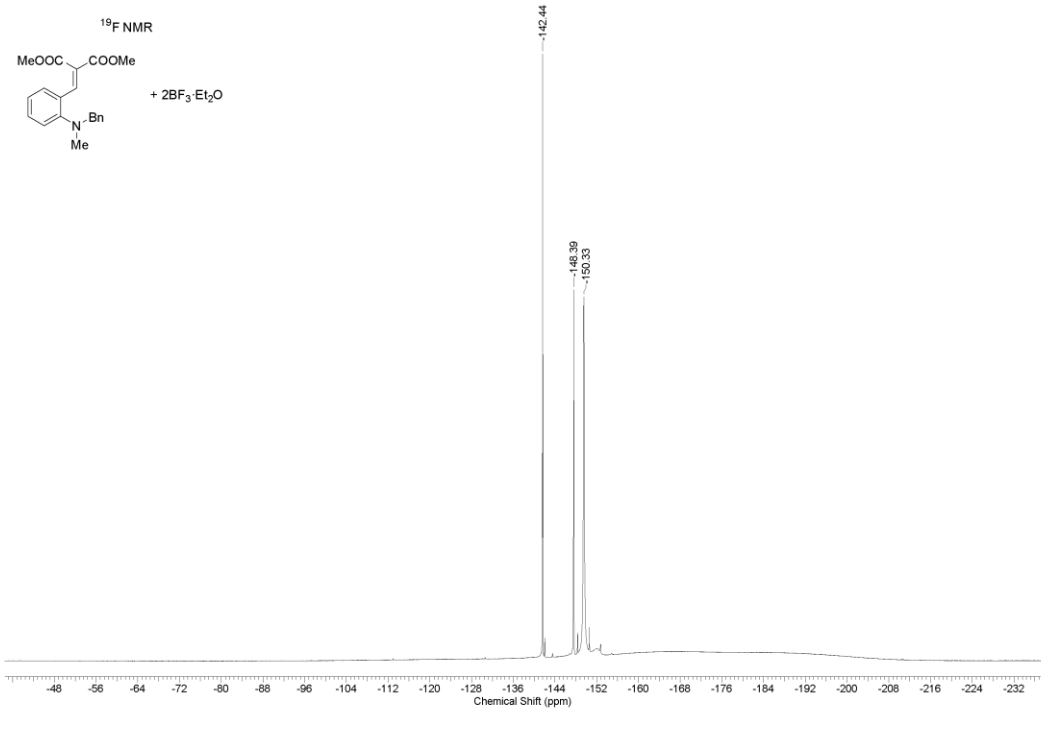

Figure S6. ^{19}F NMR spectrum of the reaction mixture in $C_2H_4Cl_2$.

5. References

[1] Yang, P.; Xu, W.; Wang, R.; Zhang, M.; Xie, C.; Zeng, X.; Wang, M., *Org. Lett.* **2019**, *21*, 3658–3662.

[2] Murarka, S.; Deb, I.; Zhang, C.; Seidel, D., *J. Am. Chem. Soc.* **2009**, *131*, 13226–13227.

[3] Zaitseva, E. R.; Smirnov, A. Y.; Myasnyanko, I. N.; Mineev, K. S.; Sokolov, A. I.; Volkhina, T. N.; Mikhaylov, A. A.; Baleeva, N. S.; Baranov, M. S., *New J. Chem.* **2021**, *45*, 1805–1808.

[4] Josa-Culleré, L.; Hirst, M. G.; Lockett, J. P.; Thompson, A. L.; Moloney, M. G., *J. Org. Chem.* **2019**, *84*, 9671–9683.

[5] Rosevear, J.; Wilshire, J. F. K., *Aust. J. Chem.* **1981**, *34*, 839.

[6] Mori, K.; Ehara, K.; Kurihara, K.; Akiyama, T., *J. Am. Chem. Soc.* **2011**, *133*, 6166–6169.

6. Copies of ^{1}H and ^{13}C NMR spectra

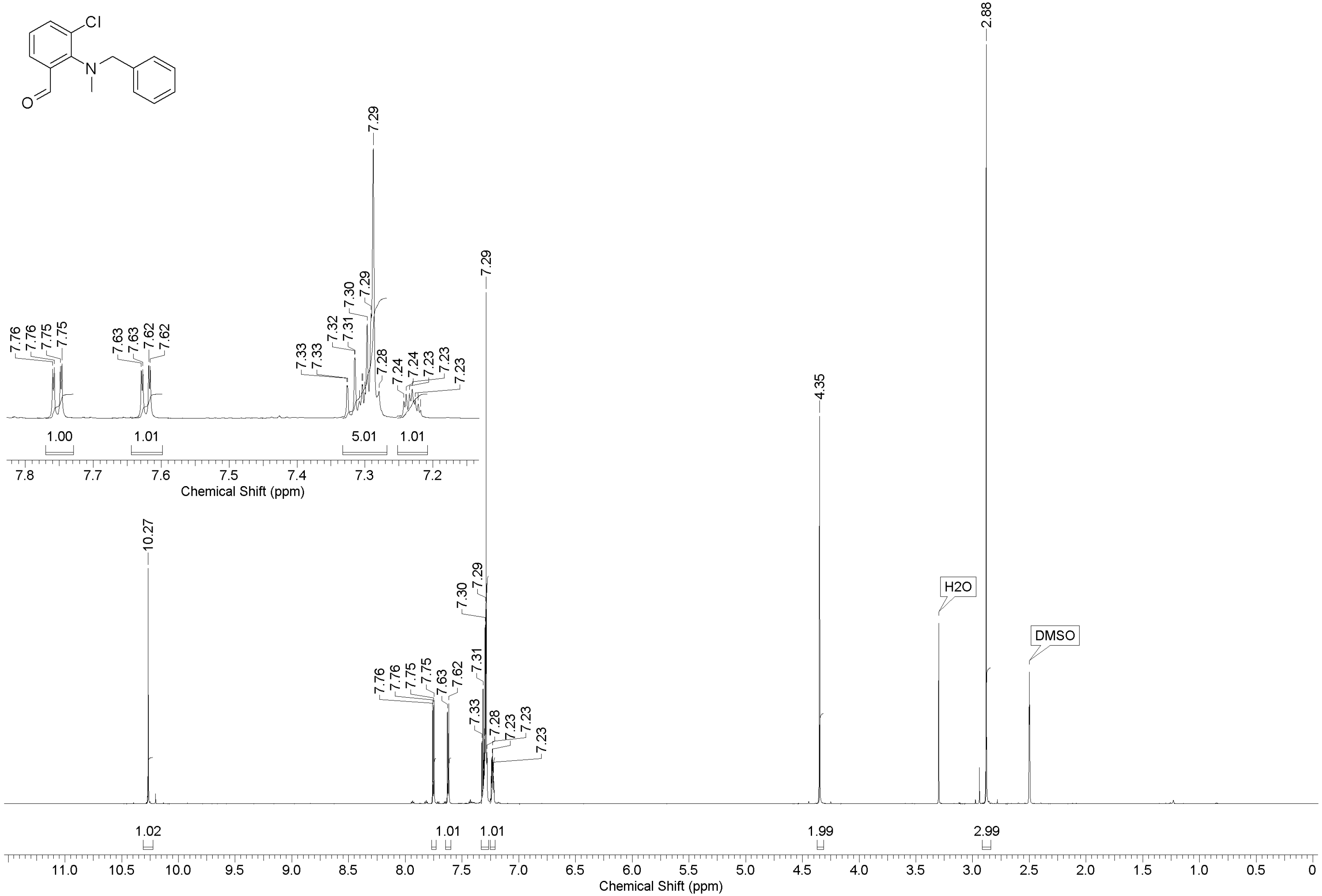

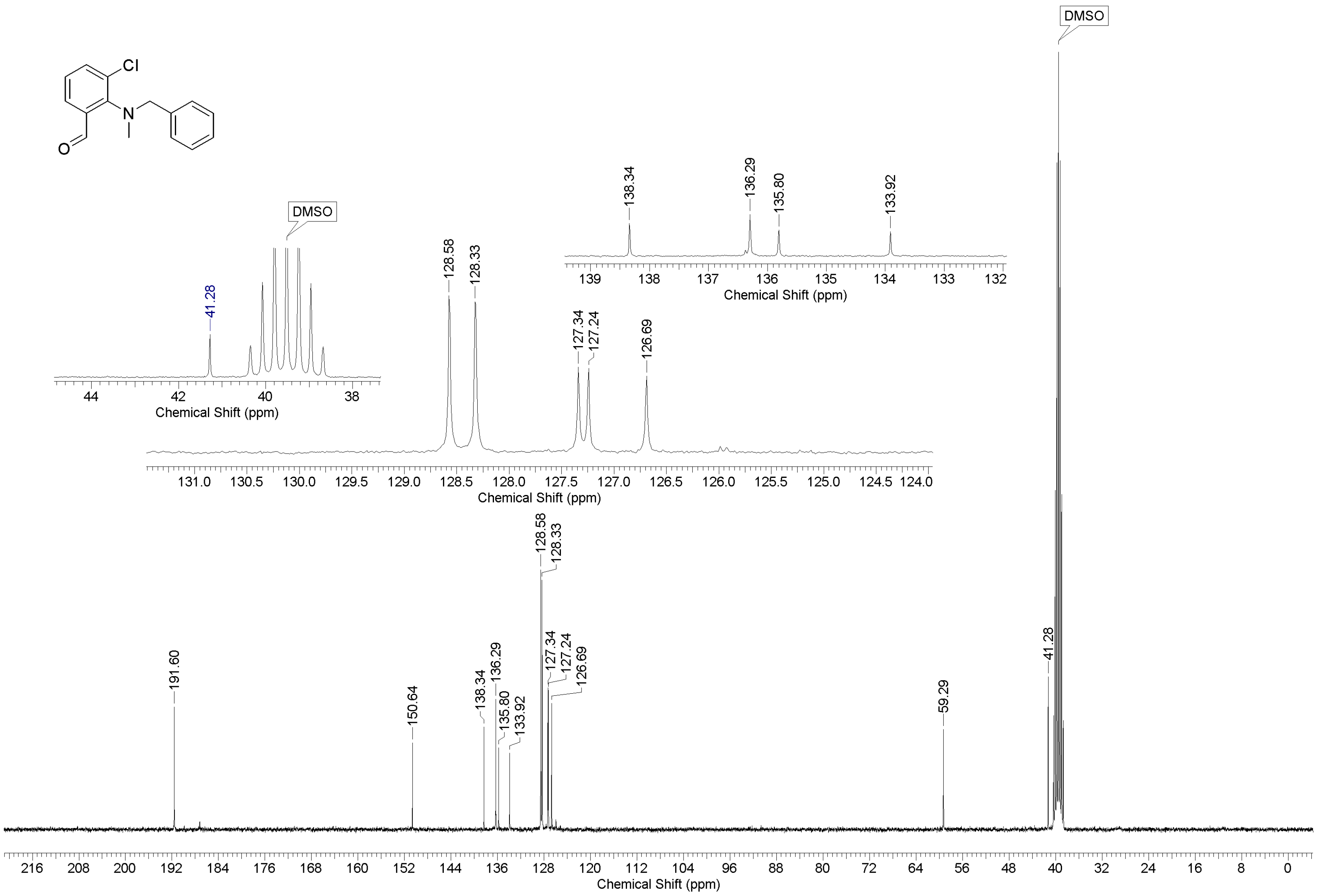
Cl
N
O
DMSO
41.28
Chemical Shift (ppm)
128.58
128.33
127.34
127.24
126.69
138.34
136.29
135.80
133.92
191.60
150.64
59.29

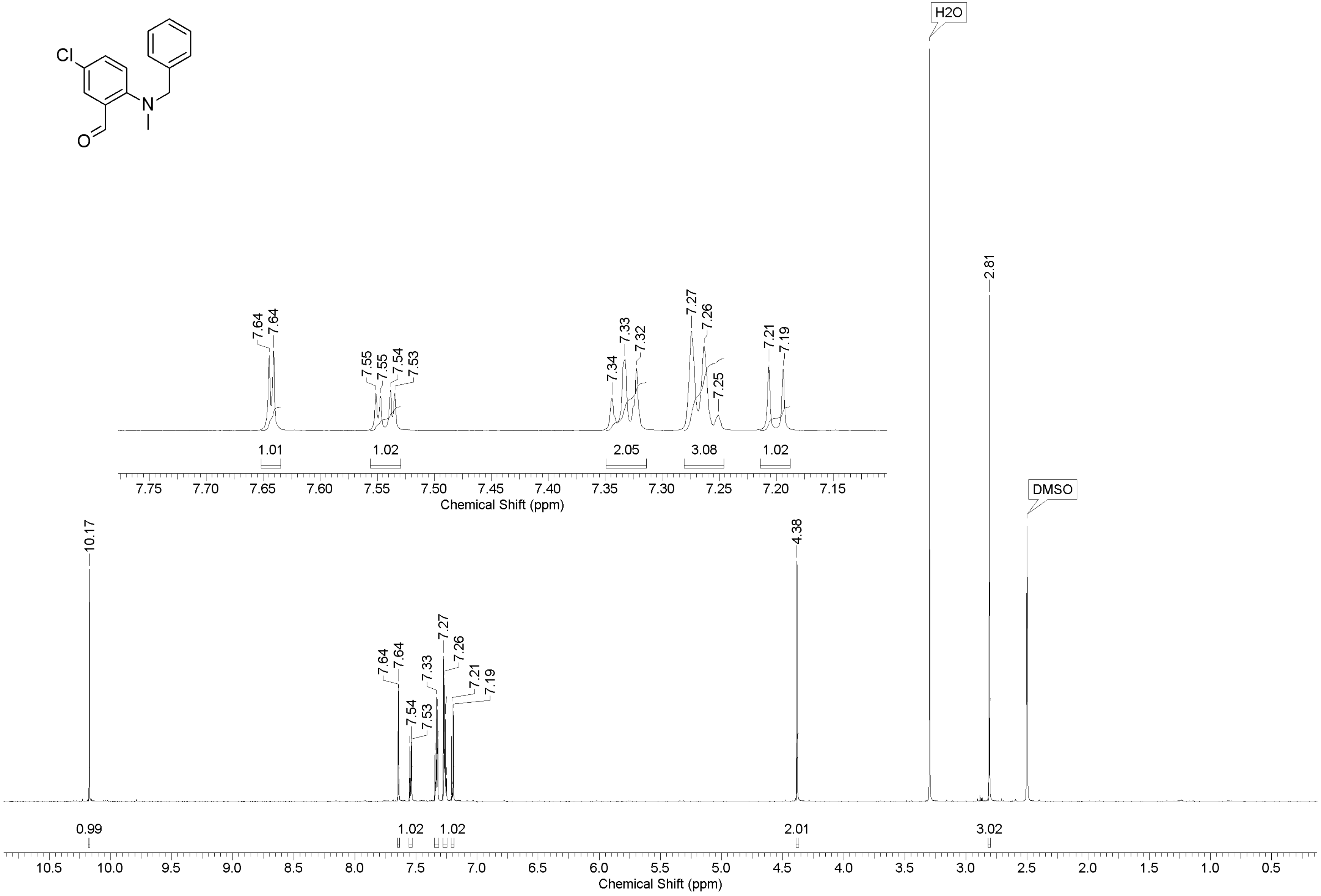

Cl
N
O
H2O
DMSO
10.17
7.64
7.64
7.54
7.53
7.33
7.27
7.26
7.21
7.19
4.38
2.81
7.55
7.55
7.34
7.32
7.25
0.99
1.02
1.02
2.01
3.02
1.01
1.02
2.05
3.08
1.02
Chemical Shift (ppm)
Chemical Shift (ppm)

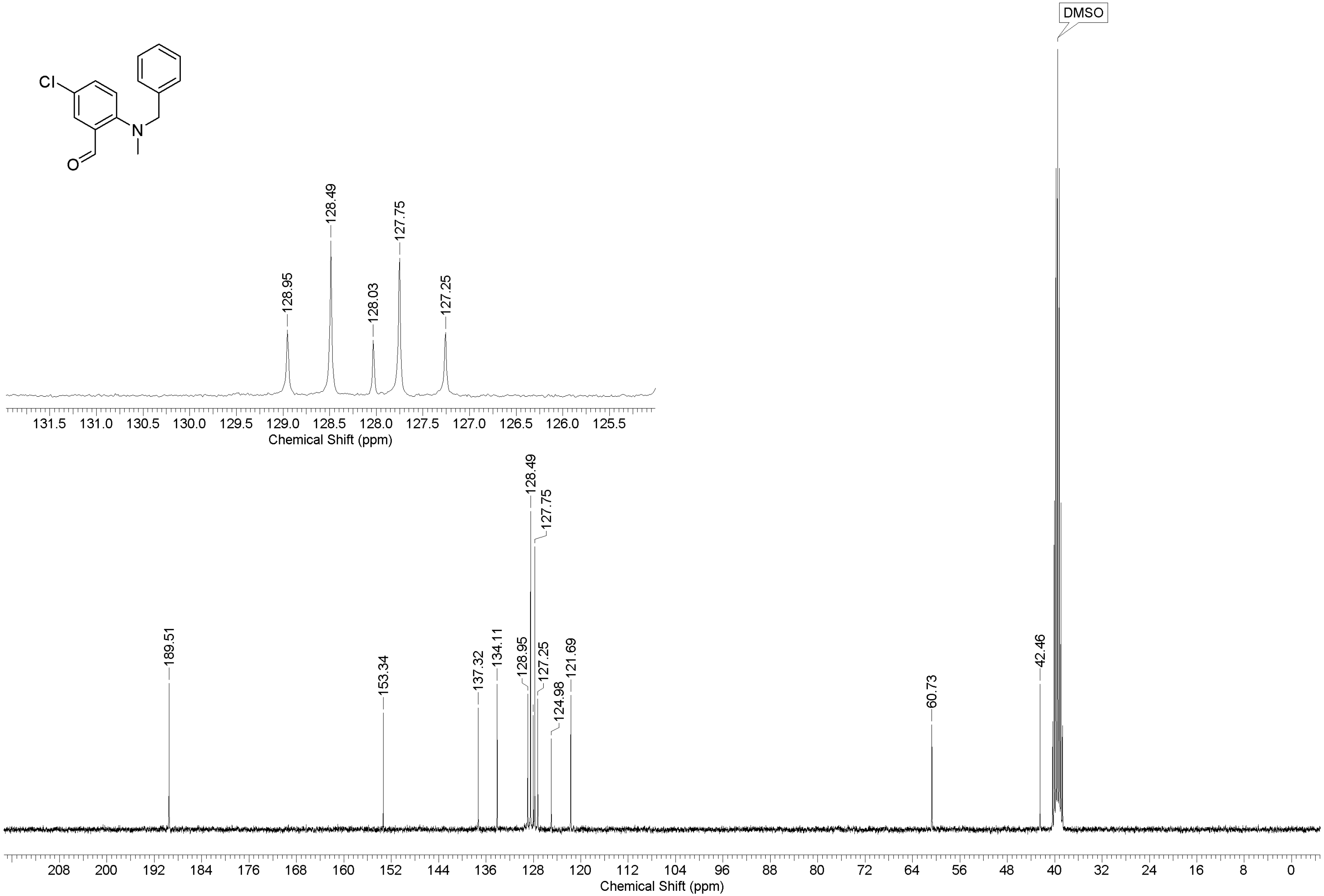

Cl
N
O
DMSO
128.95
128.49
128.03
127.75
127.25
131.5 131.0 130.5 130.0 129.5 129.0 128.5 128.0 127.5 127.0 126.5 126.0 125.5
Chemical Shift (ppm)
189.51
153.34
137.32
134.11
128.95
128.49
127.75
127.25
124.98
121.69
60.73
42.46
208 200 192 184 176 168 160 152 144 136 128 120 112 104 96 88 80 72 64 56 48 40 32 24 16 8 0
Chemical Shift (ppm)

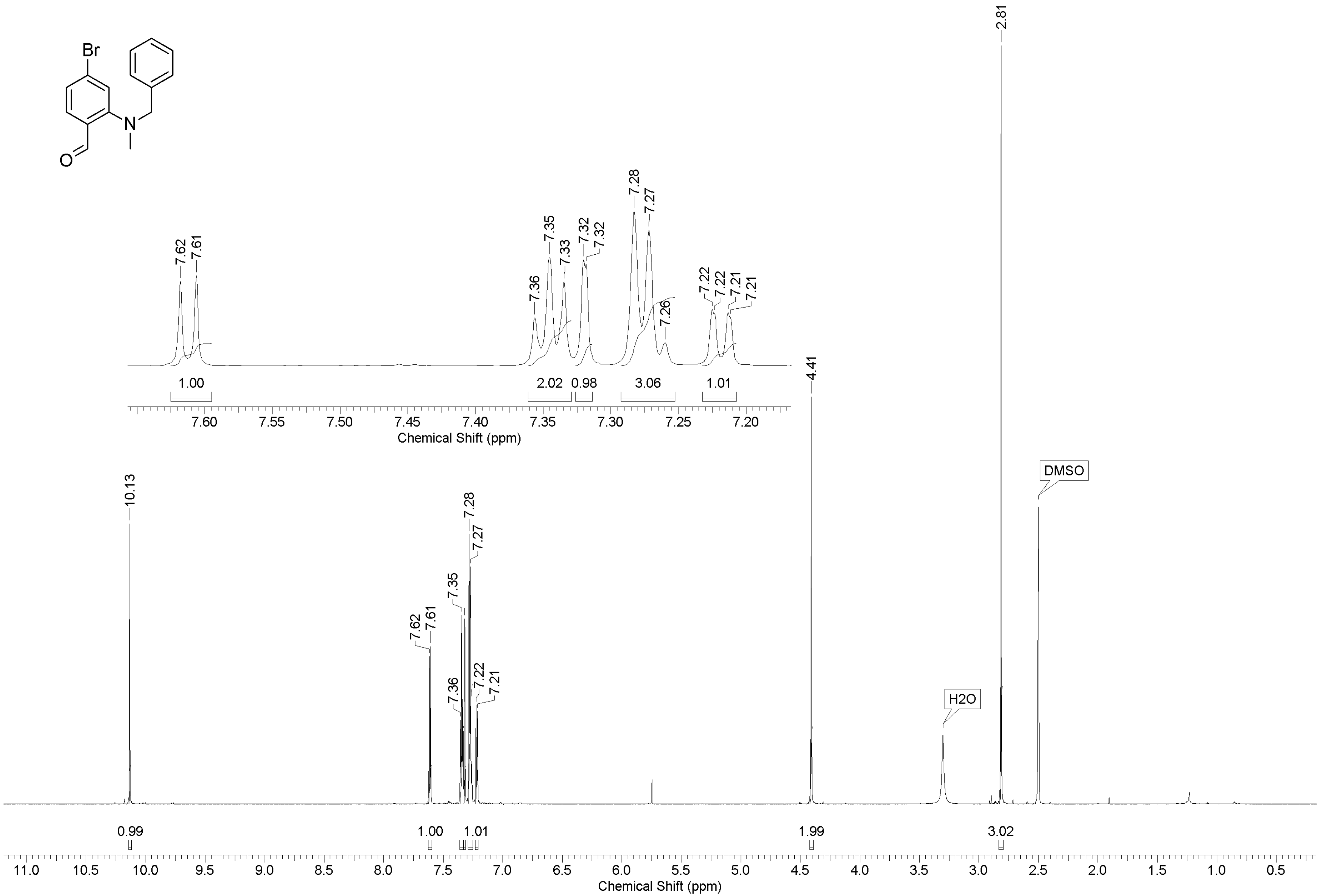
Br
N
O
7.62
7.61
7.36
7.35
7.33
7.32
7.32
7.28
7.27
7.26
7.22
7.22
7.21
7.21
1.00
2.02
0.98
3.06
1.01
7.60 7.55 7.50 7.45 7.40 7.35 7.30 7.25 7.20
Chemical Shift (ppm)
10.13
4.41
2.81
DMSO
H2O
0.99
1.00
1.01
1.99
3.02
11.0 10.5 10.0 9.5 9.0 8.5 8.0 7.5 7.0 6.5 6.0 5.5 5.0 4.5 4.0 3.5 3.0 2.5 2.0 1.5 1.0 0.5
Chemical Shift (ppm)

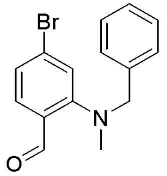

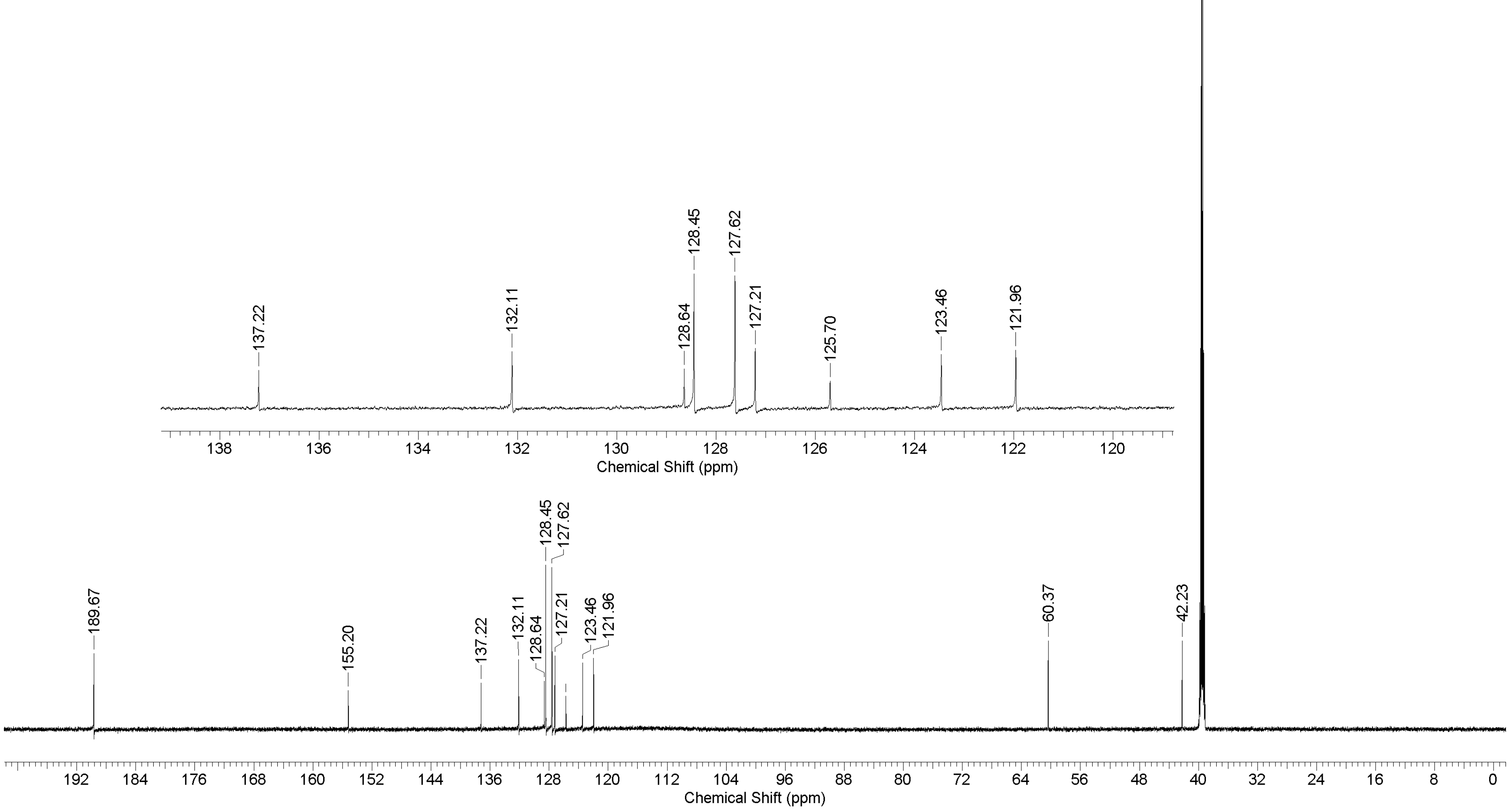

DMSO
137.22
132.11
128.64
128.45
127.62
127.21
125.70
123.46
121.96
Chemical Shift (ppm)
189.67
155.20
60.37
42.23
Chemical Shift (ppm)

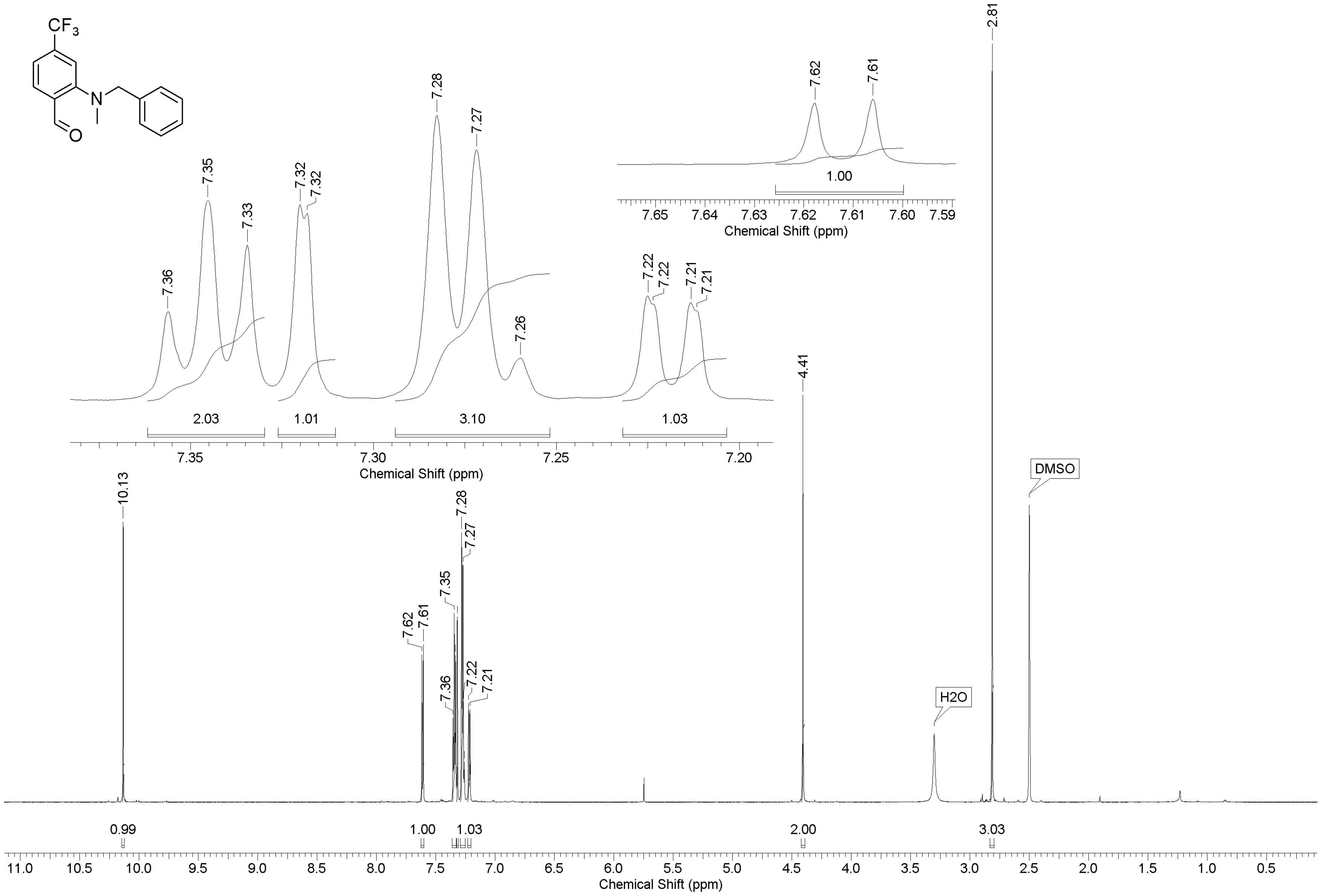

CF3
N
O
7.62
7.61
1.00
7.65 7.64 7.63 7.62 7.61 7.60 7.59
Chemical Shift (ppm)
7.36
7.35
7.33
7.32
7.32
7.28
7.27
7.26
7.22
7.22
7.21
7.21
2.03
1.01
3.10
1.03
7.35 7.30 7.25 7.20
Chemical Shift (ppm)
2.81
4.41
10.13
DMSO
H2O
0.99
1.00
1.03
2.00
3.03
11.0 10.5 10.0 9.5 9.0 8.5 8.0 7.5 7.0 6.5 6.0 5.5 5.0 4.5 4.0 3.5 3.0 2.5 2.0 1.5 1.0 0.5
Chemical Shift (ppm)

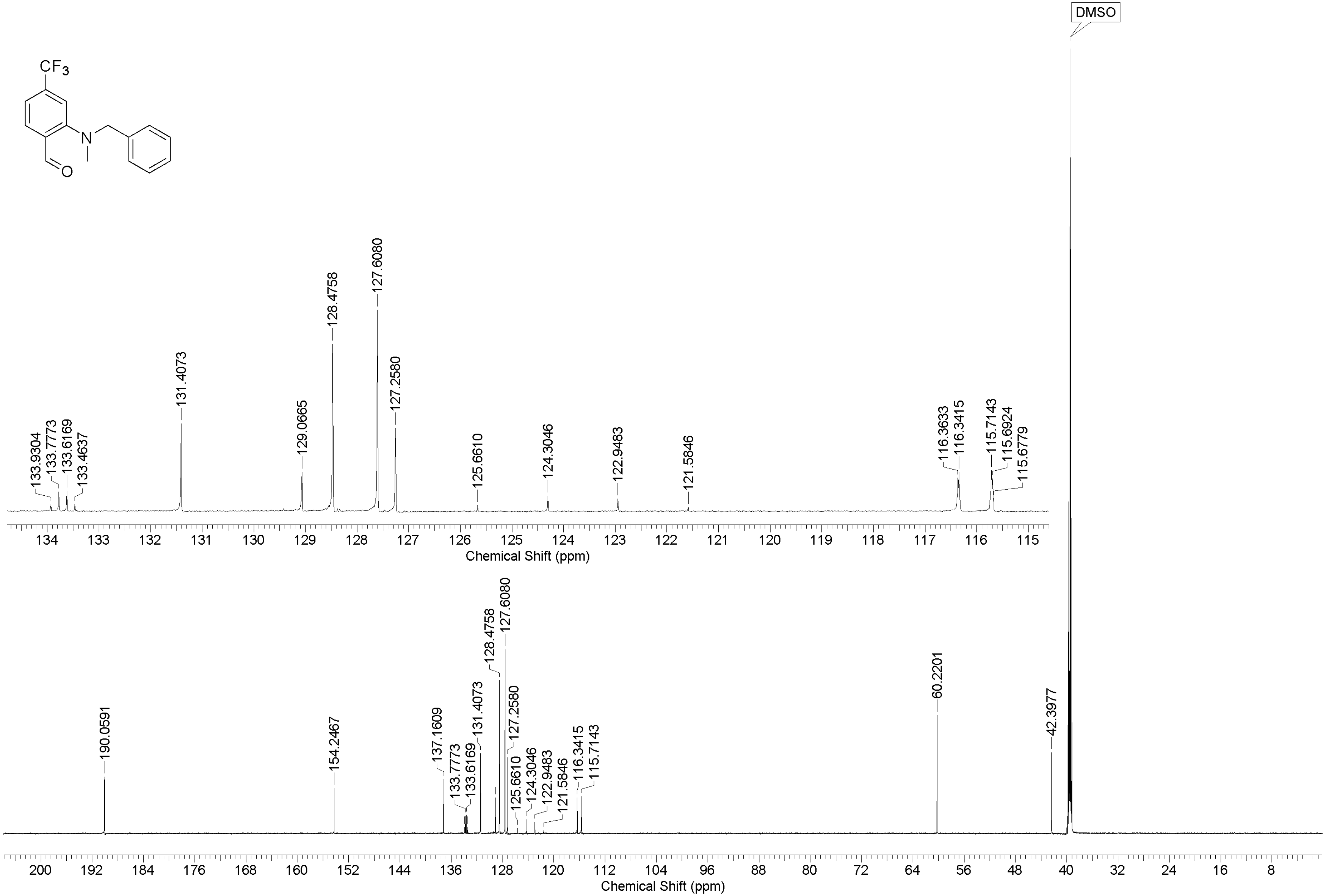
CF3
N
O
DMSO
133.9304
133.7773
133.6169
133.4637
131.4073
129.0665
128.4758
127.6080
127.2580
125.6610
124.3046
122.9483
121.5846
116.3633
116.3415
115.7143
115.6924
115.6779
Chemical Shift (ppm)
190.0591
154.2467
137.1609
133.7773
133.6169
131.4073
128.4758
127.6080
127.2580
125.6610
124.3046
122.9483
121.5846
116.3415
115.7143
60.2201
42.3977
Chemical Shift (ppm)

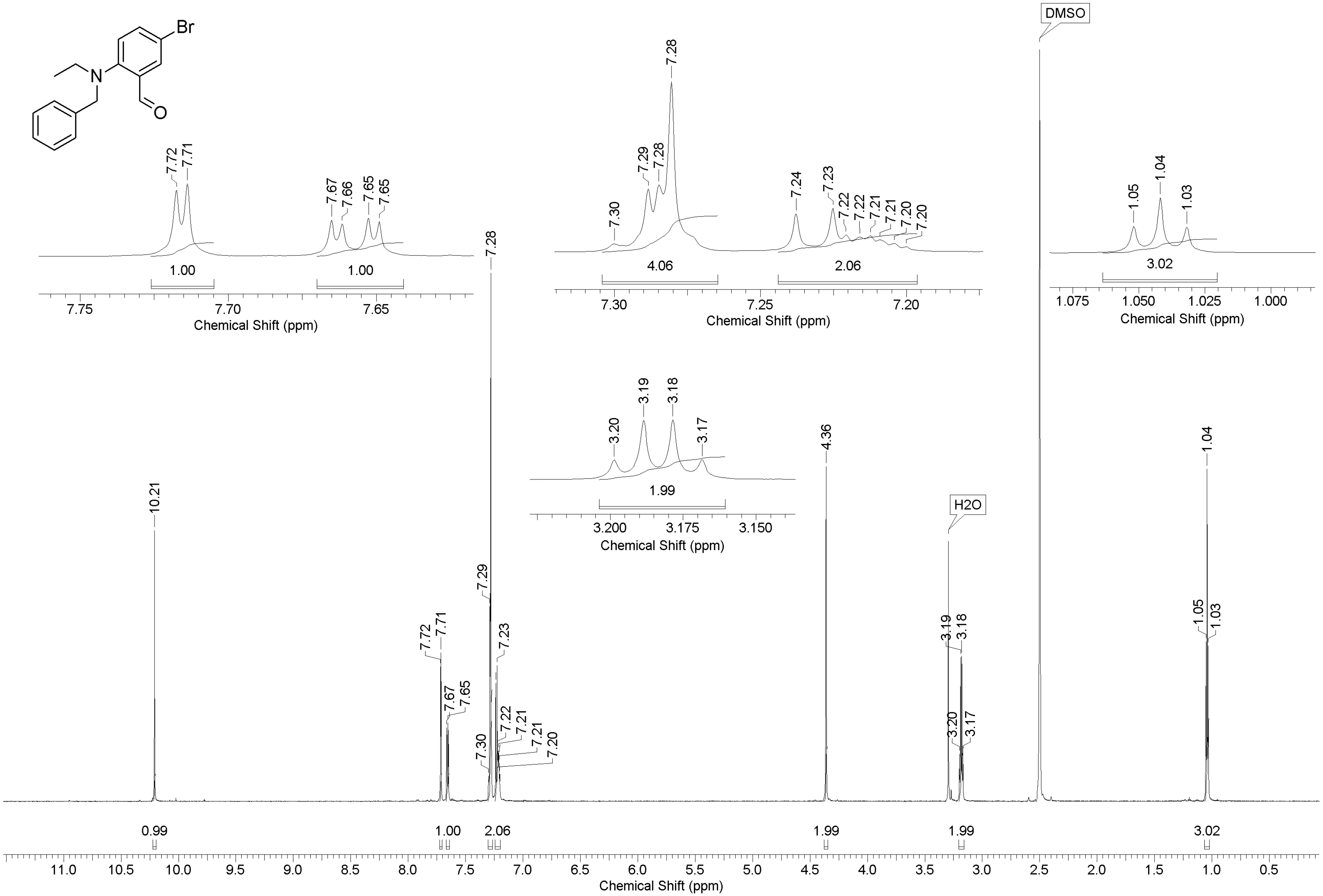
Br
N
O
DMSO
H2O
10.21
7.72
7.71
7.67
7.65
7.30
7.29
7.28
7.23
7.22
7.21
7.21
7.20
4.36
3.20
3.19
3.18
3.17
1.05
1.04
1.03
0.99
1.00
2.06
1.99
1.99
3.02
4.06
Chemical Shift (ppm)

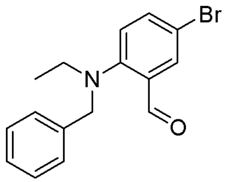

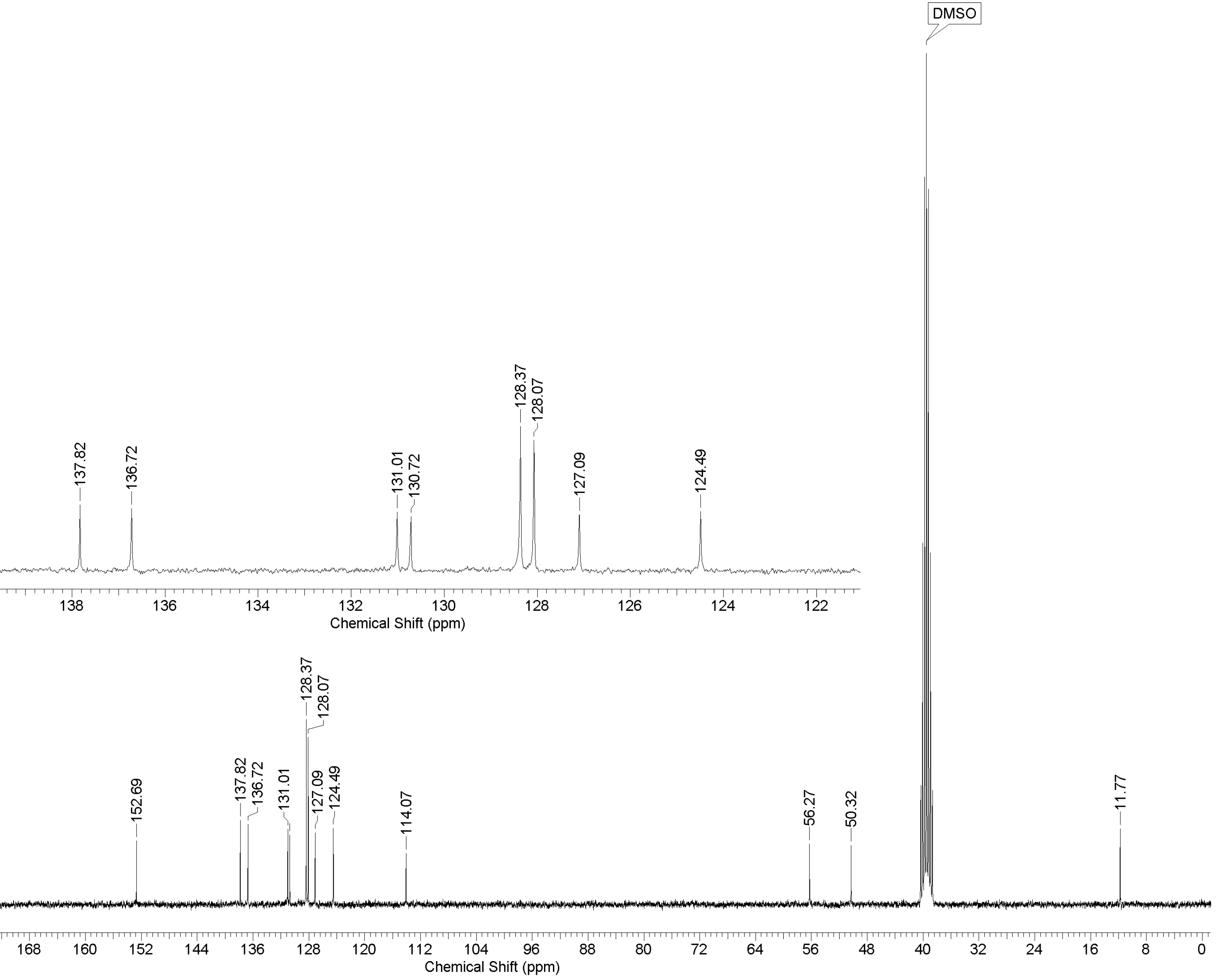
DMSO
137.82
136.72
131.01
130.72
128.37
128.07
127.09
124.49
140
138
136
134
132
130
128
126
124
122
Chemical Shift (ppm)
189.72
152.69
114.07
56.27
50.32
11.77
200
192
184
176
168
160
152
144
136
128
120
112
104
96
88
80
72
64
56
48
40
32
24
16
8
0
Chemical Shift (ppm)

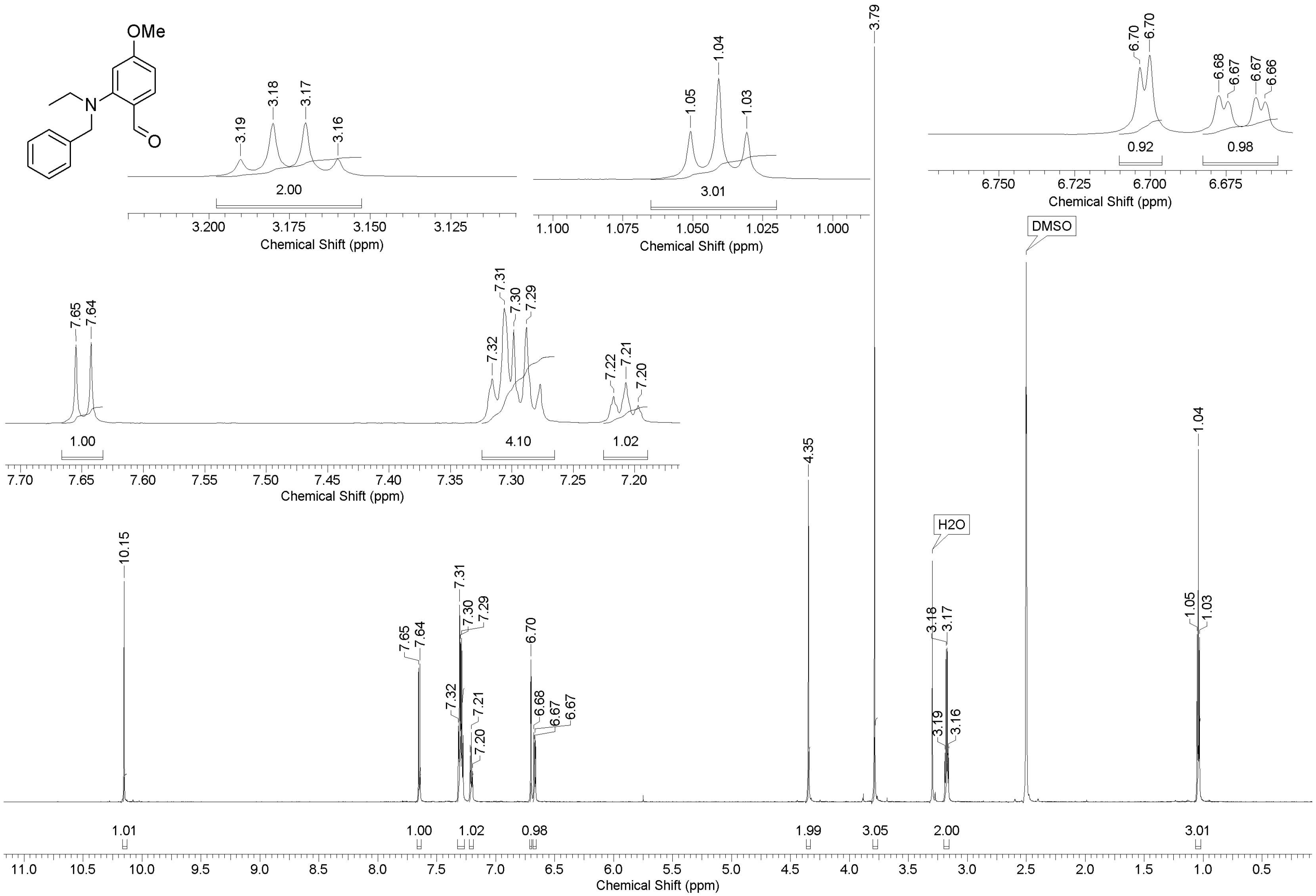
OMe
N
O
3.19
3.18
3.17
3.16
2.00
Chemical Shift (ppm)
1.05
1.04
1.03
3.01
Chemical Shift (ppm)
3.79
6.70
6.70
6.68
6.67
6.67
6.66
0.92
0.98
Chemical Shift (ppm)
DMSO
7.65
7.64
7.32
7.31
7.30
7.29
7.22
7.21
7.20
1.00
4.10
1.02
Chemical Shift (ppm)
4.35
H2O
10.15
1.04
1.01
1.00
1.02
0.98
1.99
3.05
2.00
3.01
Chemical Shift (ppm)

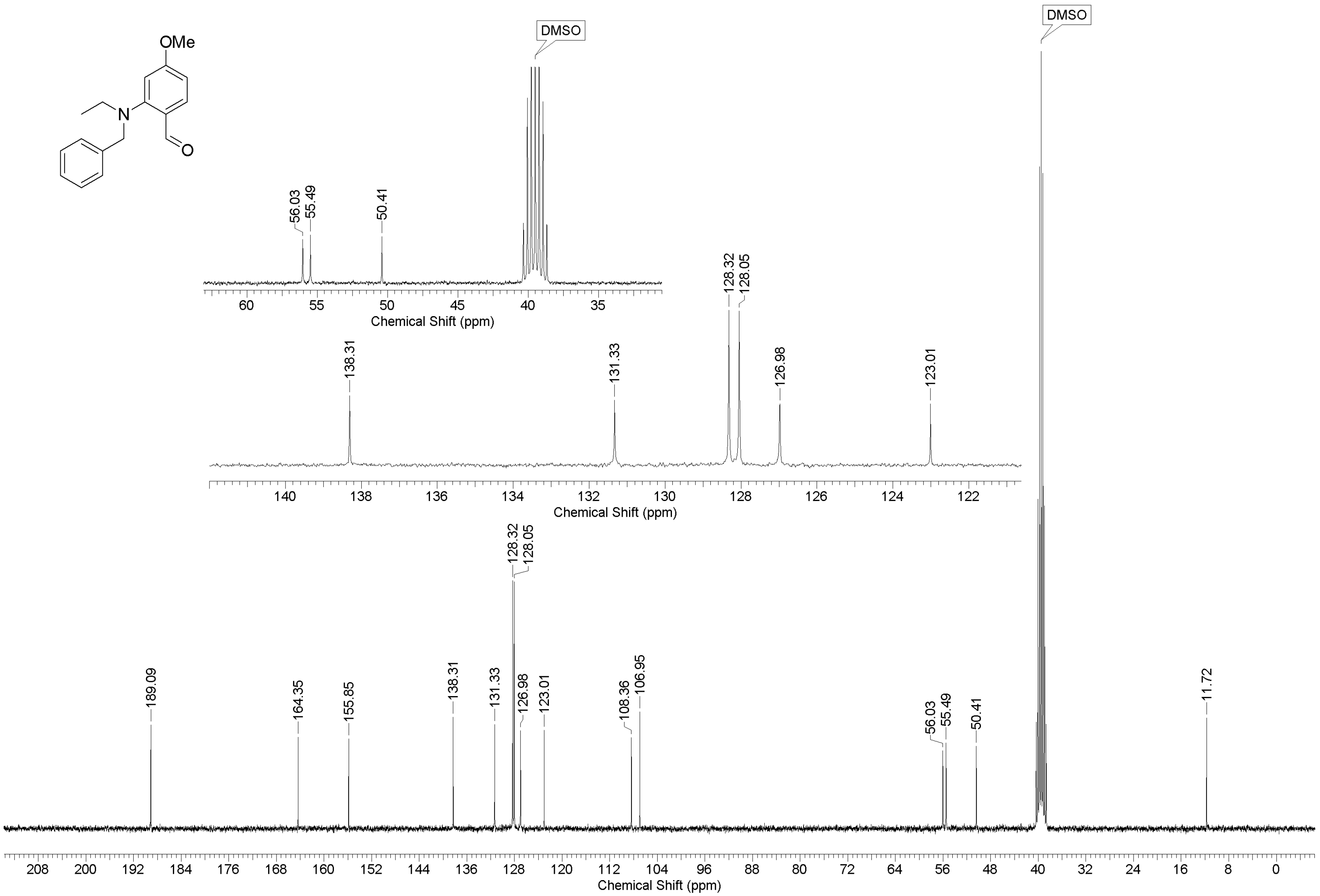
OMe
N
O
DMSO
56.03
55.49
50.41
Chemical Shift (ppm)
138.31
131.33
128.32
128.05
126.98
123.01
Chemical Shift (ppm)
DMSO
189.09
164.35
155.85
138.31
131.33
128.32
128.05
126.98
123.01
108.36
106.95
56.03
55.49
50.41
11.72
Chemical Shift (ppm)

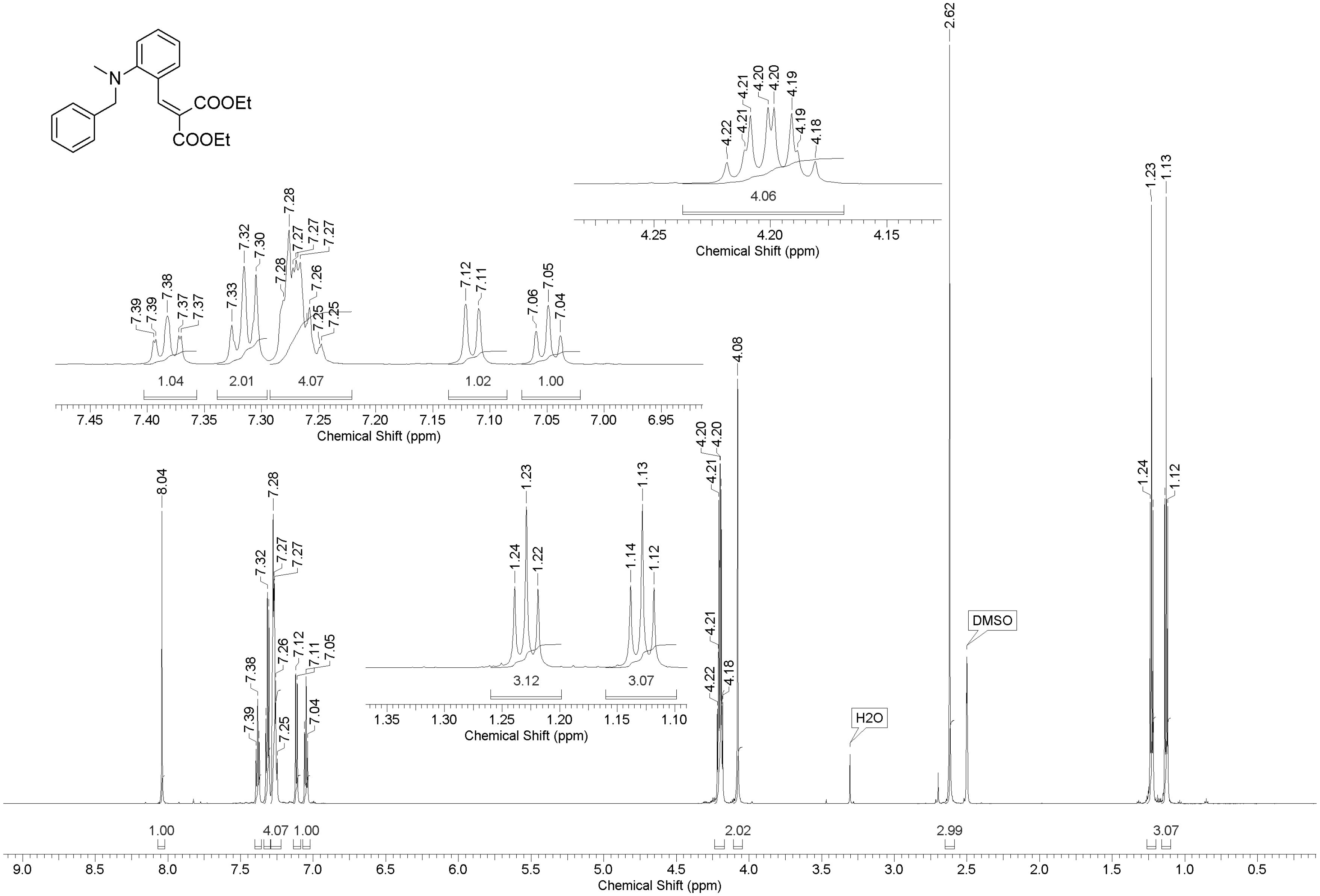

N
COOEt
COOEt
2.62
4.08
8.04
7.28
7.32
7.27
7.27
7.38
7.39
7.26
7.25
7.12
7.11
7.05
7.04
4.20
4.20
4.21
4.21
4.22
4.18
1.23
1.13
1.24
1.12
H2O
DMSO
1.00
4.07
1.00
2.02
2.99
3.07
Chemical Shift (ppm)
9.0
8.5
8.0
7.5
7.0
6.5
6.0
5.5
5.0
4.5
4.0
3.5
3.0
2.5
2.0
1.5
1.0
0.5
4.22
4.21
4.21
4.20
4.20
4.19
4.19
4.18
4.06
4.25
4.20
4.15
Chemical Shift (ppm)
7.39
7.39
7.38
7.37
7.37
7.33
7.32
7.30
7.28
7.28
7.27
7.27
7.27
7.26
7.25
7.25
7.12
7.11
7.06
7.05
7.04
1.04
2.01
4.07
1.02
1.00
7.45
7.40
7.35
7.30
7.25
7.20
7.15
7.10
7.05
7.00
6.95
Chemical Shift (ppm)
1.24
1.23
1.22
1.14
1.13
1.12
3.12
3.07
1.35
1.30
1.25
1.20
1.15
1.10
Chemical Shift (ppm)

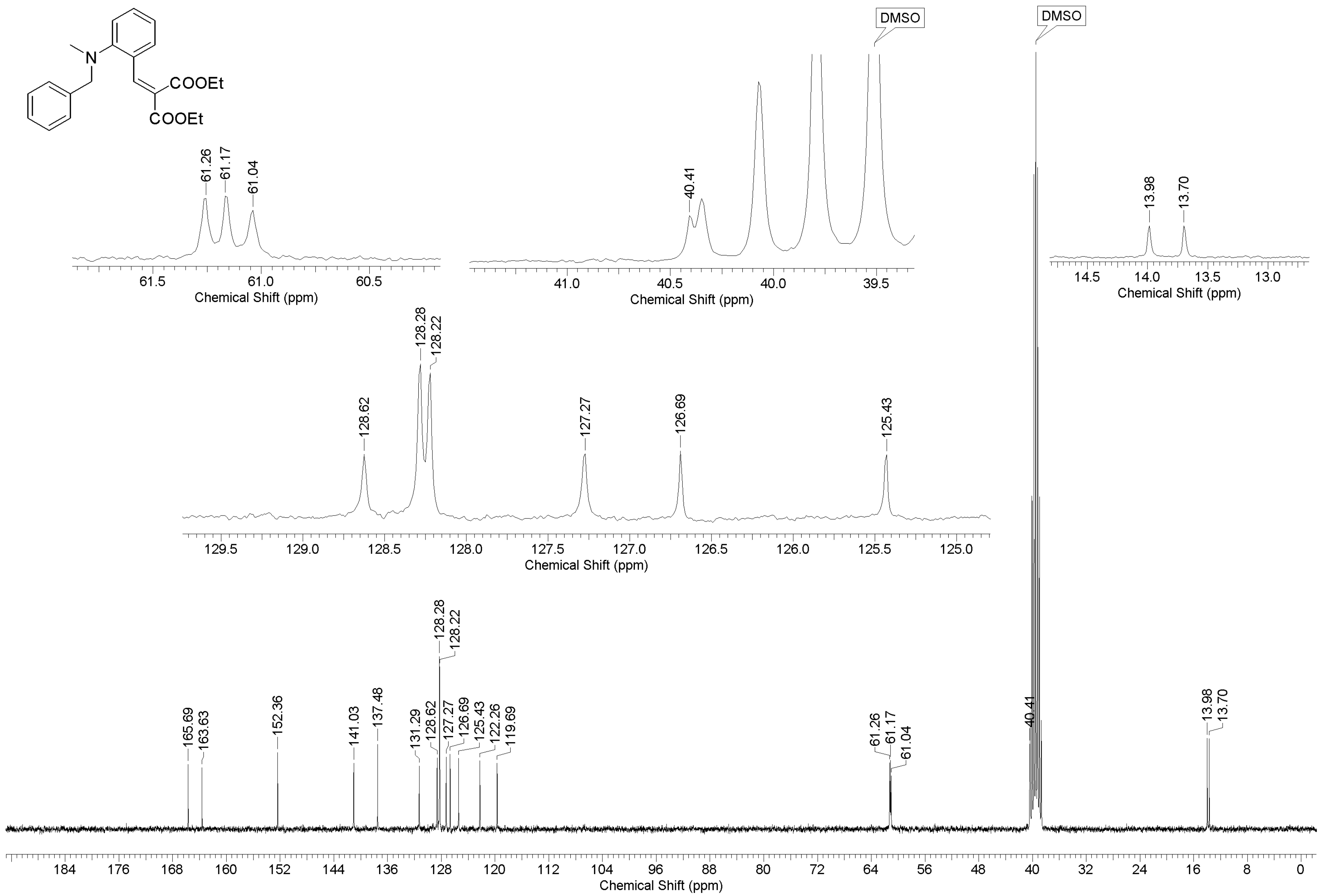

COOEt
COOEt
N
DMSO
DMSO
61.26
61.17
61.04
Chemical Shift (ppm)
40.41
13.98
13.70
128.62
128.28
128.22
127.27
126.69
125.43
165.69
163.63
152.36
141.03
137.48
131.29
122.26
119.69
Chemical Shift (ppm)

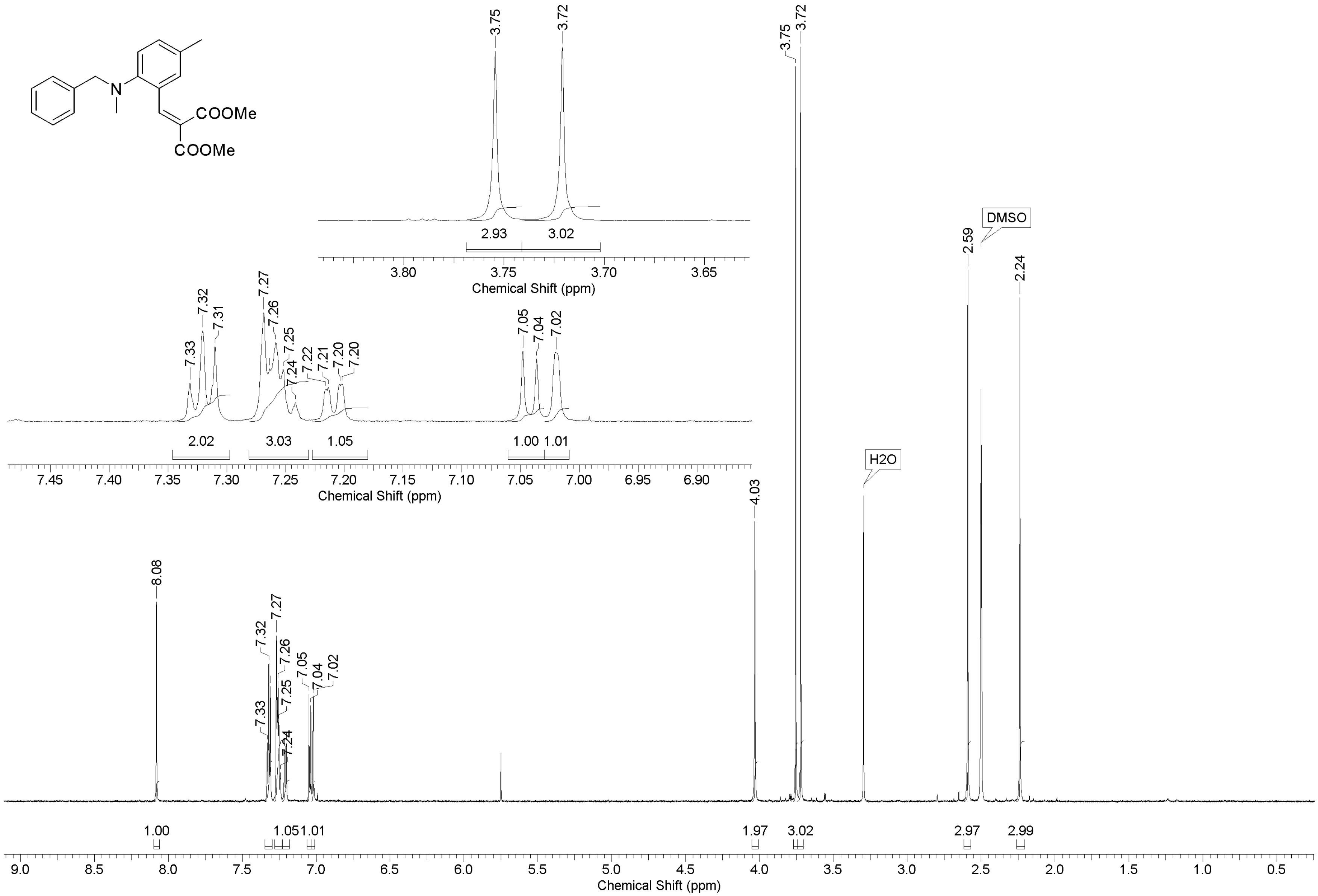
N
COOMe
COOMe
3.75
3.72
2.93
3.02
3.80
3.75
3.70
3.65
Chemical Shift (ppm)
7.33
7.32
7.31
7.27
7.26
7.25
7.24
7.22
7.21
7.20
7.20
7.05
7.04
7.02
2.02
3.03
1.05
1.00
1.01
7.45
7.40
7.35
7.30
7.25
7.20
7.15
7.10
7.05
7.00
6.95
6.90
Chemical Shift (ppm)
8.08
4.03
3.75
3.72
H2O
DMSO
2.59
2.24
1.00
1.05
1.01
1.97
3.02
2.97
2.99
9.0
8.5
8.0
7.5
7.0
6.5
6.0
5.5
5.0
4.5
4.0
3.5
3.0
2.5
2.0
1.5
1.0
0.5
Chemical Shift (ppm)

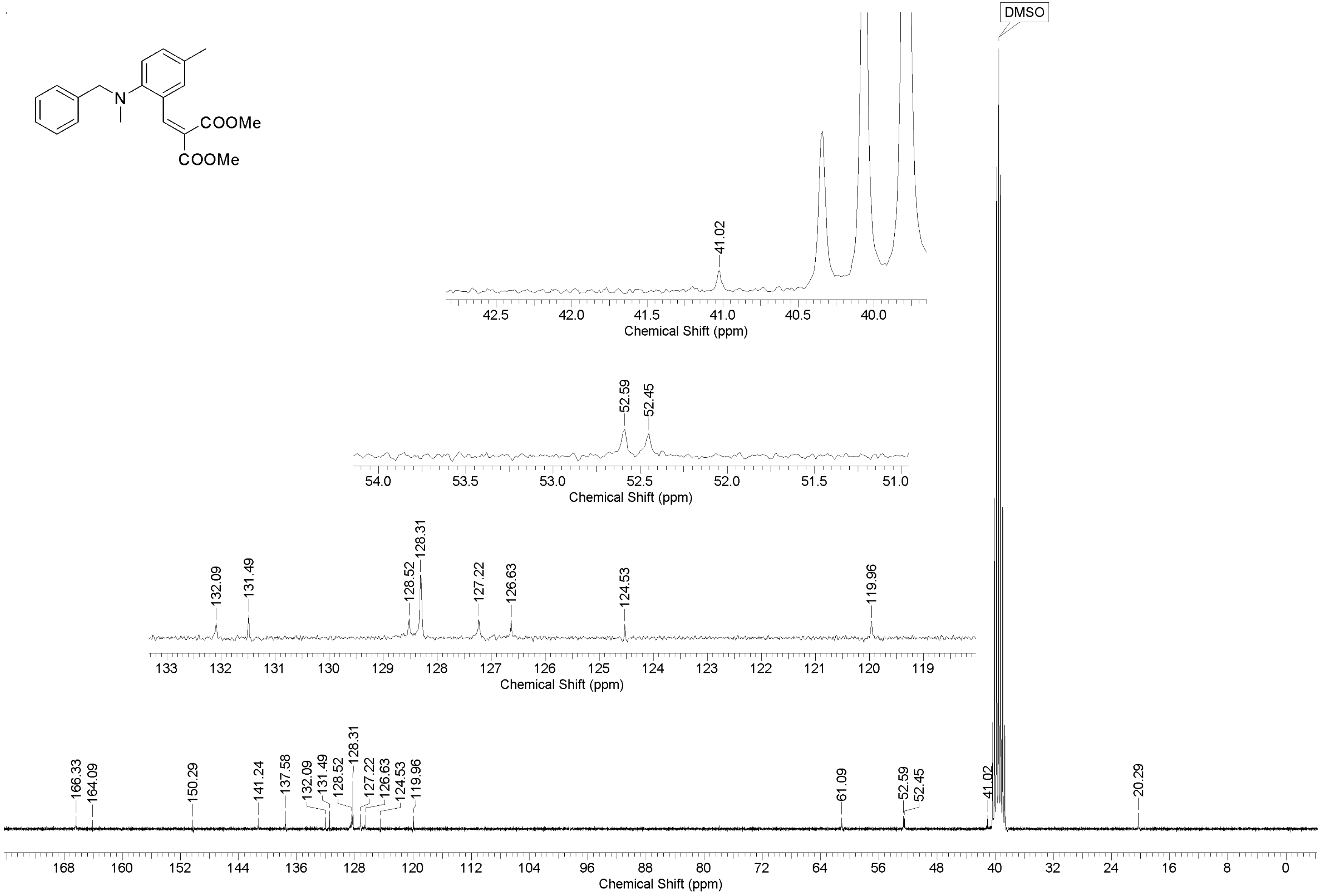

COOMe
COOMe
N
DMSO
41.02
Chemical Shift (ppm)
52.59
52.45
Chemical Shift (ppm)
132.09
131.49
128.52
128.31
127.22
126.63
124.53
119.96
Chemical Shift (ppm)
166.33
164.09
150.29
141.24
137.58
61.09
20.29
Chemical Shift (ppm)

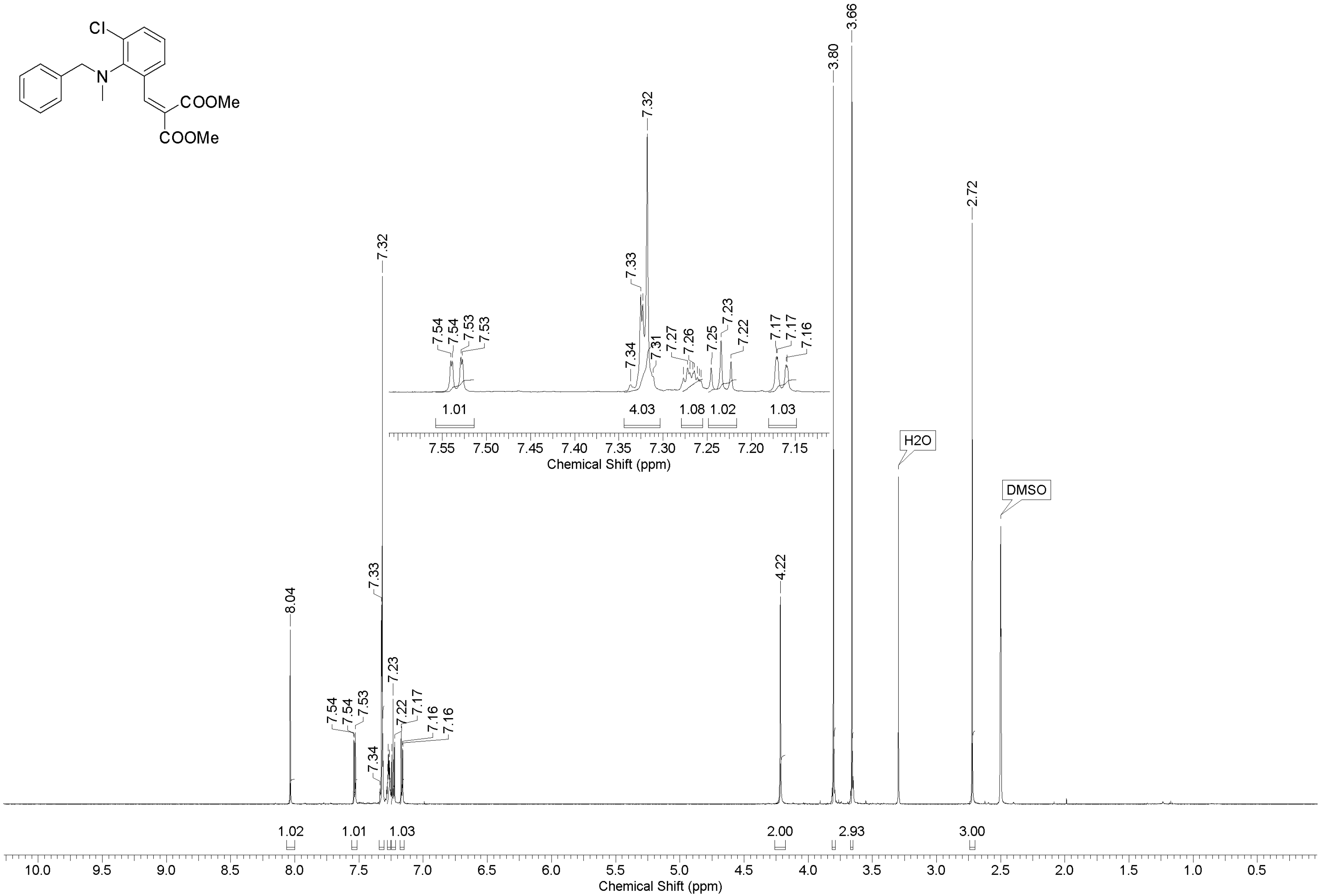
Cl
N
COOMe
COOMe
3.66
3.80
2.72
7.32
7.33
7.54 7.54 7.53 7.53
7.34 7.31 7.27 7.26 7.25 7.23 7.22 7.17 7.17 7.16
1.01 4.03 1.08 1.02 1.03
7.55 7.50 7.45 7.40 7.35 7.30 7.25 7.20 7.15
Chemical Shift (ppm)
H2O
DMSO
4.22
8.04
7.54 7.54 7.53
7.34
7.23 7.22 7.17 7.16 7.16
1.02 1.01 1.03 2.00 2.93 3.00
10.0 9.5 9.0 8.5 8.0 7.5 7.0 6.5 6.0 5.5 5.0 4.5 4.0 3.5 3.0 2.5 2.0 1.5 1.0 0.5
Chemical Shift (ppm)

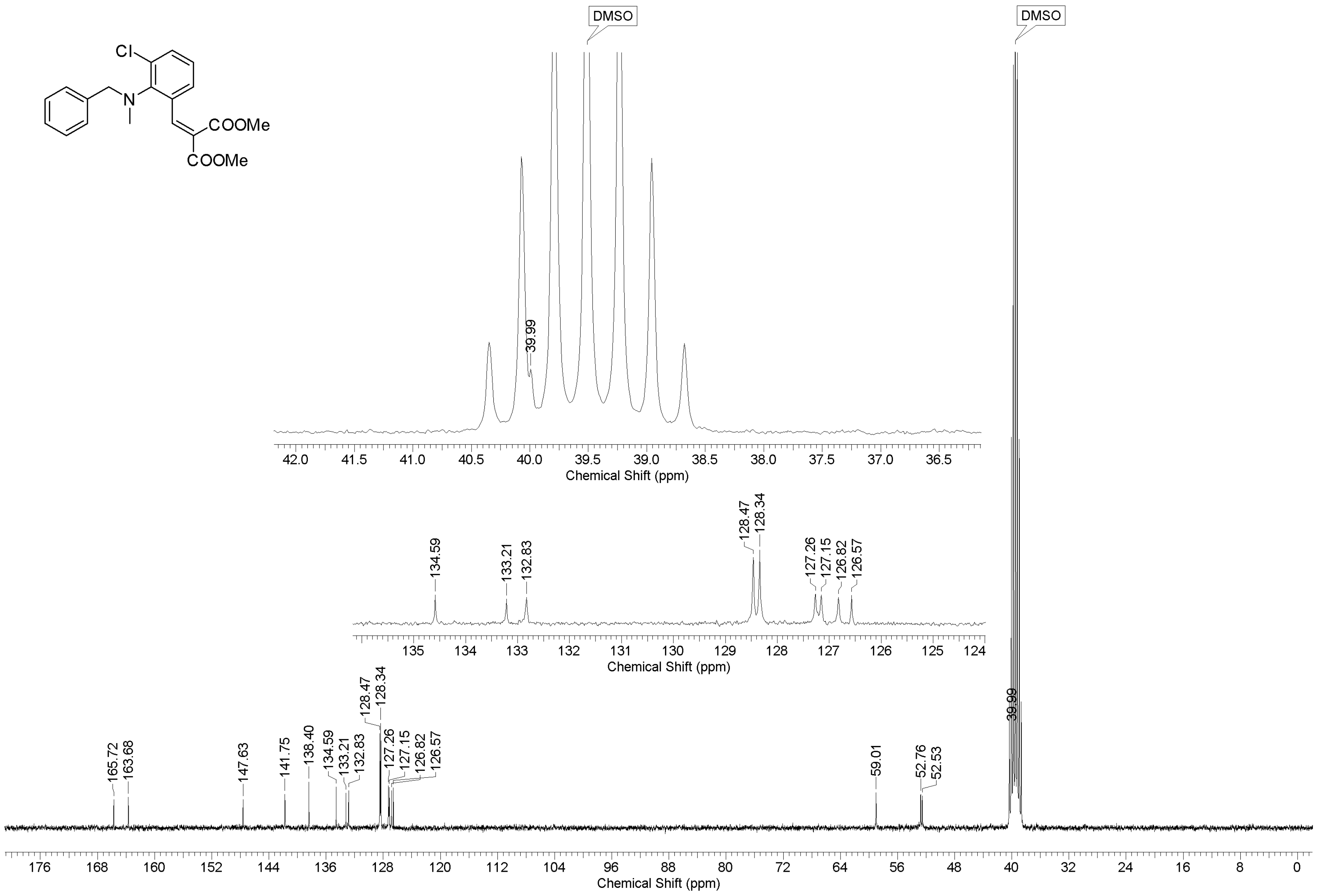

Cl
N
COOMe
COOMe
DMSO
DMSO
39.99
42.0 41.5 41.0 40.5 40.0 39.5 39.0 38.5 38.0 37.5 37.0 36.5
Chemical Shift (ppm)
134.59
133.21
132.83
128.47
128.34
127.26
127.15
126.82
126.57
135 134 133 132 131 130 129 128 127 126 125 124
Chemical Shift (ppm)
165.72
163.68
147.63
141.75
138.40
134.59
133.21
132.83
128.47
128.34
127.26
127.15
126.82
126.57
59.01
52.76
52.53
39.99
176 168 160 152 144 136 128 120 112 104 96 88 80 72 64 56 48 40 32 24 16 8 0
Chemical Shift (ppm)

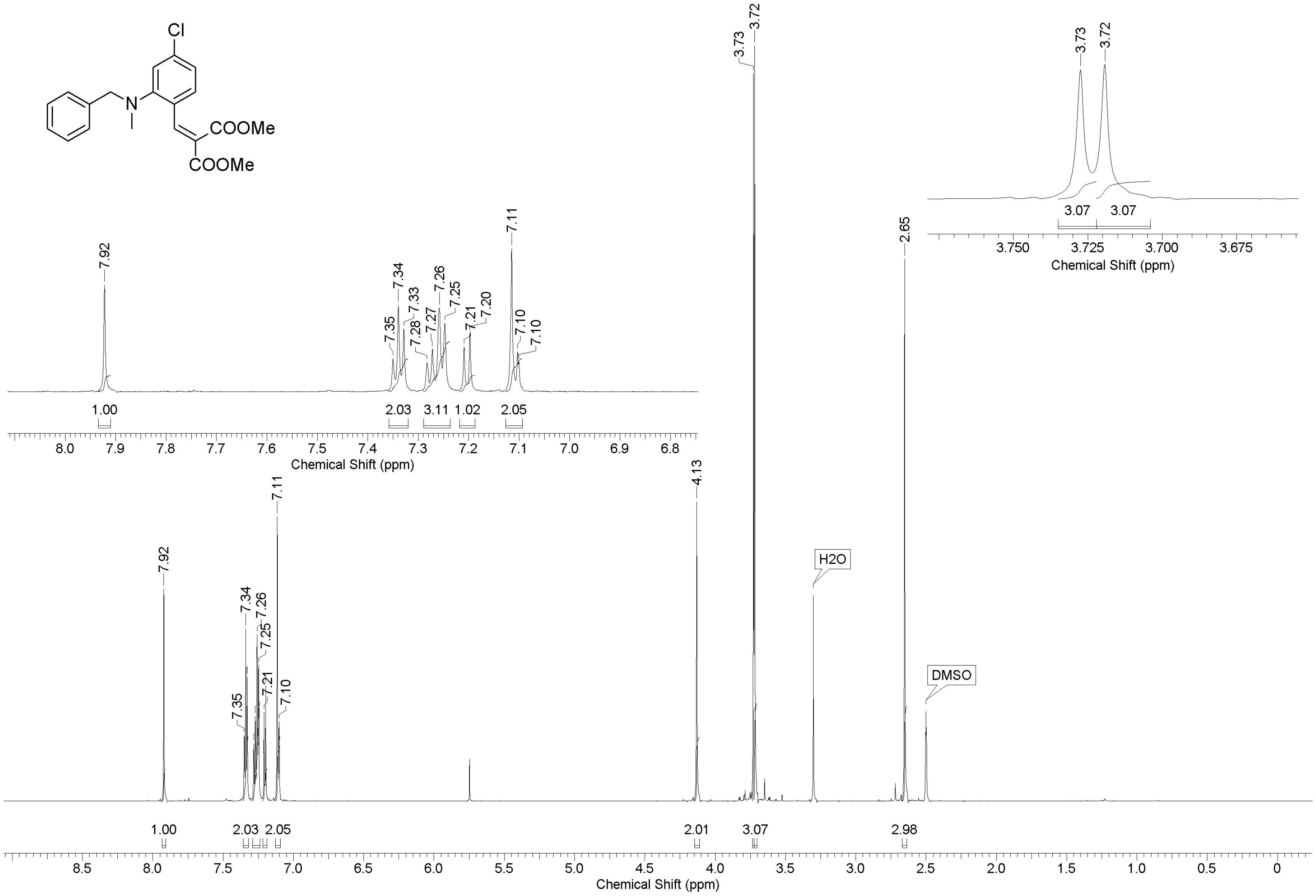
Cl
N
COOMe
COOMe
7.92
7.35
7.34
7.33
7.28
7.27
7.26
7.25
7.21
7.20
7.11
7.10
7.10
1.00
2.03
3.11
1.02
2.05
Chemical Shift (ppm)
3.73
3.72
3.07
3.07
Chemical Shift (ppm)
4.13
2.65
H2O
DMSO
2.01
3.07
2.98
Chemical Shift (ppm)

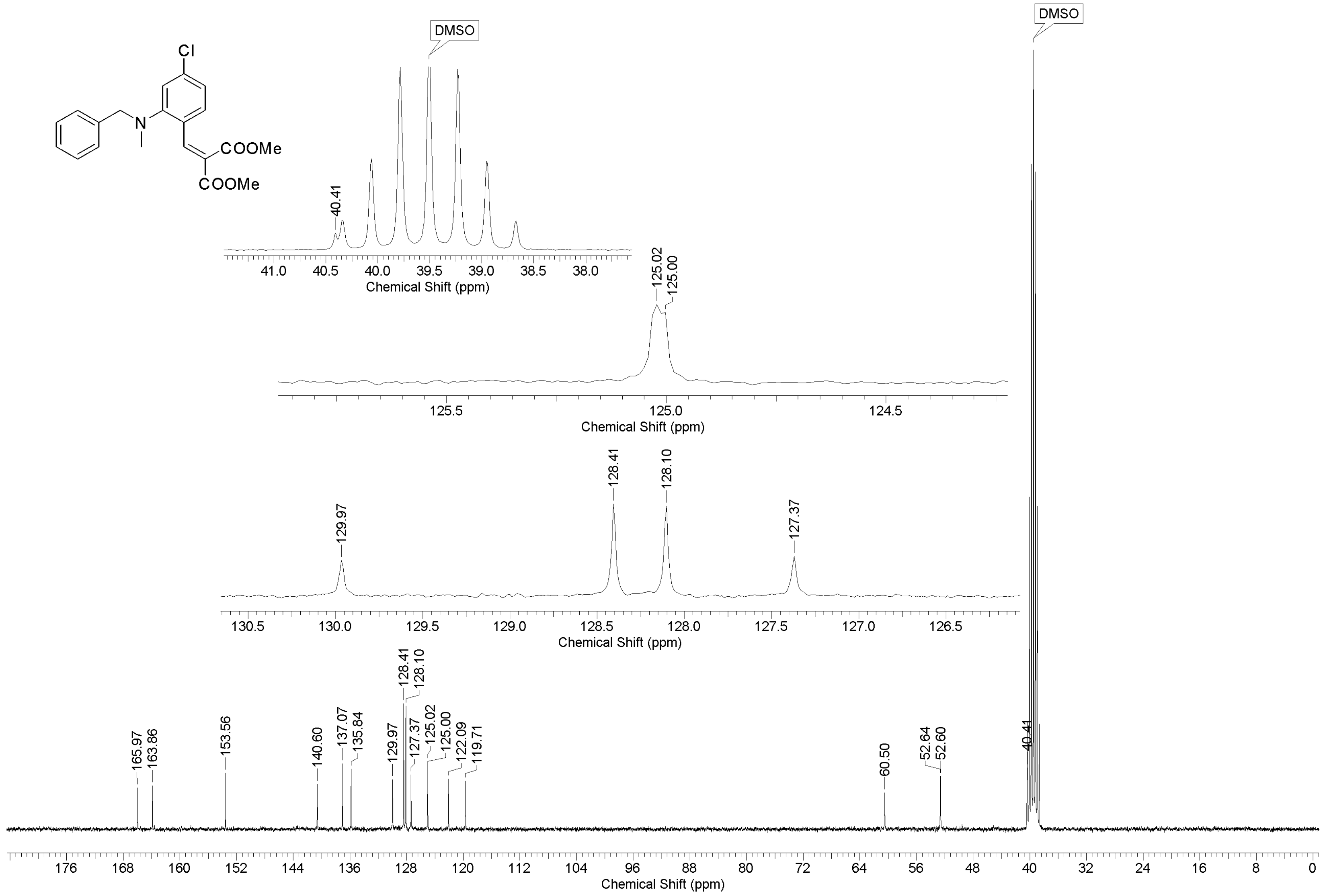
Cl
COOMe
COOMe
DMSO
40.41
41.0
40.5
40.0
39.5
39.0
38.5
38.0
Chemical Shift (ppm)
125.02
125.00
125.5
125.0
124.5
Chemical Shift (ppm)
129.97
128.41
128.10
127.37
130.5
130.0
129.5
129.0
128.5
128.0
127.5
127.0
126.5
Chemical Shift (ppm)
DMSO
165.97
163.86
153.56
140.60
137.07
135.84
129.97
128.41
128.10
127.37
125.02
125.00
122.09
119.71
60.50
52.64
52.60
40.41
176
168
160
152
144
136
128
120
112
104
96
88
80
72
64
56
48
40
32
24
16
8
0
Chemical Shift (ppm)

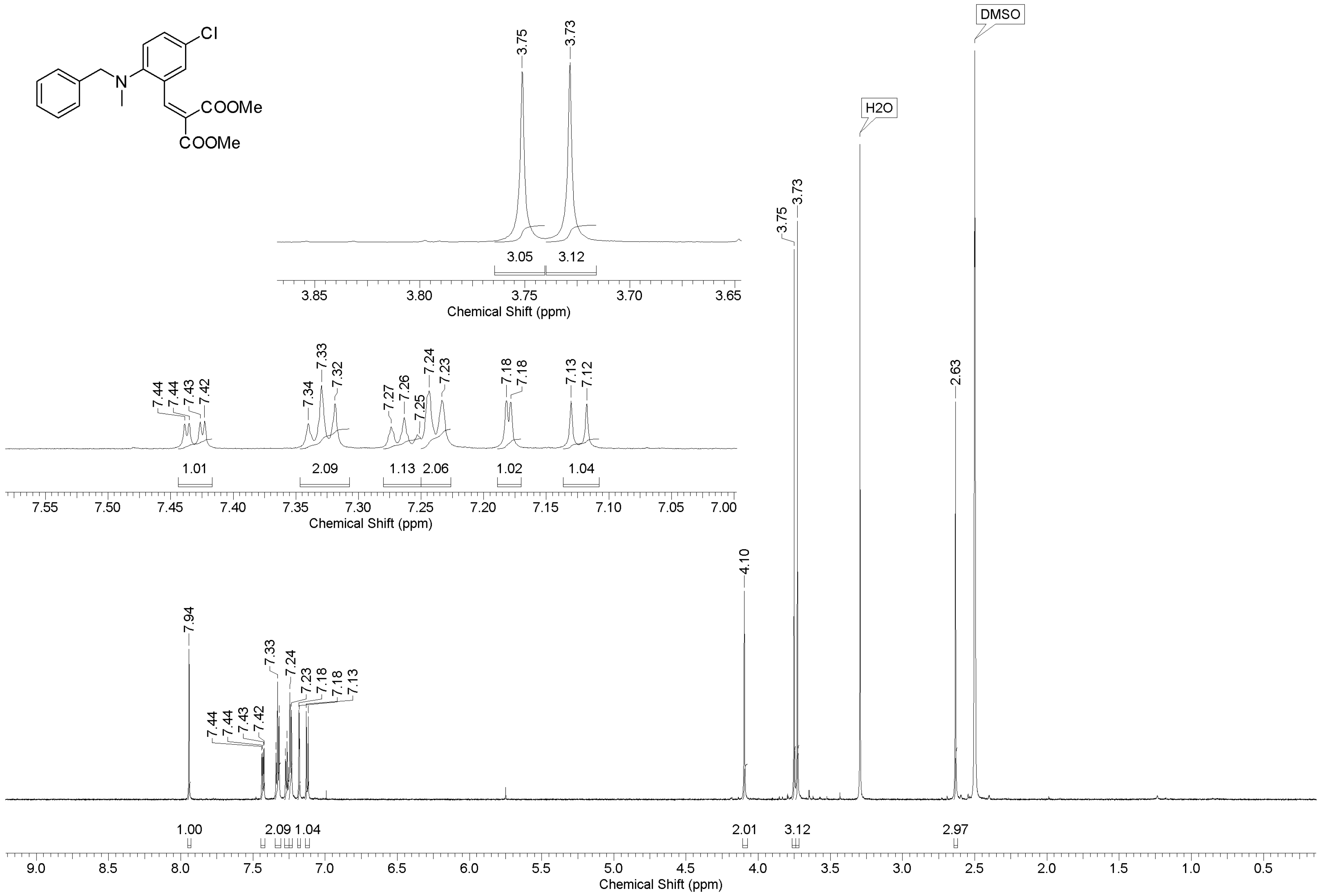
Cl
N
COOMe
COOMe
DMSO
H2O
Chemical Shift (ppm)

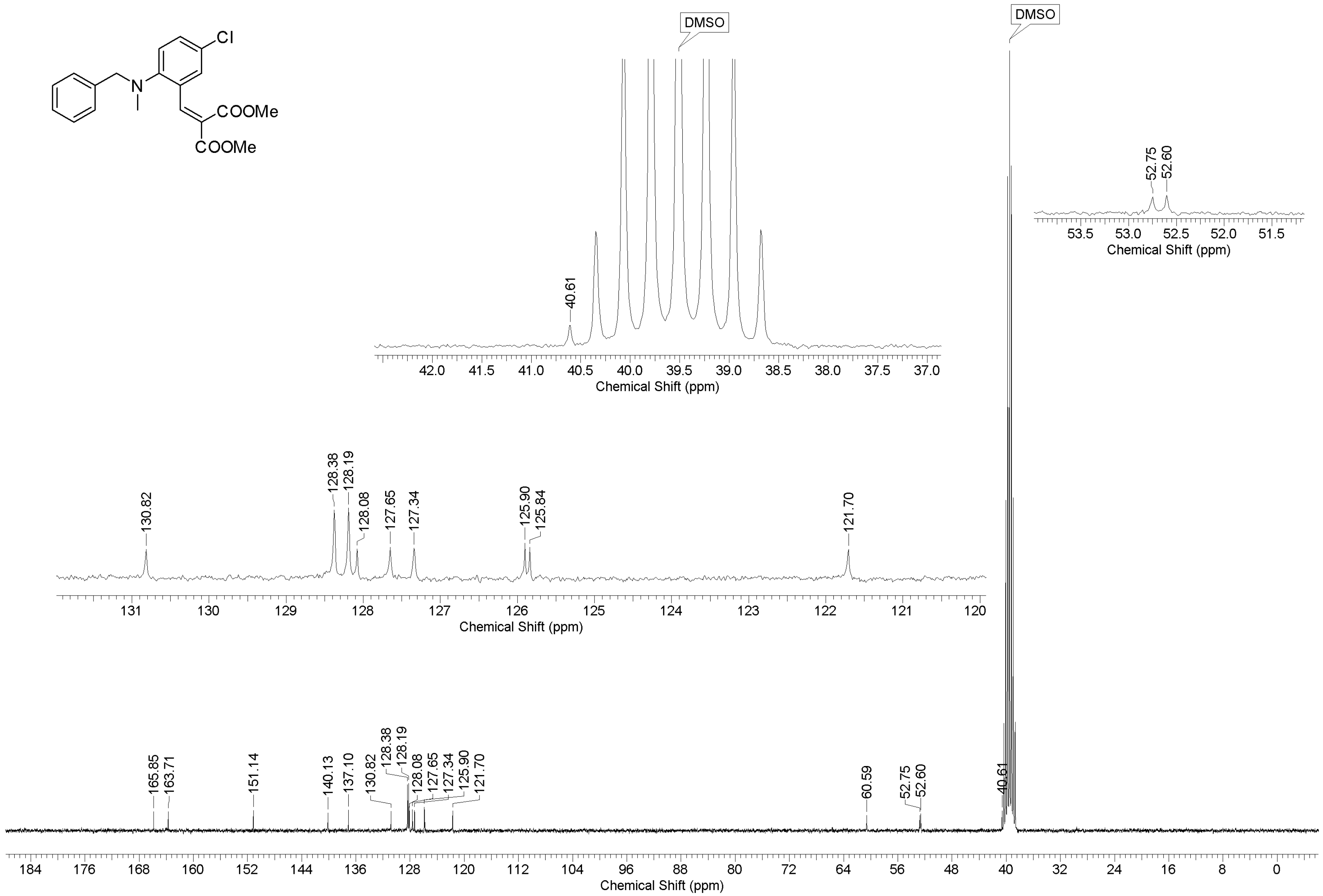
Cl
N
COOMe
COOMe
DMSO
40.61
42.0 41.5 41.0 40.5 40.0 39.5 39.0 38.5 38.0 37.5 37.0
Chemical Shift (ppm)
DMSO
52.75
52.60
53.5 53.0 52.5 52.0 51.5
Chemical Shift (ppm)
130.82
128.38
128.19
128.08
127.65
127.34
125.90
125.84
121.70
131 130 129 128 127 126 125 124 123 122 121 120
Chemical Shift (ppm)
165.85
163.71
151.14
140.13
137.10
130.82
128.38
128.19
128.08
127.65
127.34
125.90
121.70
60.59
52.75
52.60
40.61
184 176 168 160 152 144 136 128 120 112 104 96 88 80 72 64 56 48 40 32 24 16 8 0
Chemical Shift (ppm)

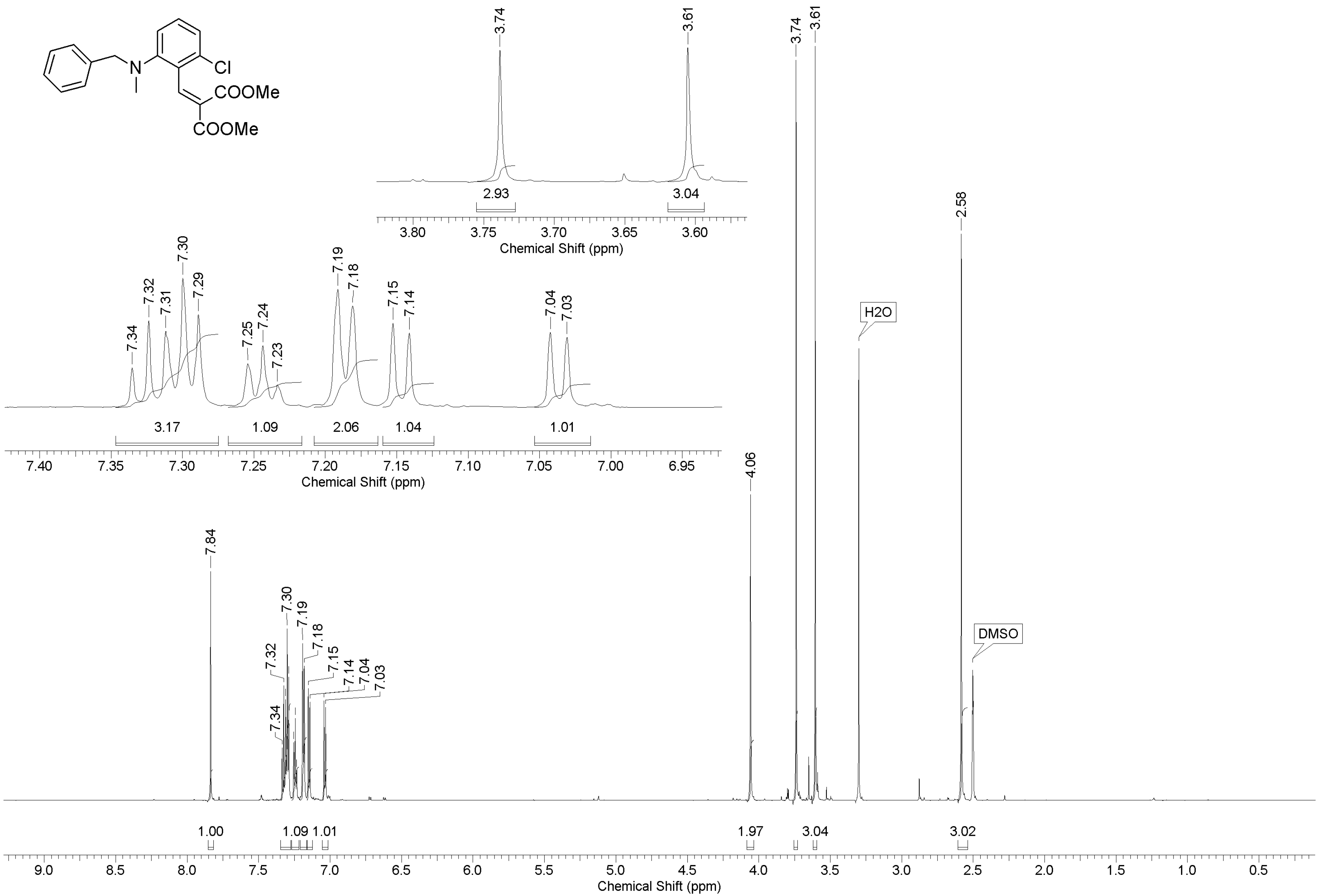

Cl
N
COOMe
COOMe
3.74
3.61
2.93
3.04
3.80
3.75
3.70
3.65
3.60
Chemical Shift (ppm)
7.34
7.32
7.31
7.30
7.29
7.25
7.24
7.23
7.19
7.18
7.15
7.14
7.04
7.03
3.17
1.09
2.06
1.04
1.01
7.40
7.35
7.30
7.25
7.20
7.15
7.10
7.05
7.00
6.95
Chemical Shift (ppm)
H2O
DMSO
7.84
4.06
2.58
1.00
1.09
1.01
1.97
3.04
3.02
9.0
8.5
8.0
7.5
7.0
6.5
6.0
5.5
5.0
4.5
4.0
3.5
3.0
2.5
2.0
1.5
1.0
0.5
Chemical Shift (ppm)

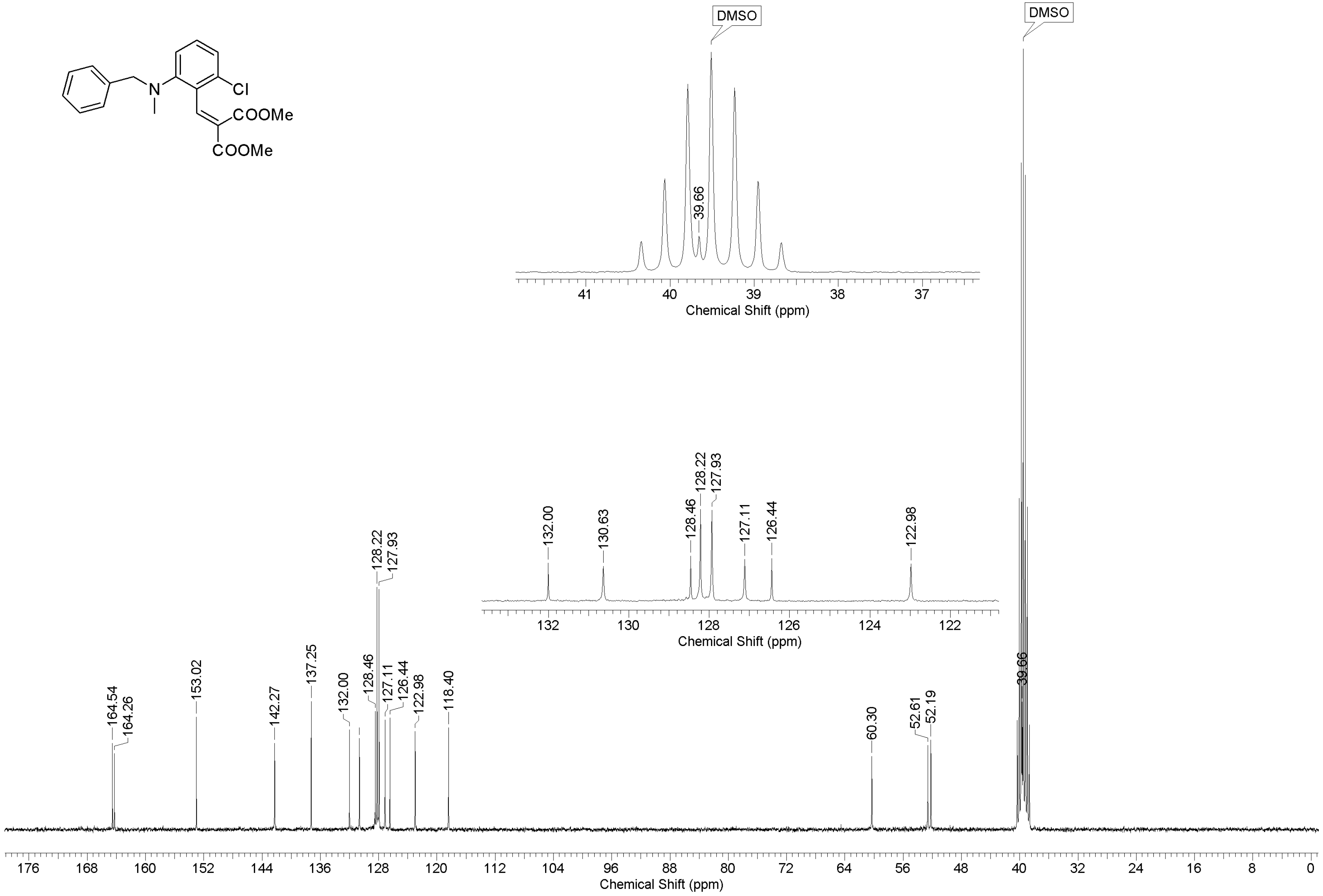
N
Cl
COOMe
COOMe
DMSO
39.66
41
40
39
38
37
Chemical Shift (ppm)
132.00
130.63
128.46
128.22
127.93
127.11
126.44
122.98
132
130
128
126
124
122
Chemical Shift (ppm)
164.54
164.26
153.02
142.27
137.25
132.00
128.46
128.22
127.93
127.11
126.44
122.98
118.40
60.30
52.61
52.19
39.66
176
168
160
152
144
136
128
120
112
104
96
88
80
72
64
56
48
40
32
24
16
8
0
Chemical Shift (ppm)

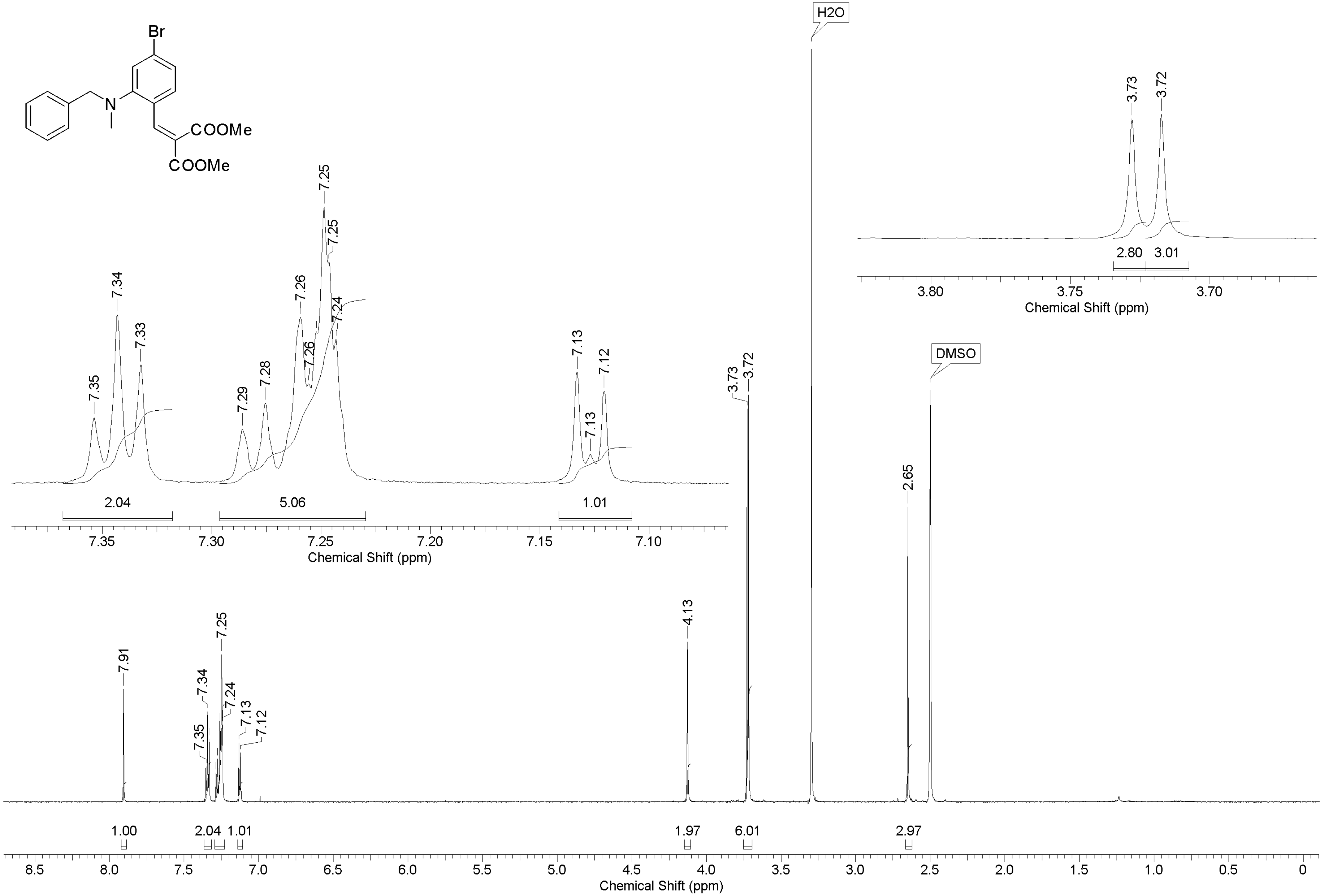

Br
N
COOMe
COOMe
H2O
DMSO
7.91
7.35
7.34
7.25
7.24
7.13
7.12
4.13
3.73
3.72
2.65
1.00
2.04
1.01
1.97
6.01
2.97
Chemical Shift (ppm)
7.33
7.29
7.28
7.26
5.06
2.80
3.01

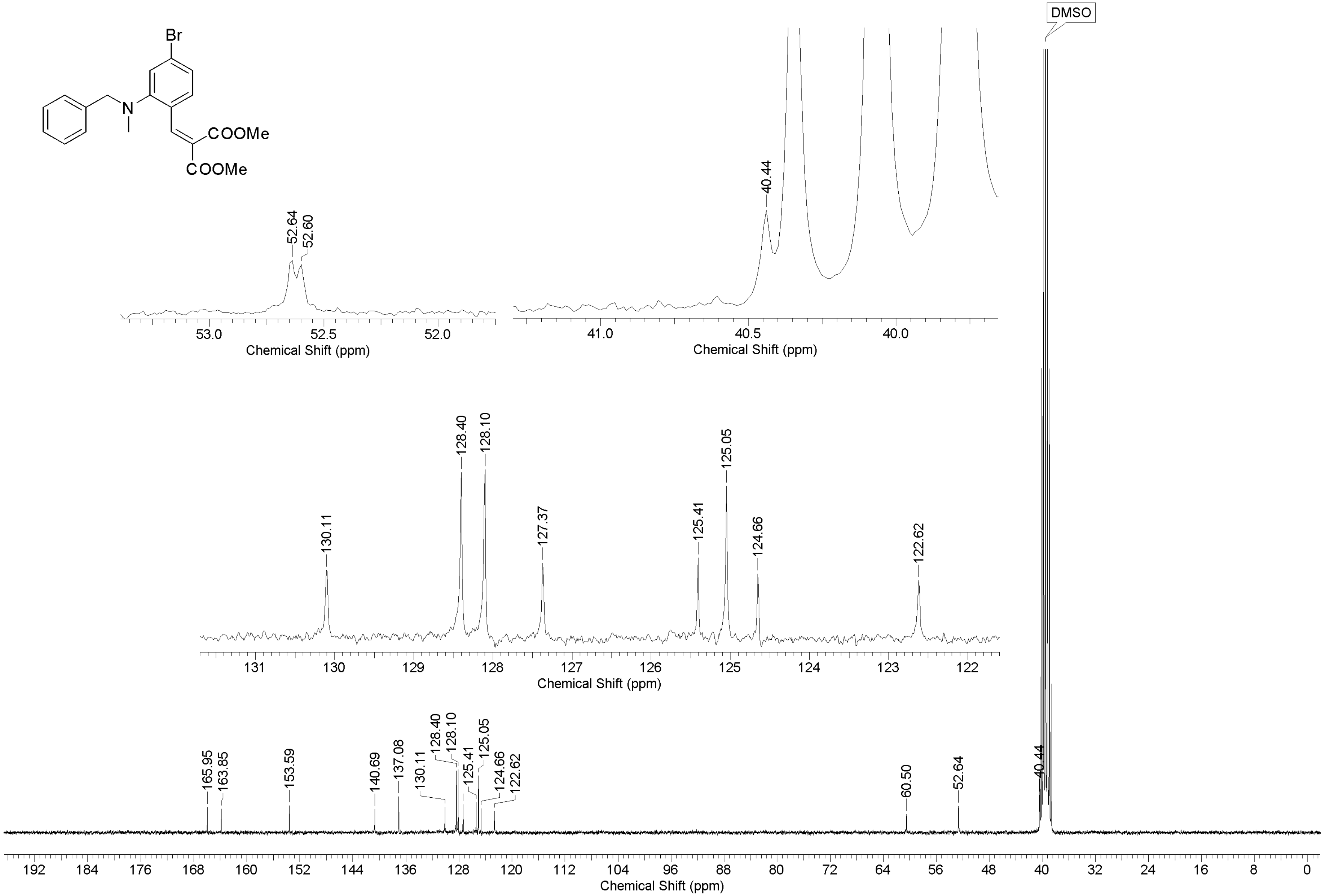
Br
N
COOMe
COOMe
DMSO
52.64
52.60
53.0
52.5
52.0
Chemical Shift (ppm)
40.44
41.0
40.5
40.0
Chemical Shift (ppm)
130.11
128.40
128.10
127.37
125.41
125.05
124.66
122.62
131
130
129
128
127
126
125
124
123
122
Chemical Shift (ppm)
165.95
163.85
153.59
140.69
137.08
130.11
128.40
128.10
125.41
125.05
124.66
122.62
60.50
52.64
40.44
192 184 176 168 160 152 144 136 128 120 112 104 96 88 80 72 64 56 48 40 32 24 16 8 0
Chemical Shift (ppm)

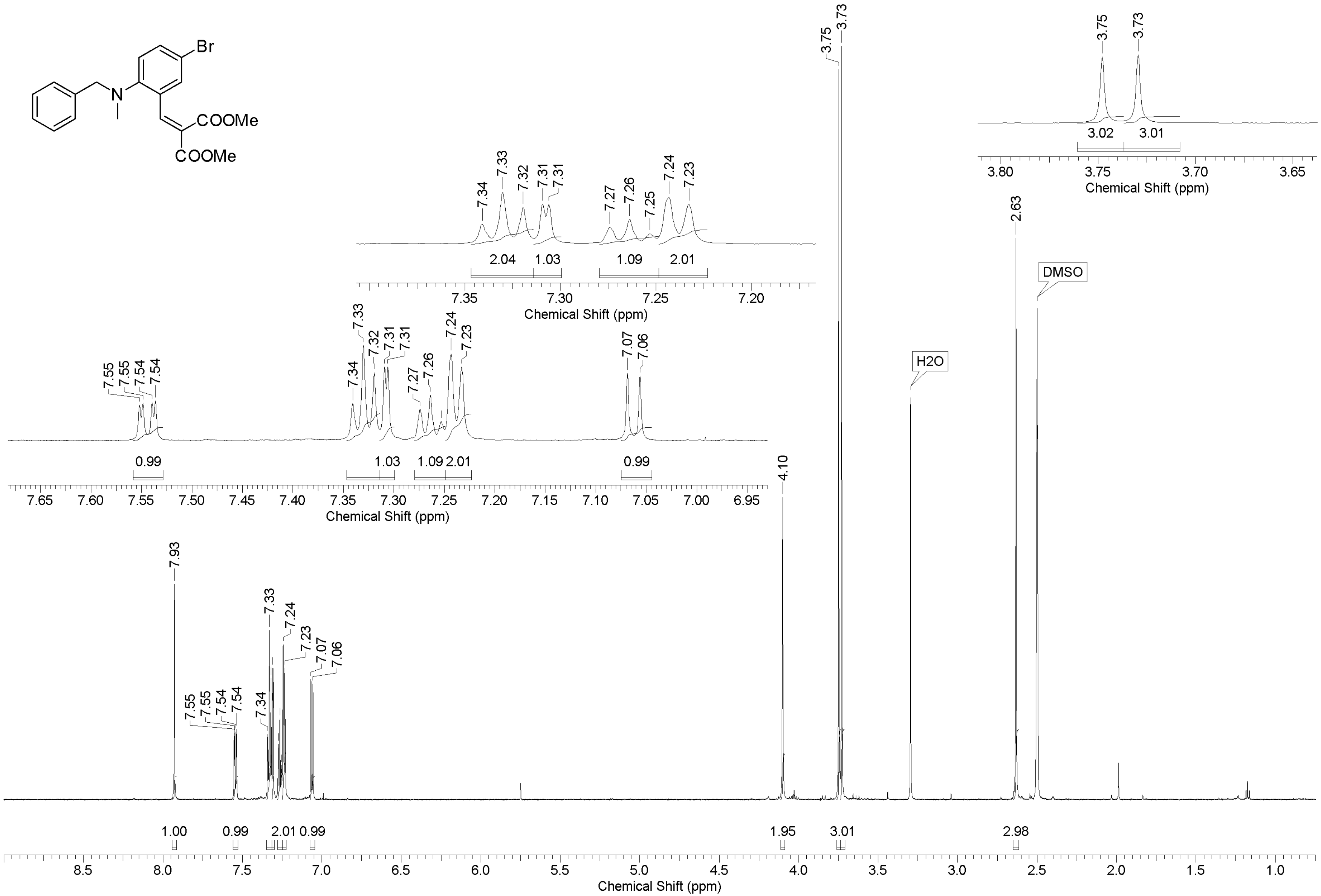
Br
N
COOMe
COOMe
7.34
7.33
7.32
7.31
7.31
7.27
7.26
7.25
7.24
7.23
2.04
1.03
1.09
2.01
Chemical Shift (ppm)
3.75
3.73
3.02
3.01
Chemical Shift (ppm)
7.55
7.55
7.54
7.54
7.07
7.06
0.99
1.03
1.09
2.01
0.99
Chemical Shift (ppm)
7.93
4.10
2.63
DMSO
H2O
1.00
0.99
2.01
0.99
1.95
3.01
2.98
Chemical Shift (ppm)

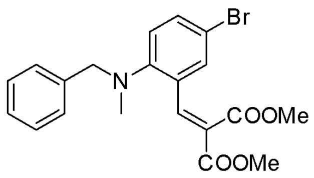
Br
N
COOMe
COOMe

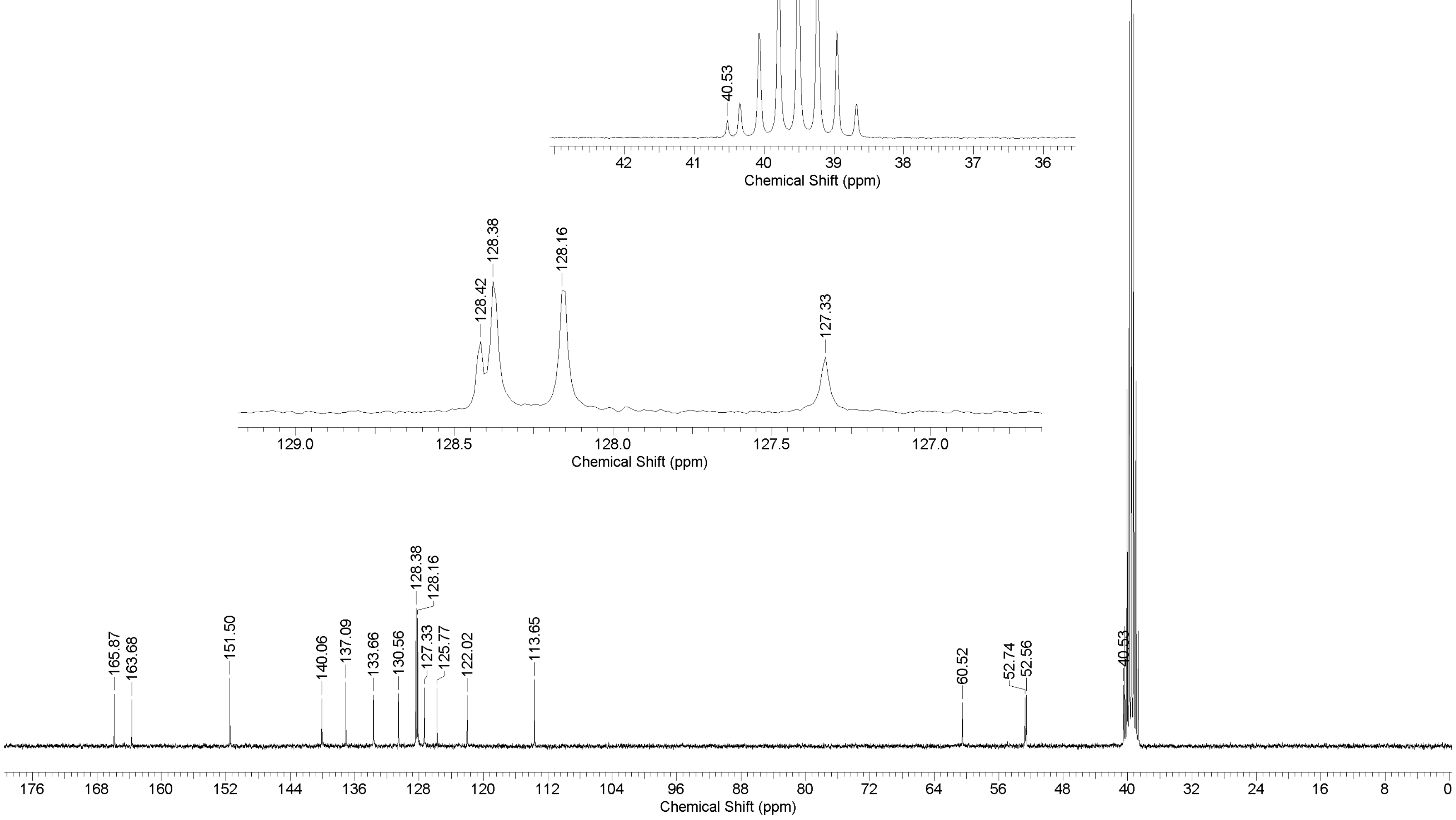
DMSO
DMSO
40.53
42
41
40
39
38
37
36
Chemical Shift (ppm)
128.42
128.38
128.16
127.33
129.0
128.5
128.0
127.5
127.0
Chemical Shift (ppm)
165.87
163.68
151.50
140.06
137.09
133.66
130.56
128.38
128.16
127.33
125.77
122.02
113.65
60.52
52.74
52.56
40.53
176
168
160
152
144
136
128
120
112
104
96
88
80
72
64
56
48
40
32
24
16
8
0
Chemical Shift (ppm)

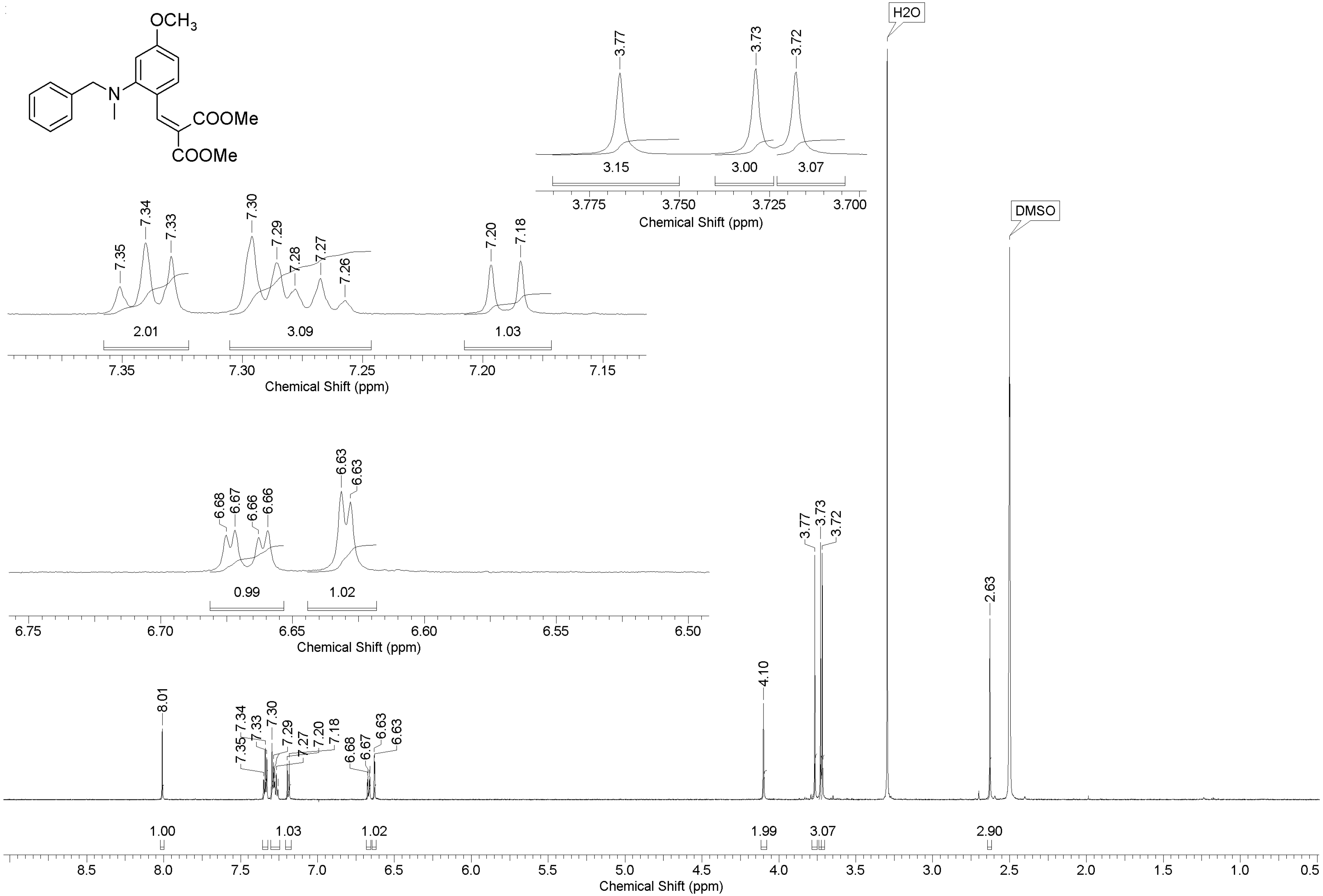
OCH3
N
COOMe
COOMe
H2O
DMSO
3.77
3.73
3.72
3.15
3.00
3.07
3.775
3.750
3.725
3.700
Chemical Shift (ppm)
7.35
7.34
7.33
7.30
7.29
7.28
7.27
7.26
7.20
7.18
2.01
3.09
1.03
7.35
7.30
7.25
7.20
7.15
Chemical Shift (ppm)
6.68
6.67
6.66
6.66
6.63
6.63
0.99
1.02
6.75
6.70
6.65
6.60
6.55
6.50
Chemical Shift (ppm)
8.01
4.10
2.63
1.00
1.03
1.02
1.99
3.07
2.90
8.5
8.0
7.5
7.0
6.5
6.0
5.5
5.0
4.5
4.0
3.5
3.0
2.5
2.0
1.5
1.0
0.5
Chemical Shift (ppm)

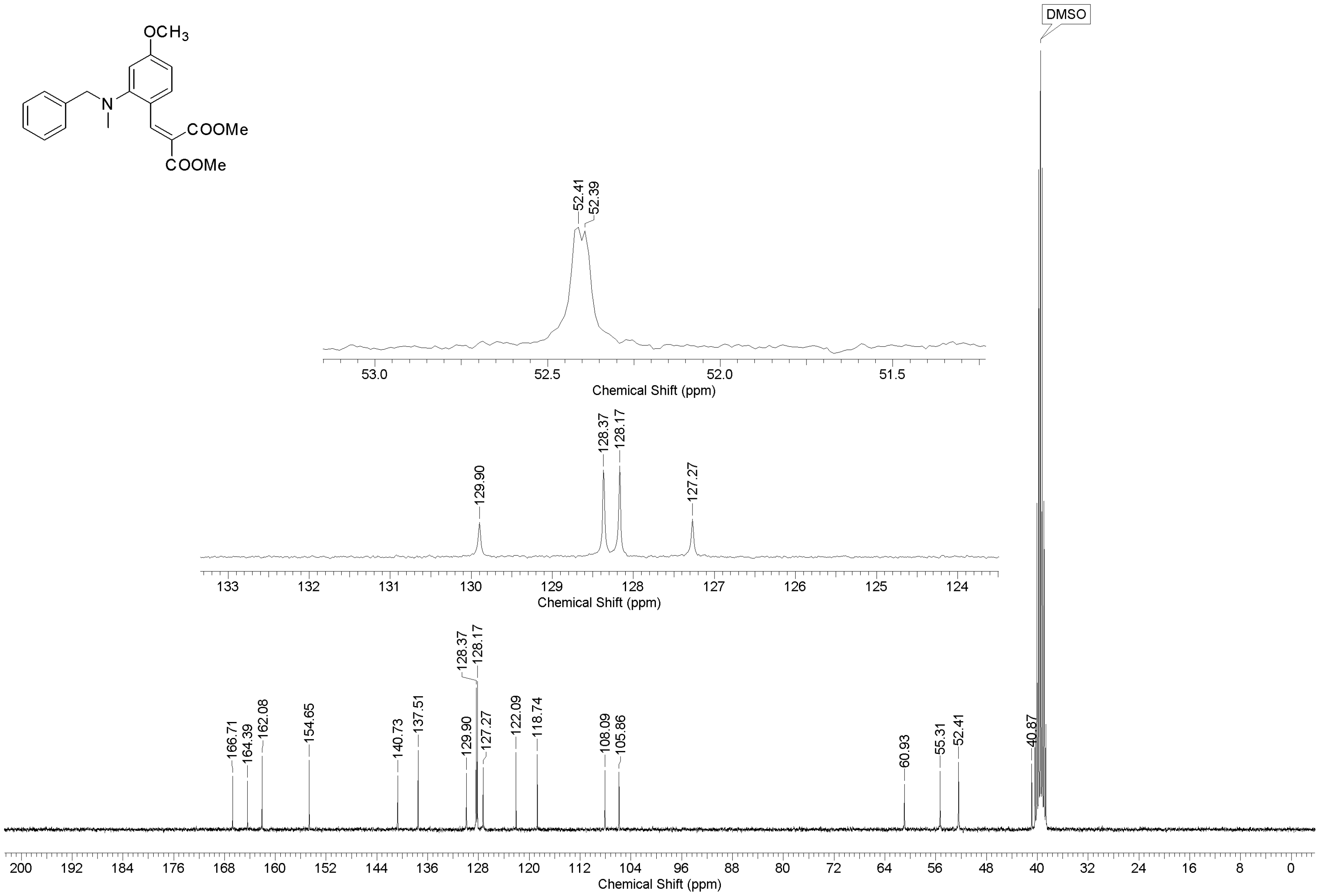
OCH3
N
COOMe
COOMe
DMSO
52.41
52.39
53.0 52.5 52.0 51.5
Chemical Shift (ppm)
129.90
128.37
128.17
127.27
133 132 131 130 129 128 127 126 125 124
Chemical Shift (ppm)
166.71
164.39
162.08
154.65
140.73
137.51
129.90
128.37
128.17
127.27
122.09
118.74
108.09
105.86
60.93
55.31
52.41
40.87
200 192 184 176 168 160 152 144 136 128 120 112 104 96 88 80 72 64 56 48 40 32 24 16 8 0
Chemical Shift (ppm)

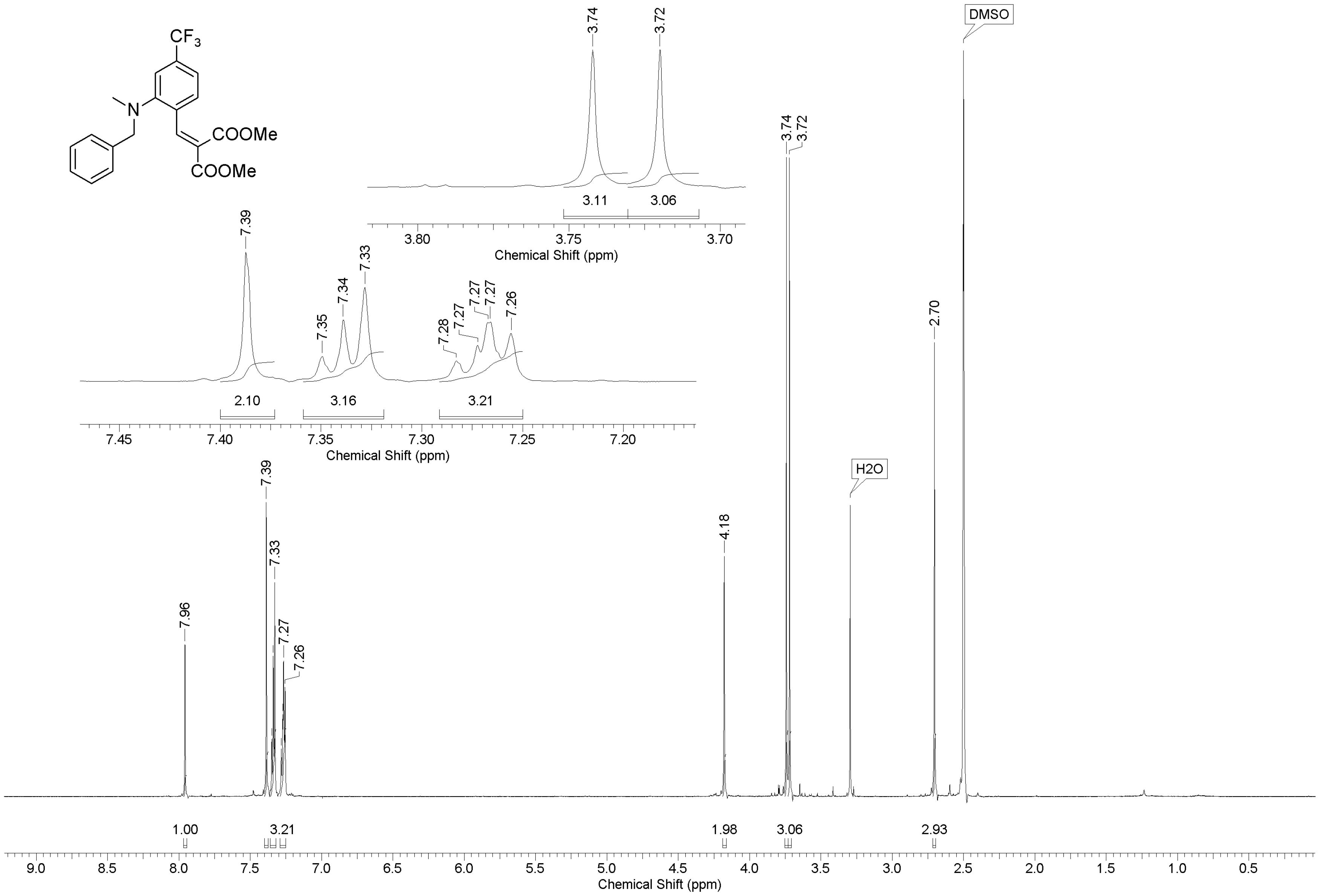
CF3
N
COOMe
COOMe
DMSO
H2O
3.74
3.72
3.11
3.06
3.80
3.75
3.70
Chemical Shift (ppm)
7.39
7.35
7.34
7.33
7.28
7.27
7.27
7.27
7.26
2.10
3.16
3.21
7.45
7.40
7.35
7.30
7.25
7.20
Chemical Shift (ppm)
7.96
7.39
7.33
7.27
7.26
4.18
3.74
3.72
2.70
1.00
3.21
1.98
3.06
2.93
9.0
8.5
8.0
7.5
7.0
6.5
6.0
5.5
5.0
4.5
4.0
3.5
3.0
2.5
2.0
1.5
1.0
0.5
Chemical Shift (ppm)

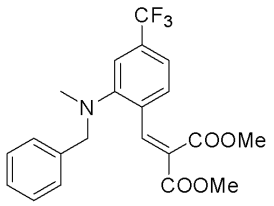
CF3
N
COOMe
COOMe

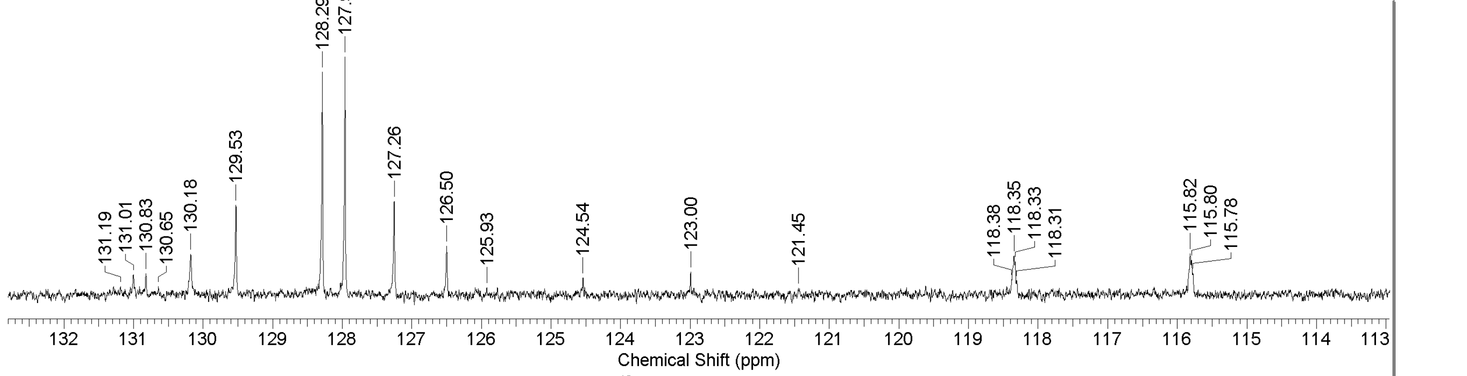
DMSO
131.19
131.01
130.83
130.65
130.18
129.53
128.29
127.96
127.26
126.50
125.93
124.54
123.00
121.45
118.38
118.35
118.33
118.31
115.82
115.80
115.78
Chemical Shift (ppm)

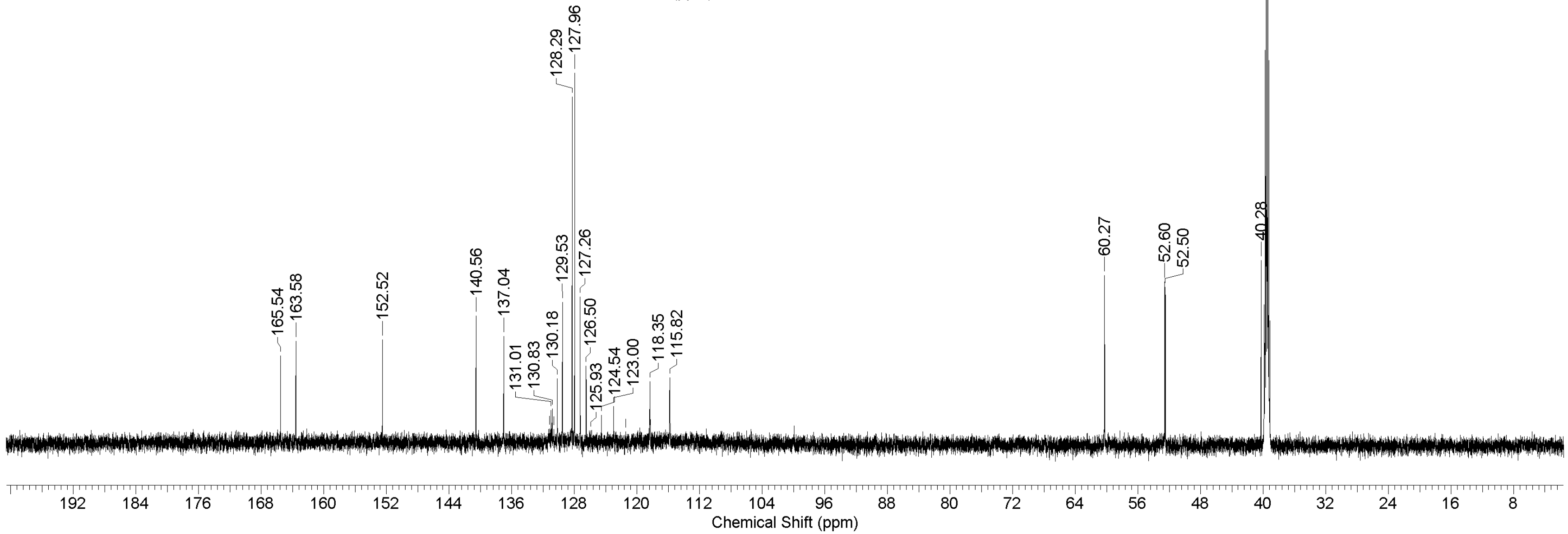
165.54
163.58
152.52
140.56
137.04
131.01
130.83
130.18
129.53
128.29
127.96
127.26
126.50
125.93
124.54
123.00
118.35
115.82
60.27
52.60
52.50
40.28
Chemical Shift (ppm)

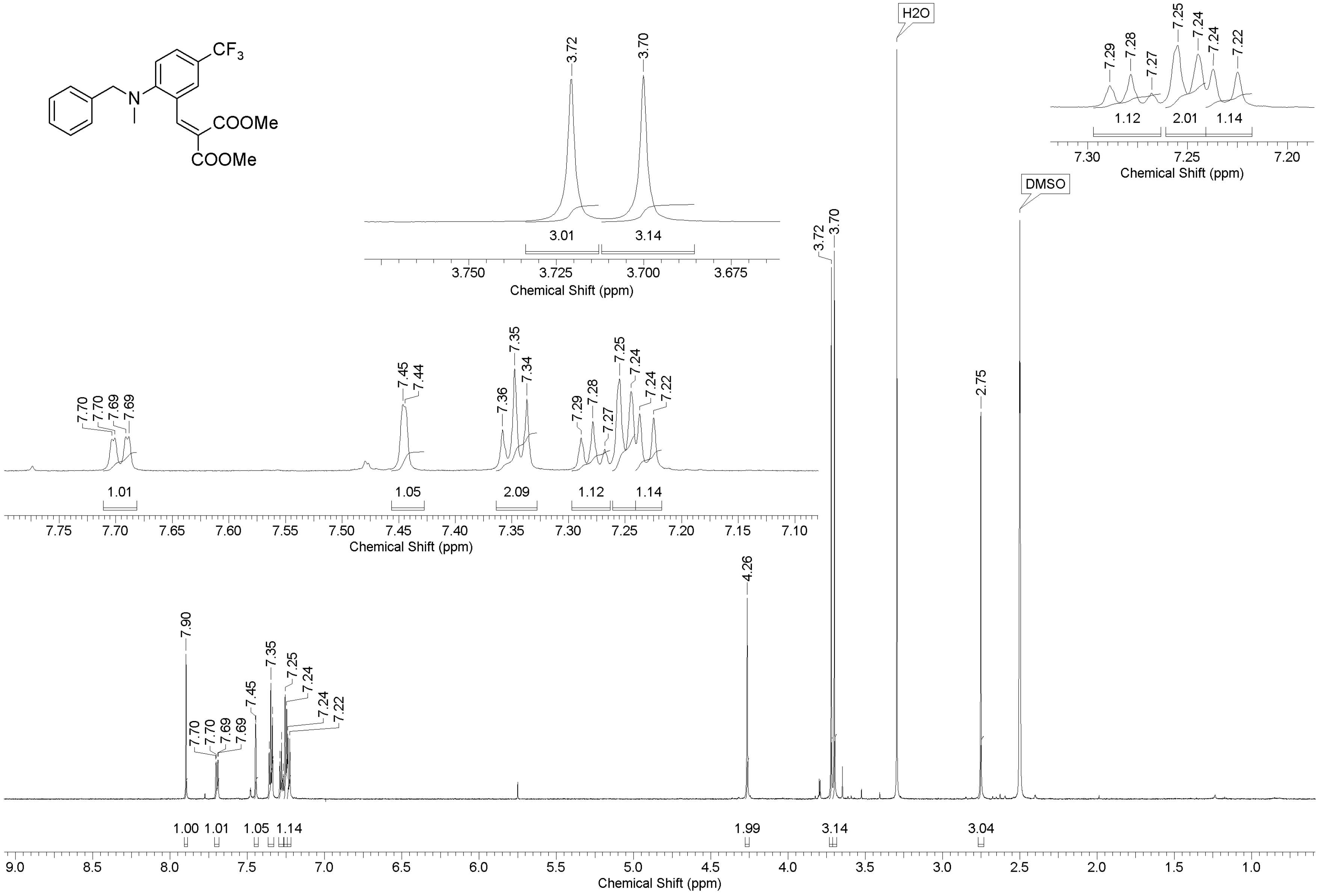
CF3
N
COOMe
COOMe
H2O
DMSO
Chemical Shift (ppm)

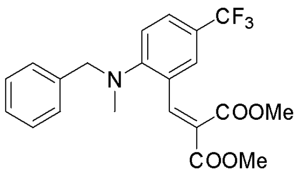
CF3
N
COOMe
COOMe

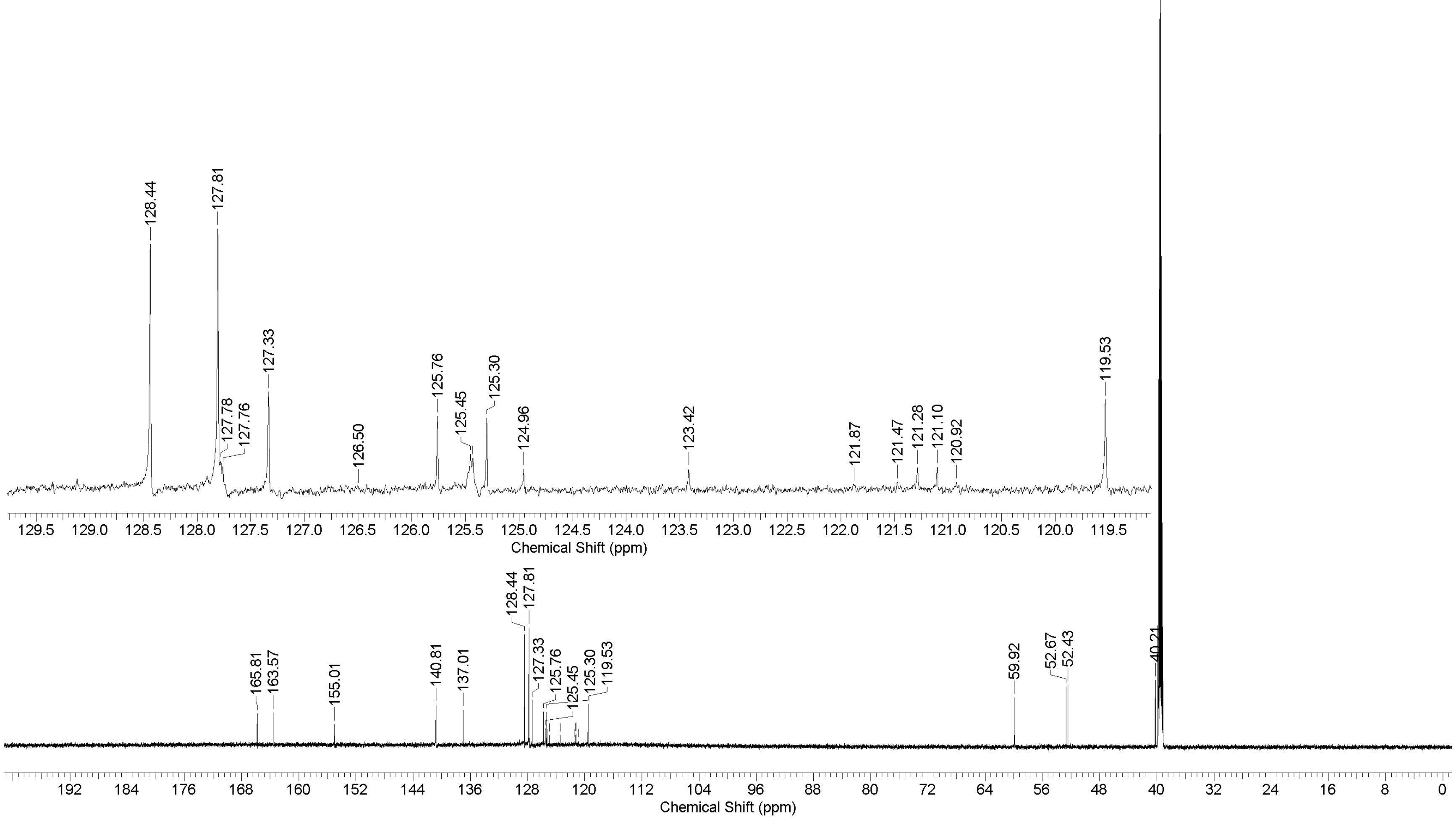
DMSO
Chemical Shift (ppm)
Chemical Shift (ppm)

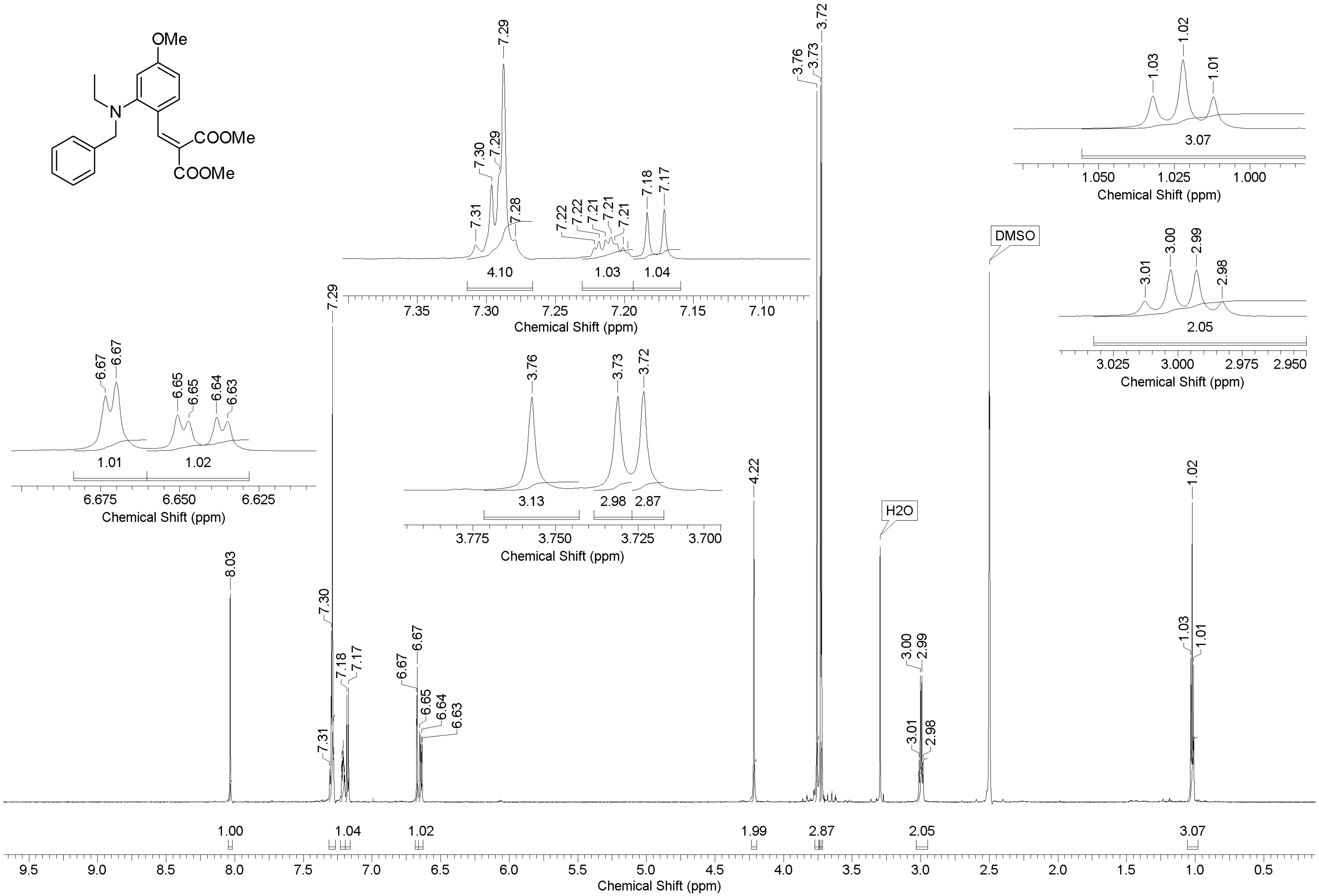
OMe
N
COOMe
COOMe
DMSO
H2O
8.03
7.31
7.30
7.29
7.18
7.17
6.67
6.67
6.65
6.64
6.63
4.22
3.76
3.73
3.72
3.01
3.00
2.99
2.98
1.03
1.02
1.01
1.00
1.04
1.02
1.99
2.87
2.05
3.07
4.10
1.03
1.01
3.13
2.98
Chemical Shift (ppm)

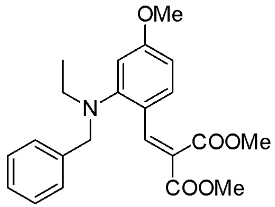
OMe
N
COOMe
COOMe

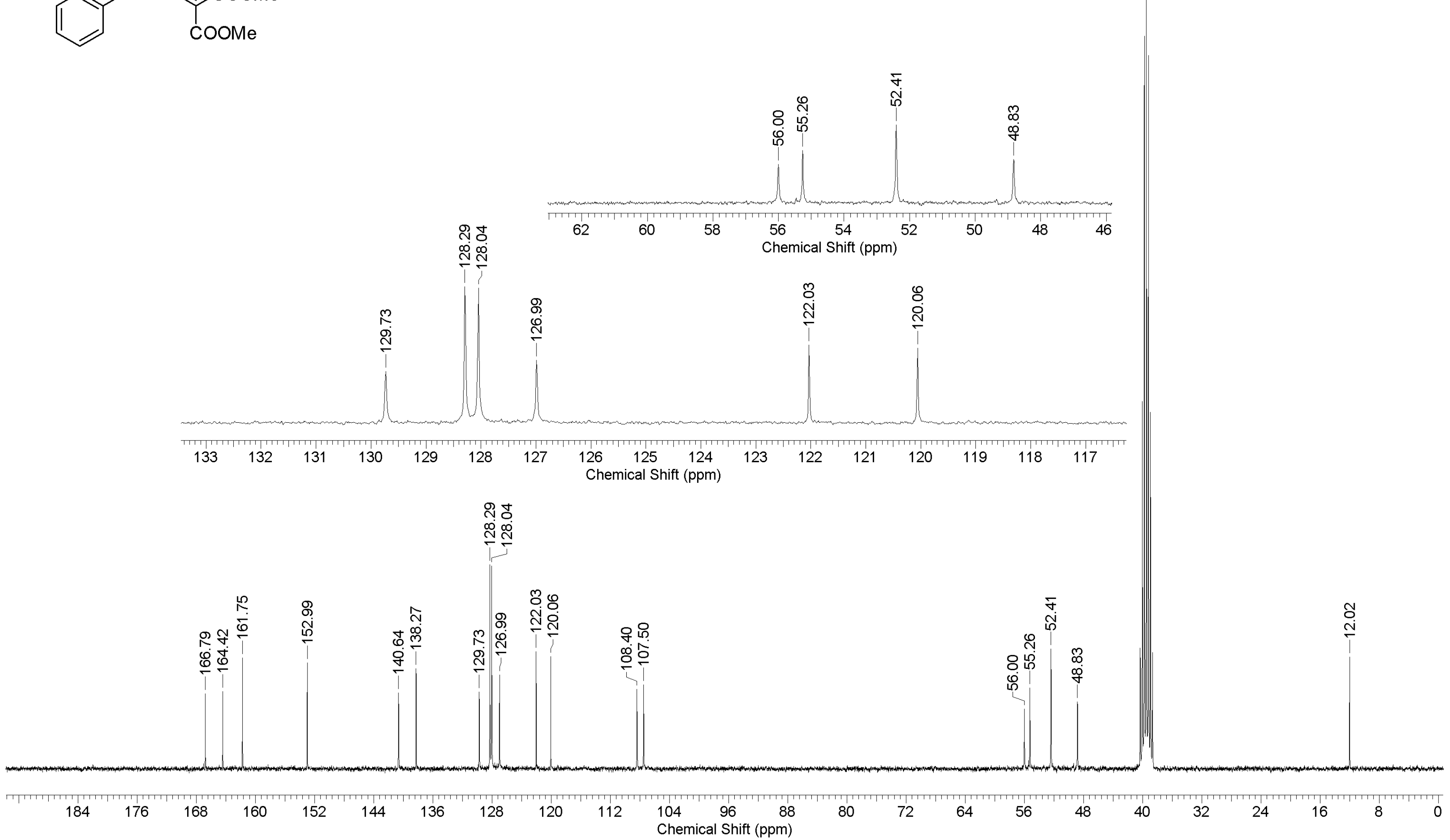
DMSO
56.00
55.26
52.41
48.83
62
60
58
56
54
52
50
48
46
Chemical Shift (ppm)
129.73
128.29
128.04
126.99
122.03
120.06
133
132
131
130
129
128
127
126
125
124
123
122
121
120
119
118
117
Chemical Shift (ppm)
166.79
164.42
161.75
152.99
140.64
138.27
129.73
128.29
128.04
126.99
122.03
120.06
108.40
107.50
56.00
55.26
52.41
48.83
12.02
184
176
168
160
152
144
136
128
120
112
104
96
88
80
72
64
56
48
40
32
24
16
8
0
Chemical Shift (ppm)

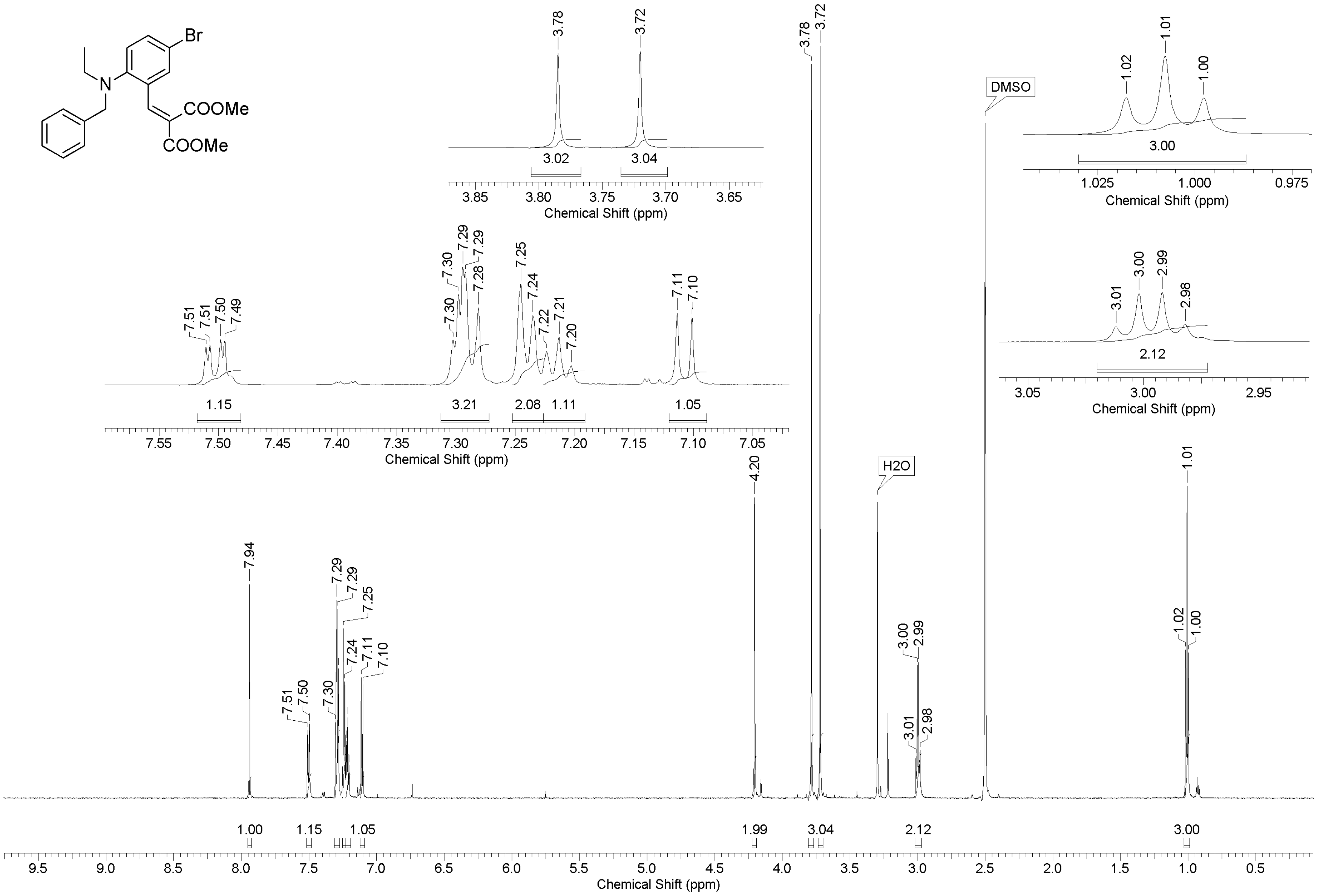
Br
N
COOMe
COOMe
DMSO
H2O
Chemical Shift (ppm)
1.00
1.15
1.05
1.99
3.04
2.12
3.00
3.02
3.21
2.08
1.11

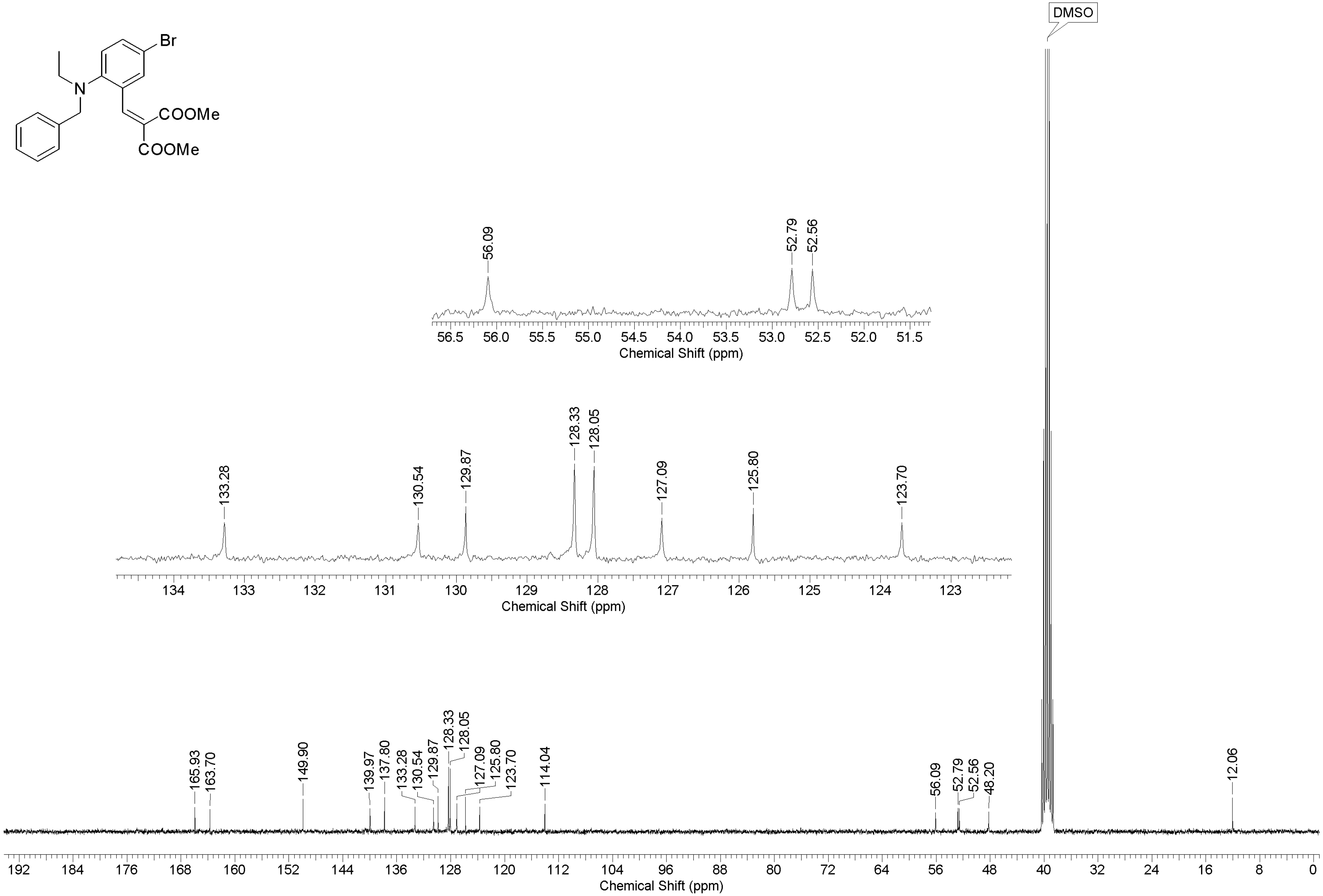
Br
N
COOMe
COOMe
DMSO
56.09
52.79
52.56
Chemical Shift (ppm)
133.28
130.54
129.87
128.33
128.05
127.09
125.80
123.70
Chemical Shift (ppm)
165.93
163.70
149.90
139.97
137.80
133.28
130.54
129.87
128.33
128.05
127.09
125.80
123.70
114.04
56.09
52.79
52.56
48.20
12.06
Chemical Shift (ppm)

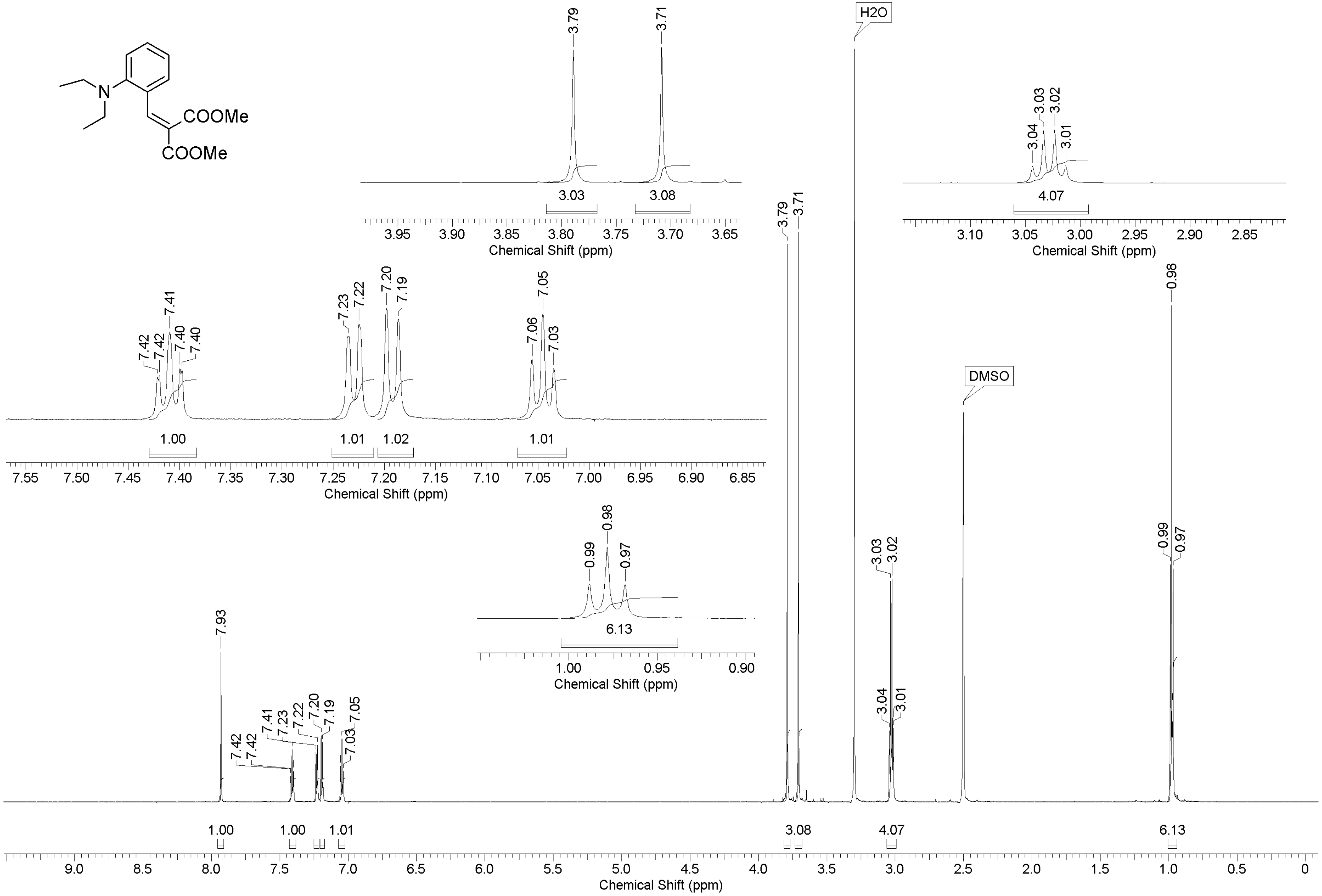

COOMe
COOMe
N
H2O
DMSO
Chemical Shift (ppm)

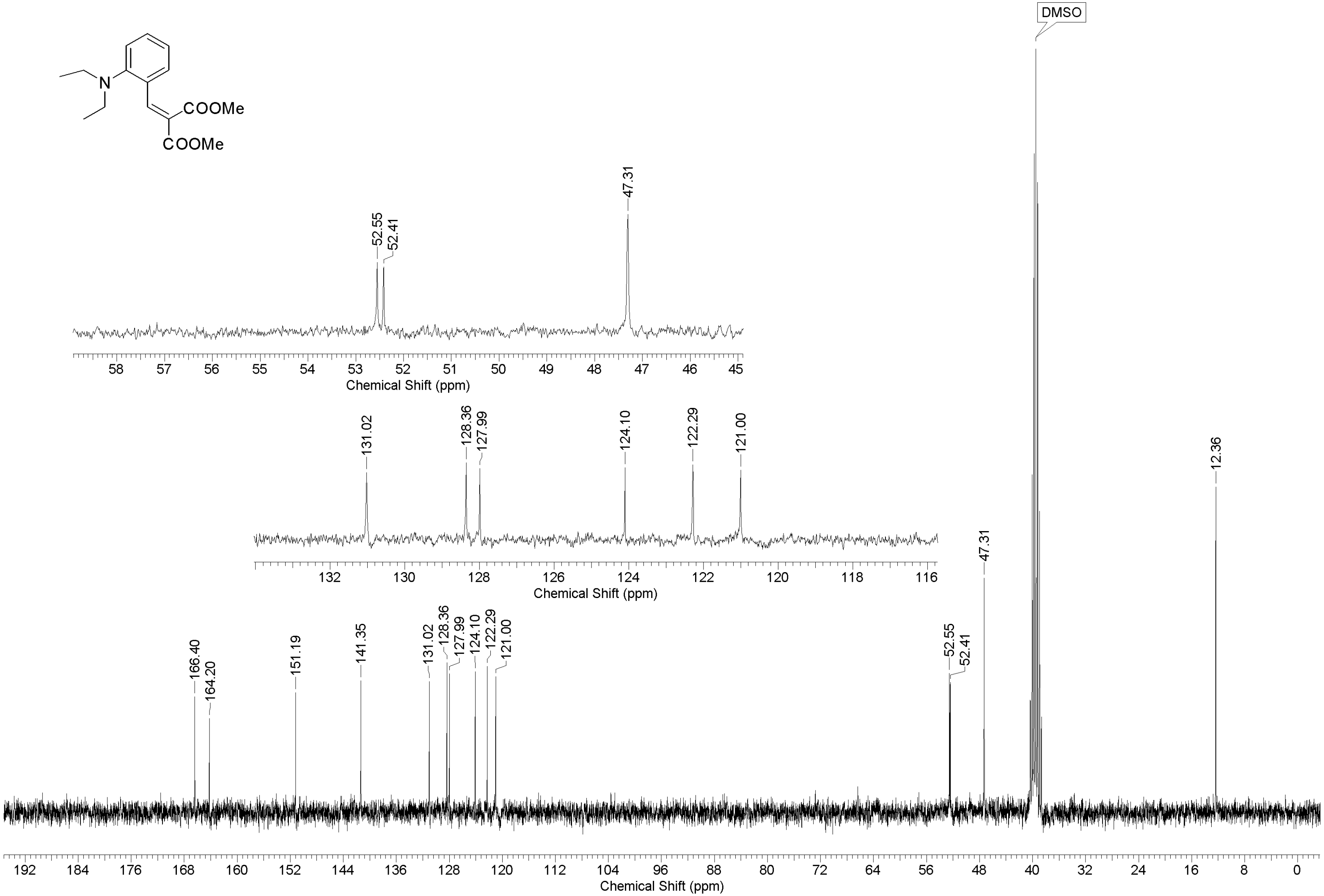
N
COOMe
COOMe
DMSO
52.55
52.41
47.31
Chemical Shift (ppm)
131.02
128.36
127.99
124.10
122.29
121.00
Chemical Shift (ppm)
166.40
164.20
151.19
141.35
131.02
128.36
127.99
124.10
122.29
121.00
52.55
52.41
47.31
12.36
Chemical Shift (ppm)

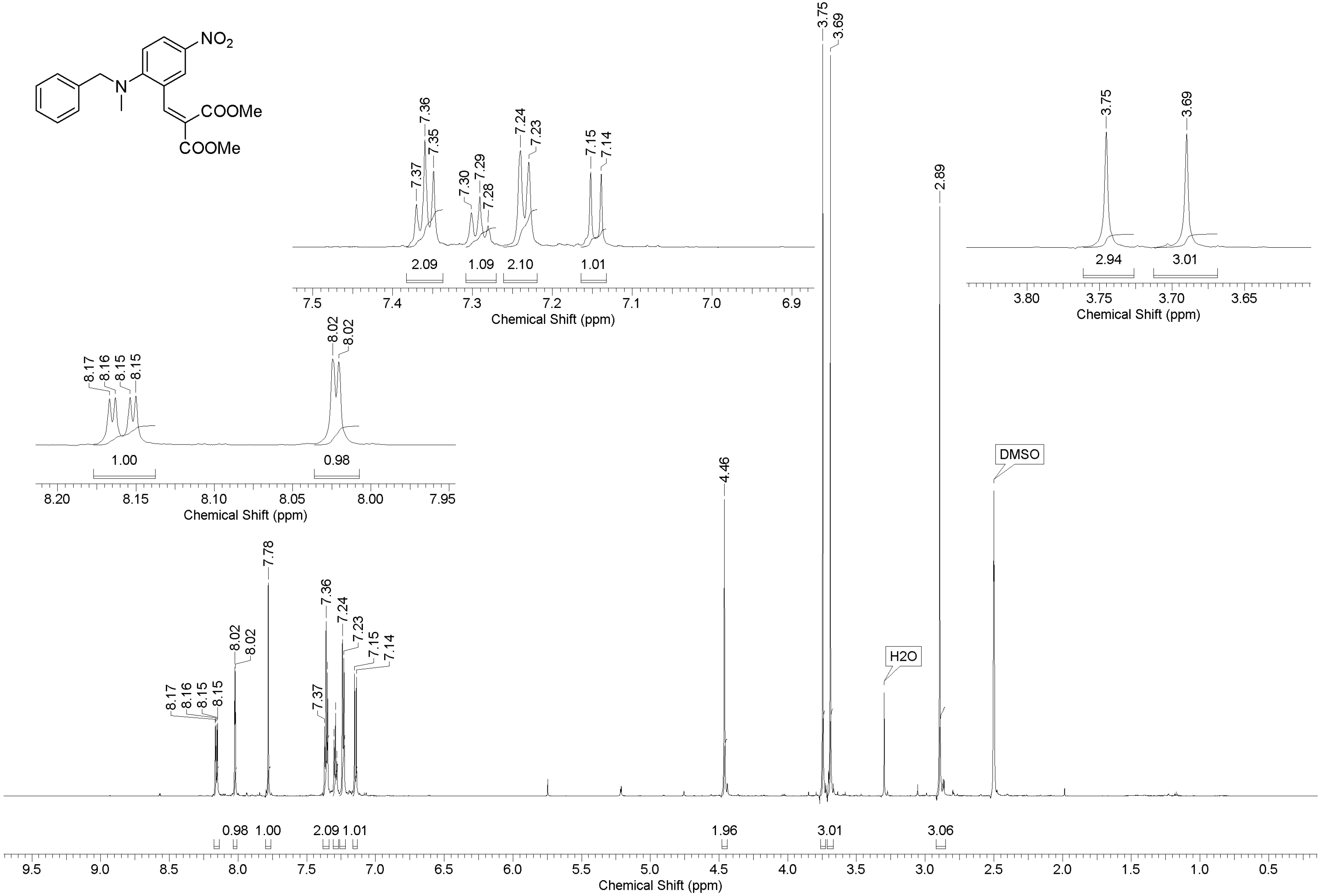

DMSO
H2O
Chemical Shift (ppm)

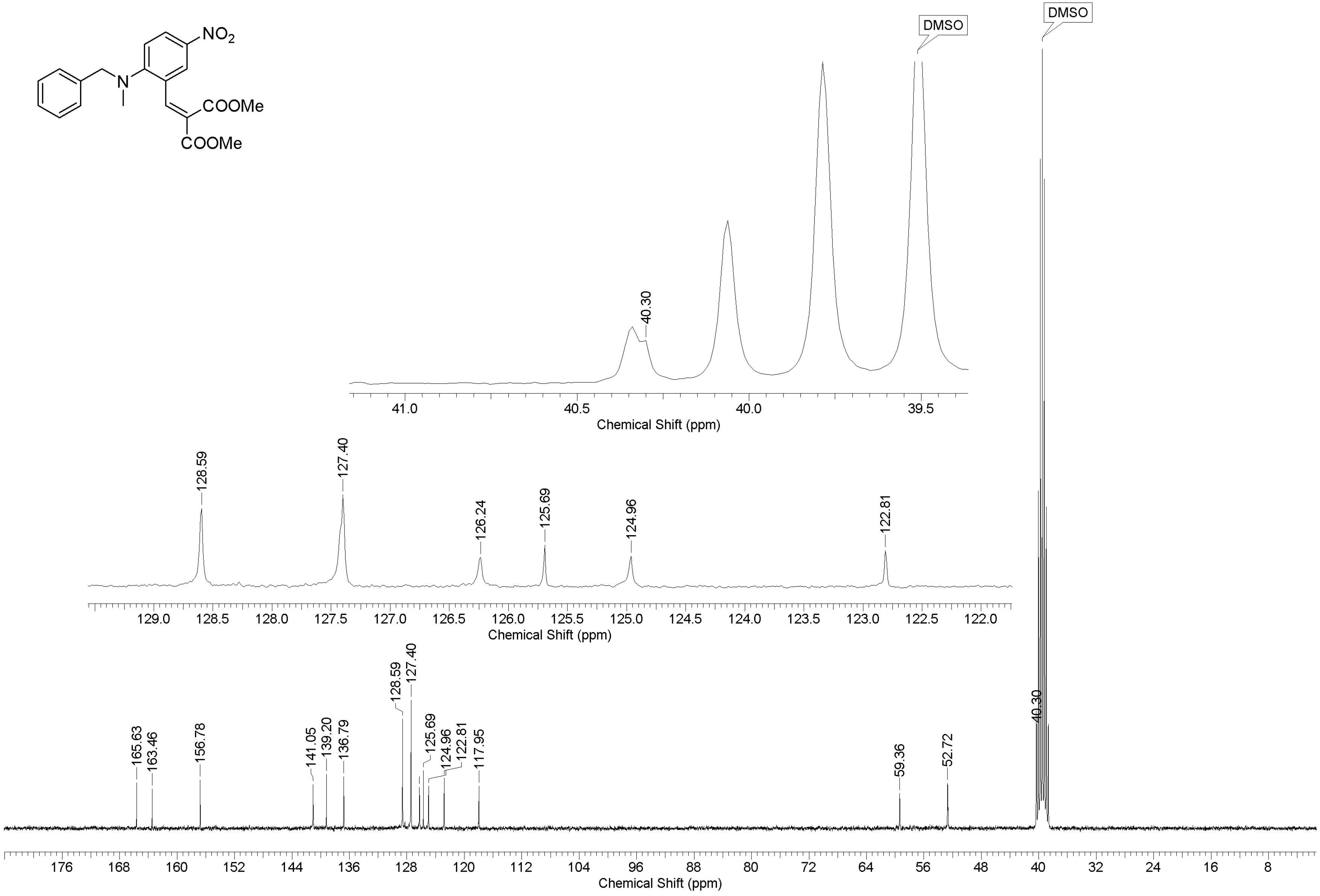
NO2
N
COOMe
COOMe
DMSO
DMSO
40.30
41.0
40.5
40.0
39.5
Chemical Shift (ppm)
128.59
127.40
126.24
125.69
124.96
122.81
129.0
128.5
128.0
127.5
127.0
126.5
126.0
125.5
125.0
124.5
124.0
123.5
123.0
122.5
122.0
Chemical Shift (ppm)
165.63
163.46
156.78
141.05
139.20
136.79
128.59
127.40
125.69
124.96
122.81
117.95
59.36
52.72
40.30
176
168
160
152
144
136
128
120
112
104
96
88
80
72
64
56
48
40
32
24
16
8
Chemical Shift (ppm)

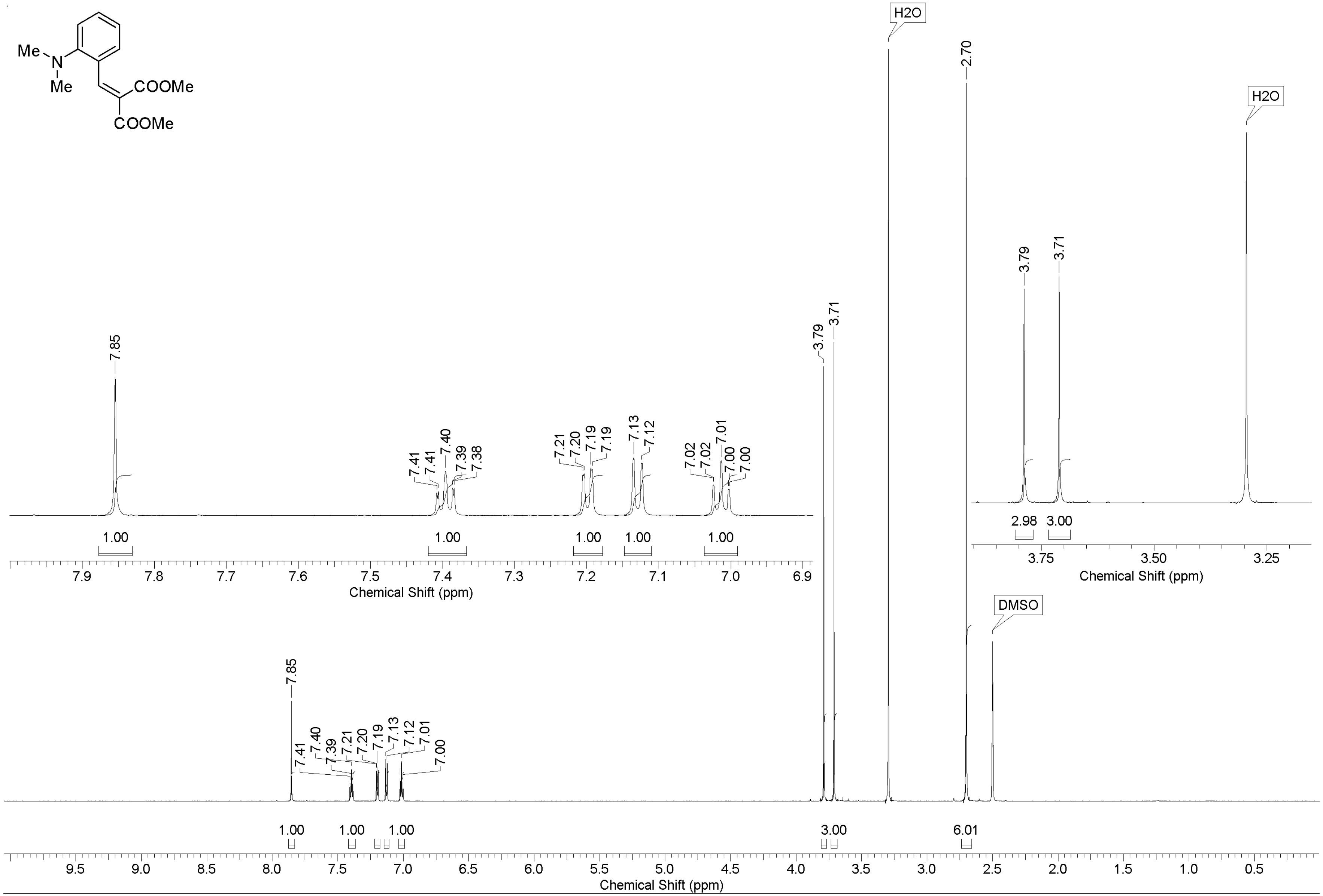
Me
N
Me
COOMe
COOMe
H2O
2.70
H2O
3.79
3.71
7.85
7.41
7.41
7.40
7.39
7.38
7.21
7.20
7.19
7.19
7.13
7.12
7.02
7.02
7.01
7.00
7.00
1.00
1.00
1.00
1.00
1.00
Chemical Shift (ppm)
2.98
3.00
Chemical Shift (ppm)
DMSO
3.00
6.01
Chemical Shift (ppm)

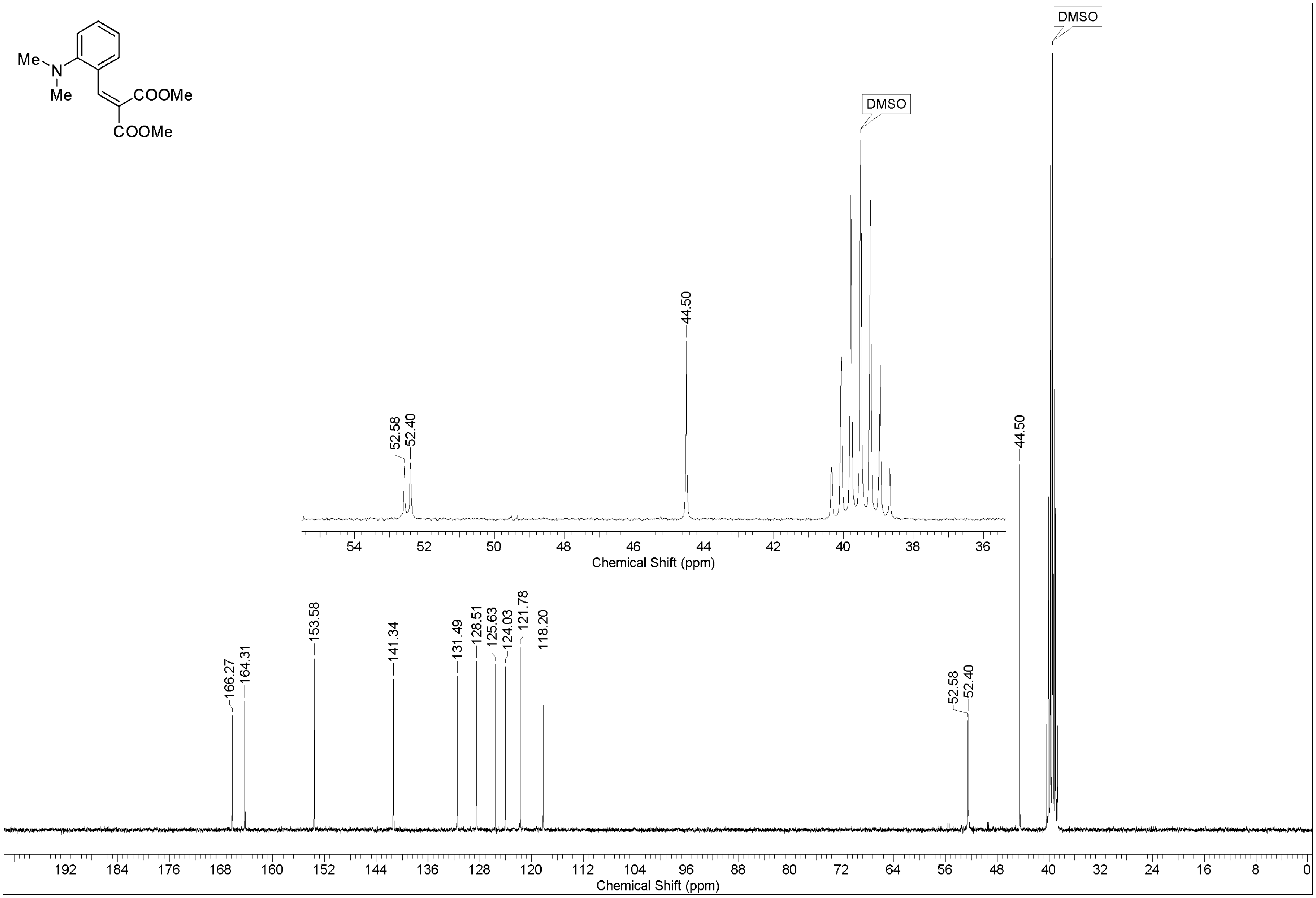
Me
N
Me
COOMe
COOMe
DMSO
DMSO
44.50
52.58
52.40
54
52
50
48
46
44
42
40
38
36
Chemical Shift (ppm)
166.27
164.31
153.58
141.34
131.49
128.51
125.63
124.03
121.78
118.20
52.58
52.40
44.50
192
184
176
168
160
152
144
136
128
120
112
104
96
88
80
72
64
56
48
40
32
24
16
8
0
Chemical Shift (ppm)

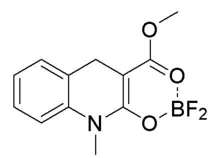
O
O
BF2
N
O

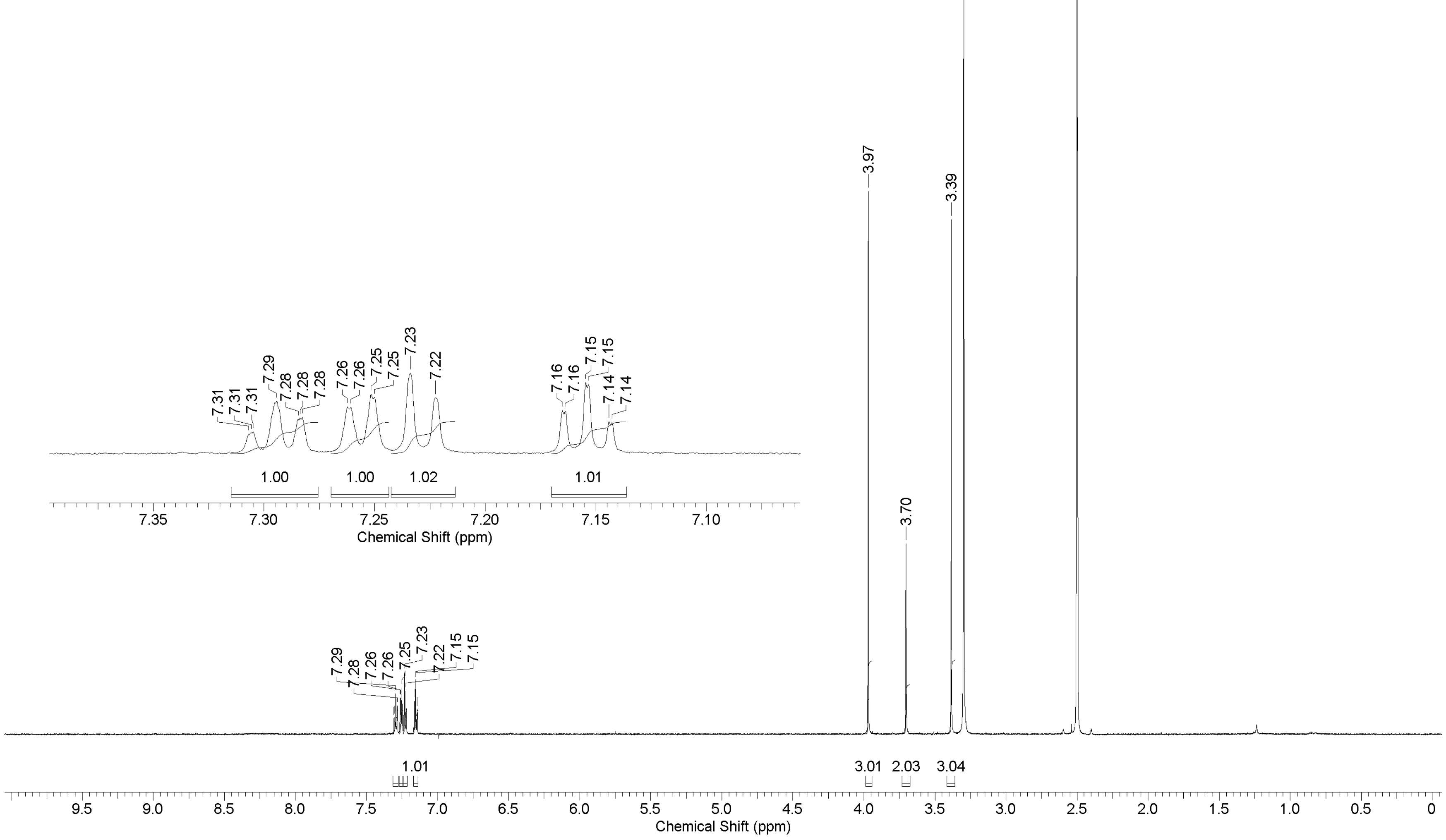
H2O
DMSO
3.97
3.39
3.70
7.31
7.31
7.31
7.29
7.28
7.28
7.28
7.26
7.26
7.25
7.25
7.23
7.22
7.16
7.16
7.15
7.15
7.14
7.14
1.00
1.00
1.02
1.01
7.35
7.30
7.25
7.20
7.15
7.10
Chemical Shift (ppm)
1.01
3.01
2.03
3.04
9.5
9.0
8.5
8.0
7.5
7.0
6.5
6.0
5.5
5.0
4.5
4.0
3.5
3.0
2.5
2.0
1.5
1.0
0.5
0
Chemical Shift (ppm)

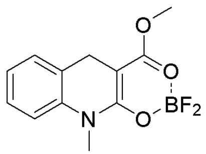
O
O
O
BF2
N

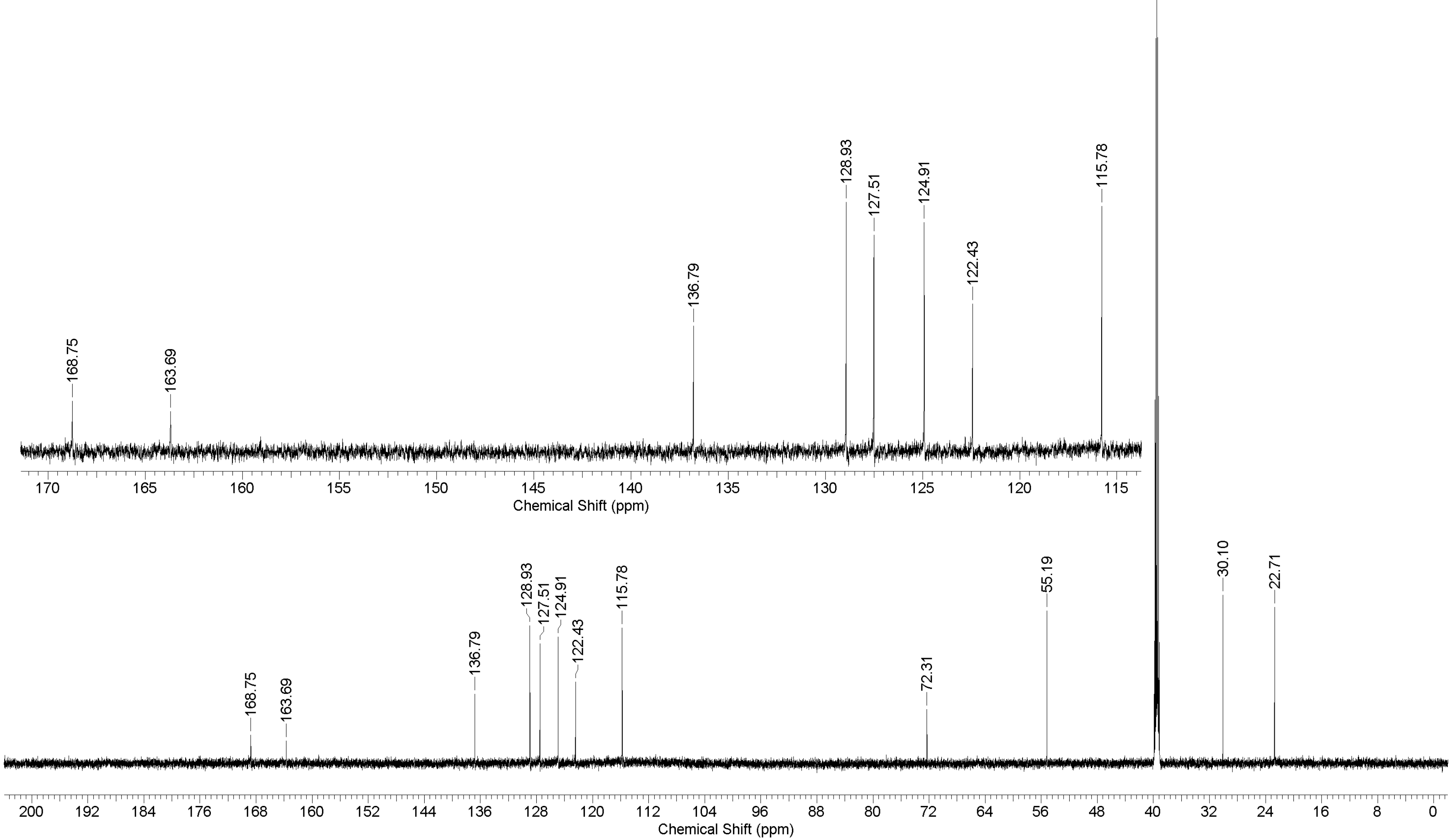
DMSO
168.75
163.69
136.79
128.93
127.51
124.91
122.43
115.78
170 165 160 155 150 145 140 135 130 125 120 115
Chemical Shift (ppm)
168.75
163.69
136.79
128.93
127.51
124.91
122.43
115.78
72.31
55.19
30.10
22.71
200 192 184 176 168 160 152 144 136 128 120 112 104 96 88 80 72 64 56 48 40 32 24 16 8 0
Chemical Shift (ppm)

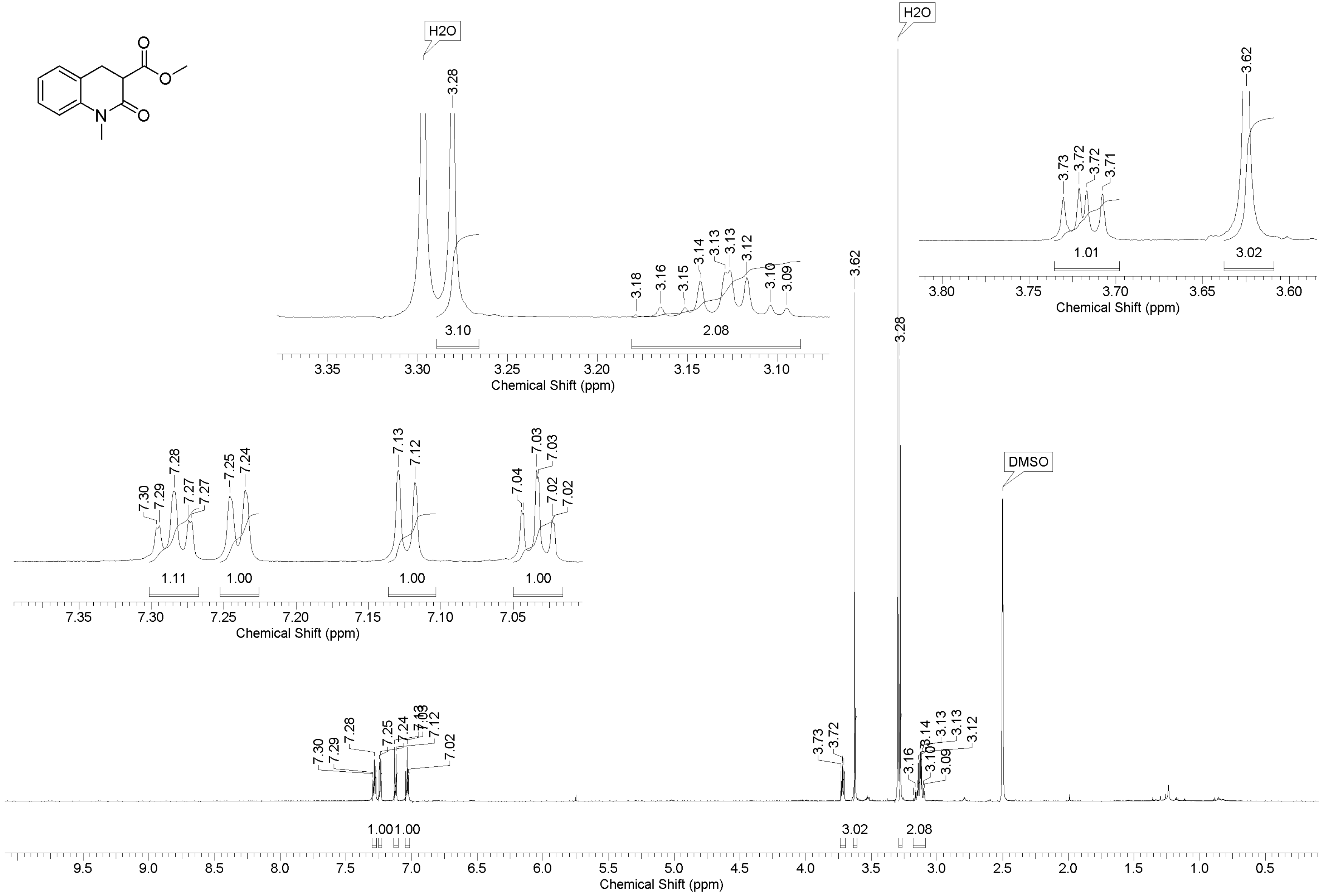
H2O
DMSO
Chemical Shift (ppm)
3.62
3.28
3.73
3.72
3.71
3.18
3.16
3.15
3.14
3.13
3.12
3.10
3.09
7.30
7.29
7.28
7.27
7.25
7.24
7.13
7.12
7.04
7.03
7.02
1.11
1.00
1.01
3.02
3.10
2.08

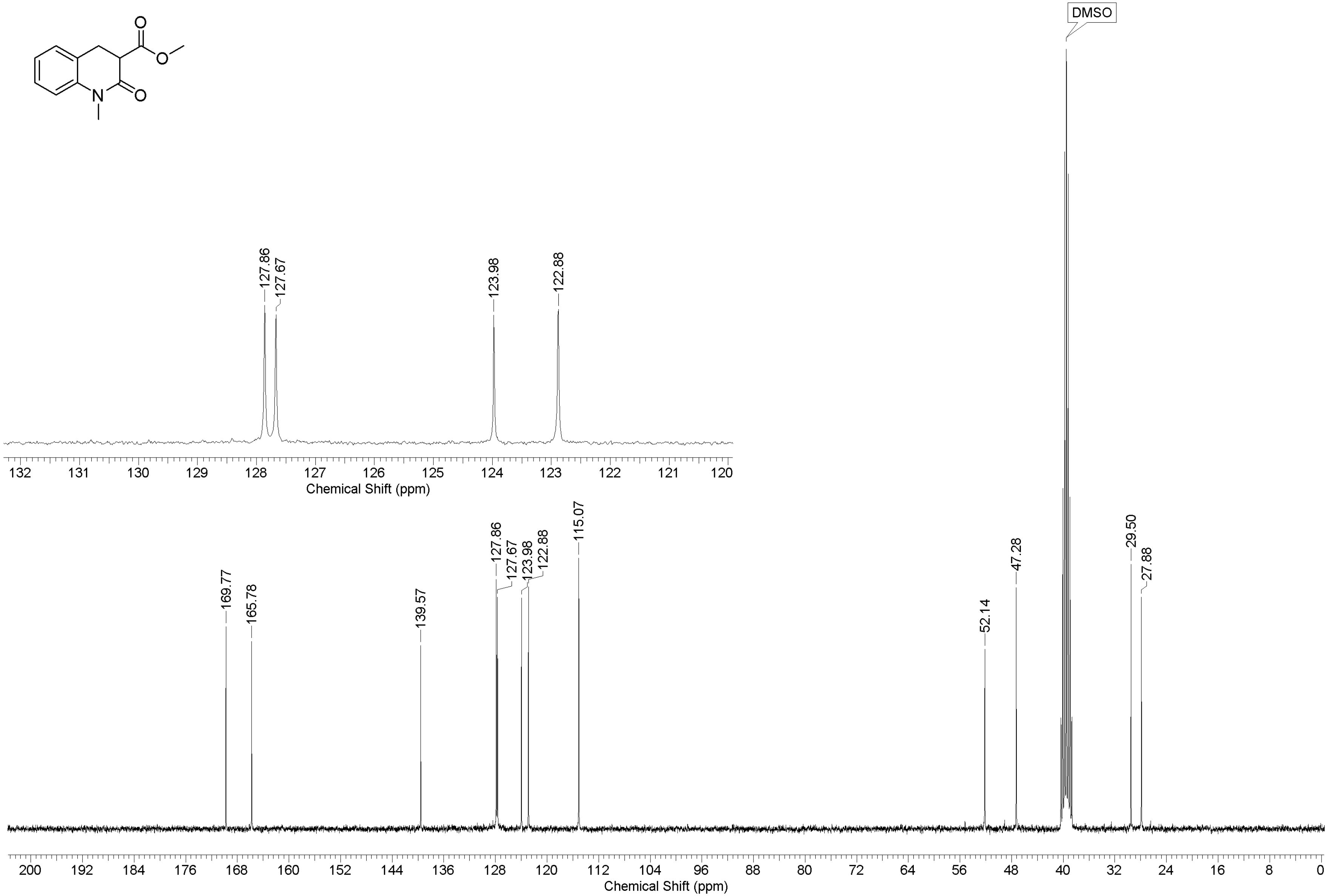

N
O
O
O
DMSO
127.86
127.67
123.98
122.88
132 131 130 129 128 127 126 125 124 123 122 121 120
Chemical Shift (ppm)
169.77
165.78
139.57
127.86
127.67
123.98
122.88
115.07
52.14
47.28
29.50
27.88
200 192 184 176 168 160 152 144 136 128 120 112 104 96 88 80 72 64 56 48 40 32 24 16 8 0
Chemical Shift (ppm)

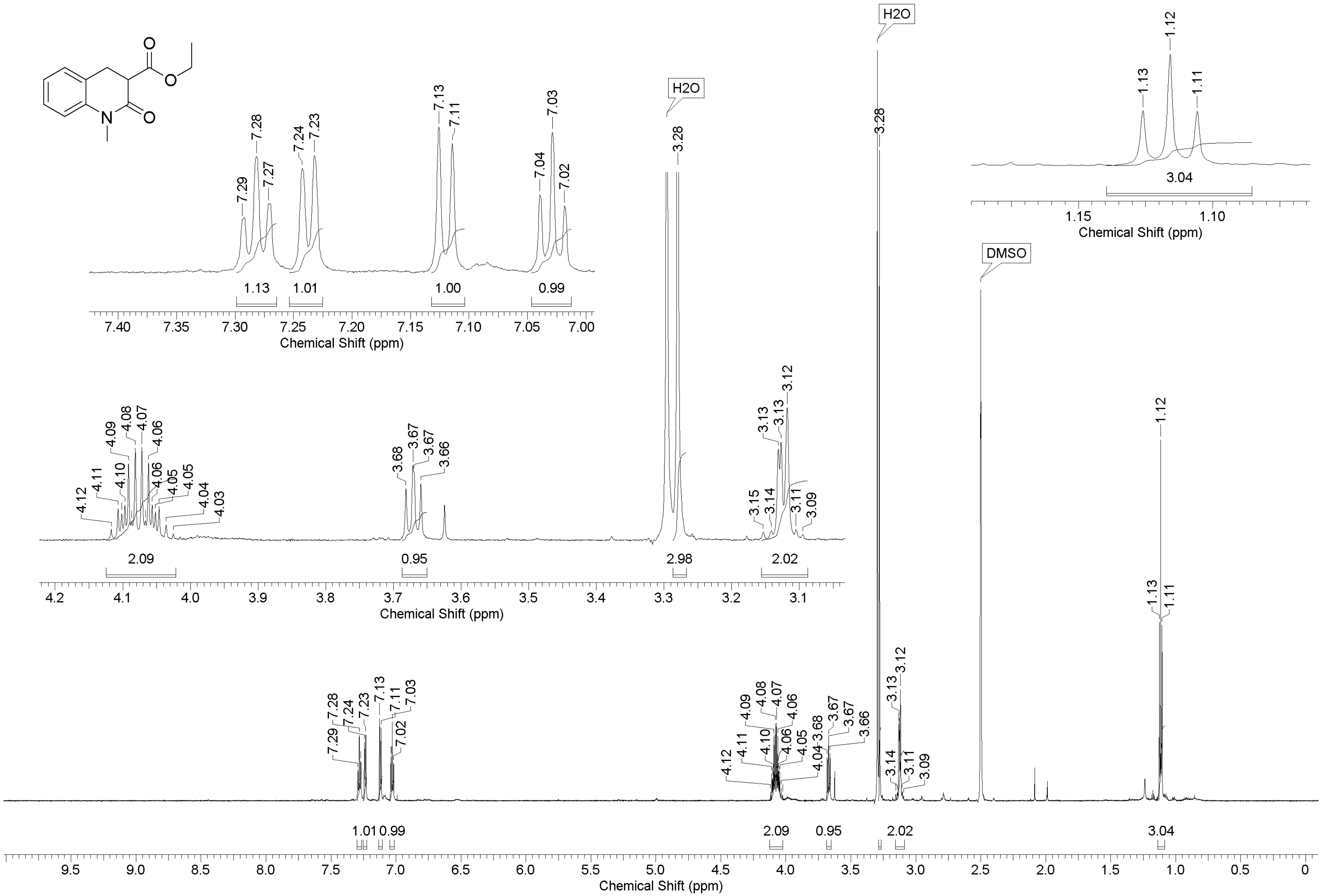
H2O
DMSO
Chemical Shift (ppm)

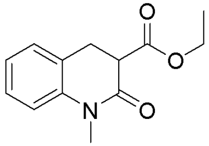

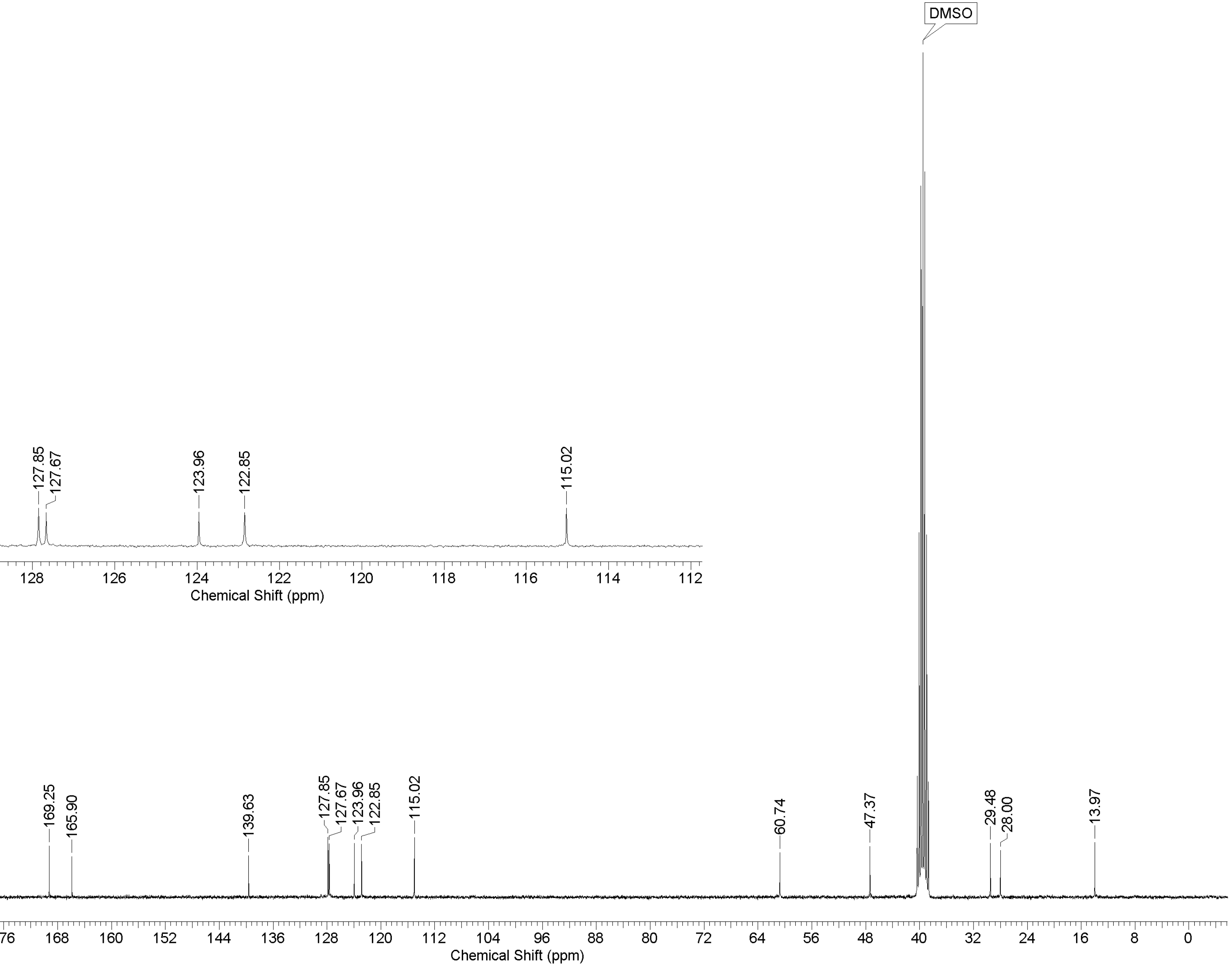
DMSO
127.85
127.67
123.96
122.85
115.02
132
130
128
126
124
122
120
118
116
114
112
Chemical Shift (ppm)
169.25
165.90
139.63
127.85
127.67
123.96
122.85
115.02
60.74
47.37
29.48
28.00
13.97
200
192
184
176
168
160
152
144
136
128
120
112
104
96
88
80
72
64
56
48
40
32
24
16
8
0
Chemical Shift (ppm)

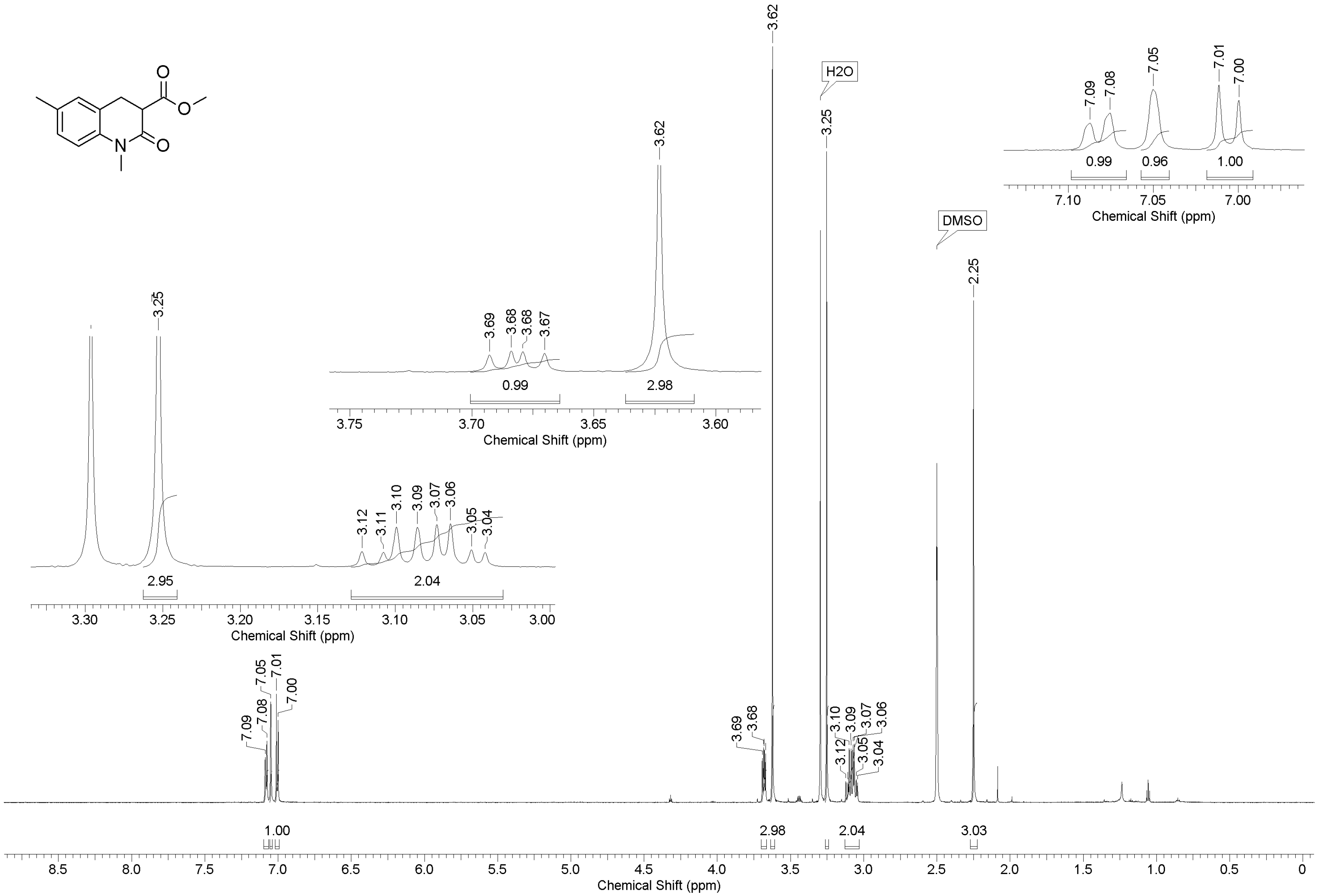
O
O
N
O
3.62
H2O
3.25
DMSO
2.25
7.09
7.08
7.05
7.01
7.00
0.99
0.96
1.00
7.10
7.05
7.00
Chemical Shift (ppm)
3.69
3.68
3.68
3.67
0.99
2.98
3.75
3.70
3.65
3.60
Chemical Shift (ppm)
3.12
3.11
3.10
3.09
3.07
3.06
3.05
3.04
2.95
2.04
3.30
3.25
3.20
3.15
3.10
3.05
3.00
Chemical Shift (ppm)
1.00
2.98
2.04
3.03
8.5
8.0
7.5
7.0
6.5
6.0
5.5
5.0
4.5
4.0
3.5
3.0
2.5
2.0
1.5
1.0
0.5
0
Chemical Shift (ppm)

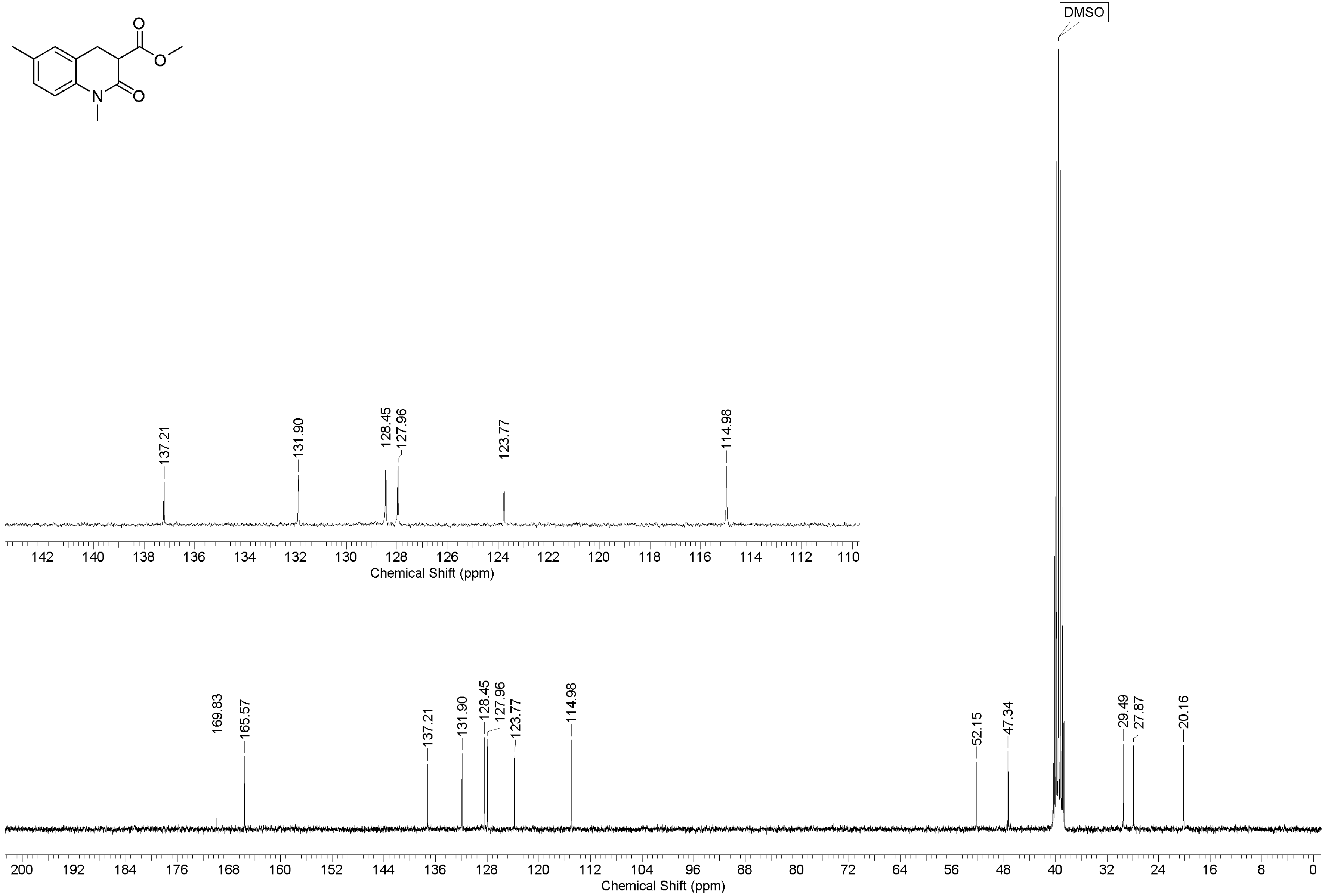

DMSO
137.21
131.90
128.45
127.96
123.77
114.98
142 140 138 136 134 132 130 128 126 124 122 120 118 116 114 112 110
Chemical Shift (ppm)
169.83
165.57
137.21
131.90
128.45
127.96
123.77
114.98
52.15
47.34
29.49
27.87
20.16
200 192 184 176 168 160 152 144 136 128 120 112 104 96 88 80 72 64 56 48 40 32 24 16 8 0
Chemical Shift (ppm)

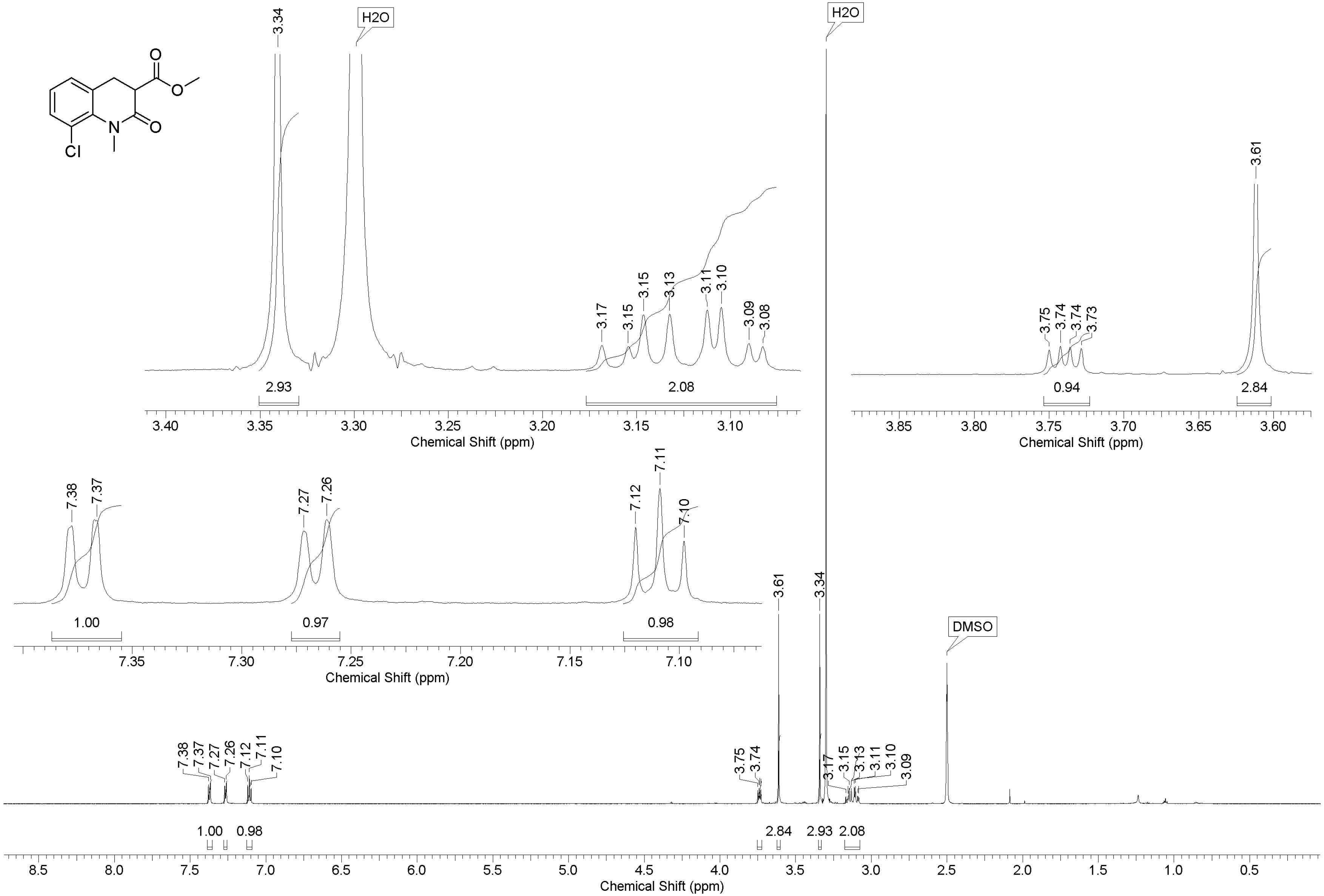
O
O
N
O
Cl
H2O
H2O
DMSO
3.34
3.17
3.15
3.15
3.13
3.11
3.10
3.09
3.08
2.93
2.08
3.40 3.35 3.30 3.25 3.20 3.15 3.10
Chemical Shift (ppm)
3.61
3.75
3.74
3.74
3.73
0.94
2.84
3.85 3.80 3.75 3.70 3.65 3.60
Chemical Shift (ppm)
7.38
7.37
7.27
7.26
7.12
7.11
7.10
1.00
0.97
0.98
7.35 7.30 7.25 7.20 7.15 7.10
Chemical Shift (ppm)
1.00 0.98 2.84 2.93 2.08
8.5 8.0 7.5 7.0 6.5 6.0 5.5 5.0 4.5 4.0 3.5 3.0 2.5 2.0 1.5 1.0 0.5
Chemical Shift (ppm)

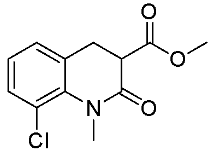

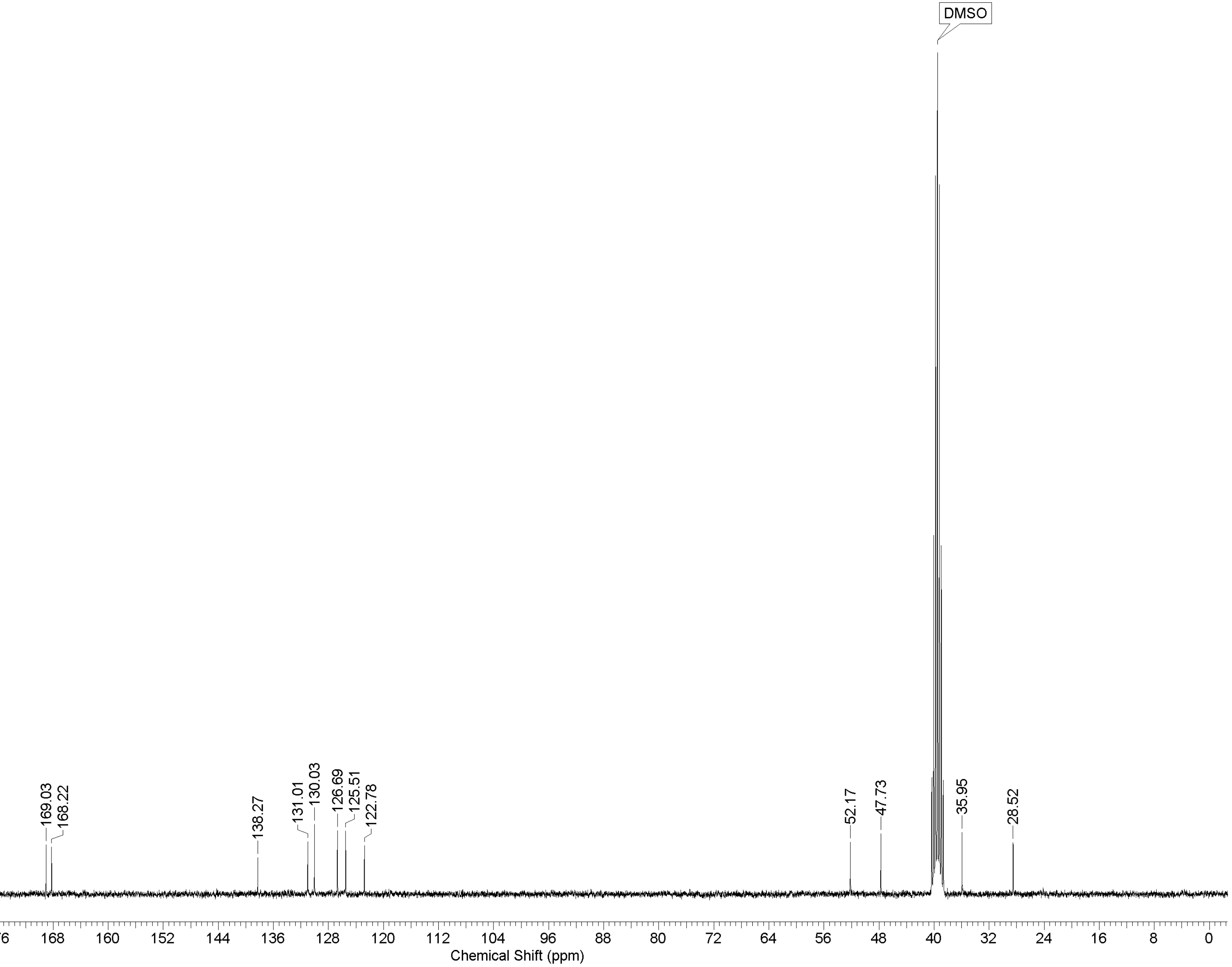

DMSO
169.03
168.22
138.27
131.01
130.03
126.69
125.51
122.78
52.17
47.73
35.95
28.52
200 192 184 176 168 160 152 144 136 128 120 112 104 96 88 80 72 64 56 48 40 32 24 16 8 0
Chemical Shift (ppm)

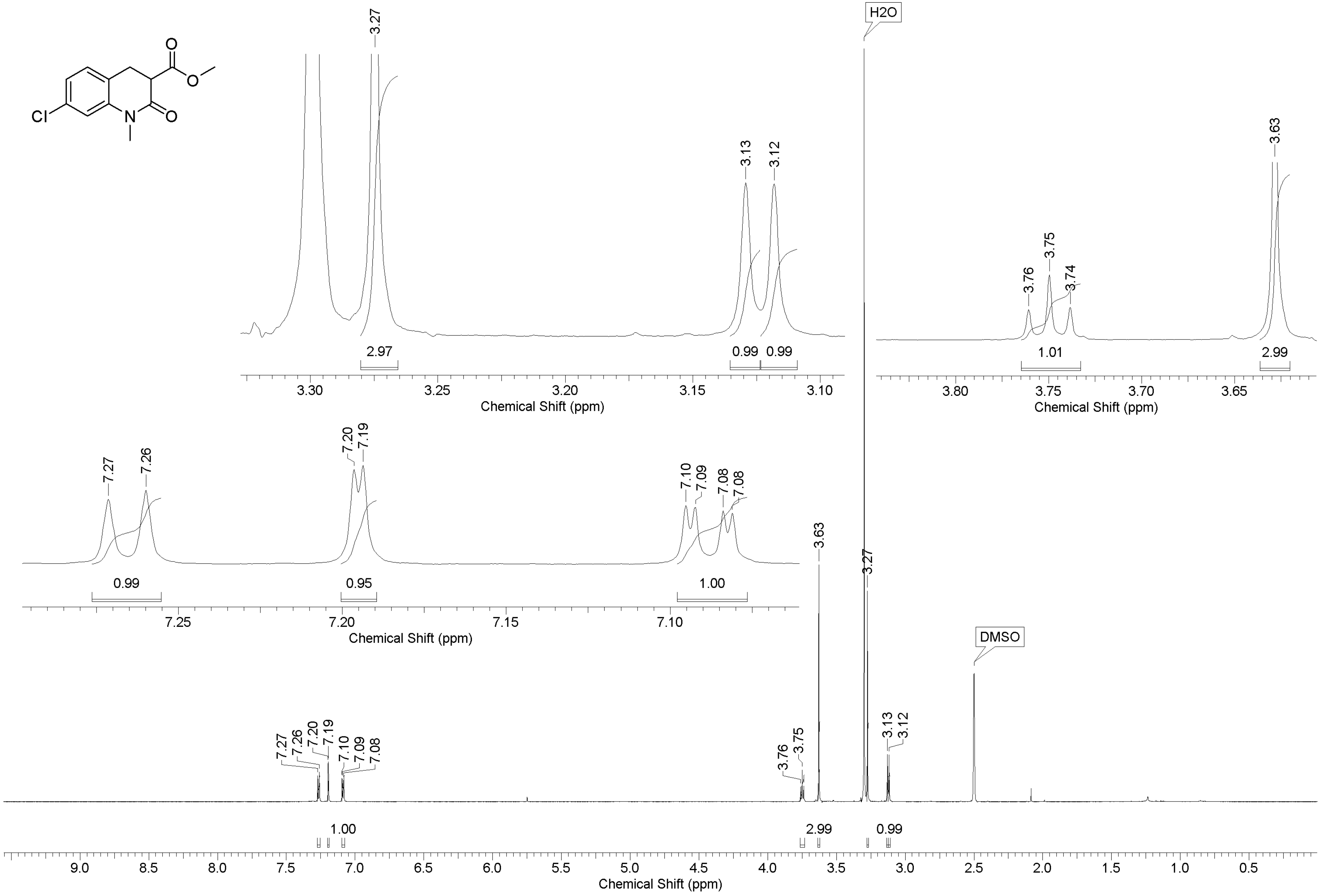
Cl
N
O
O
O
H2O
DMSO
Chemical Shift (ppm)
3.27
3.13
3.12
3.76
3.75
3.74
3.63
7.27
7.26
7.20
7.19
7.10
7.09
7.08
7.08
2.97
0.99
0.99
1.01
2.99
0.95
1.00

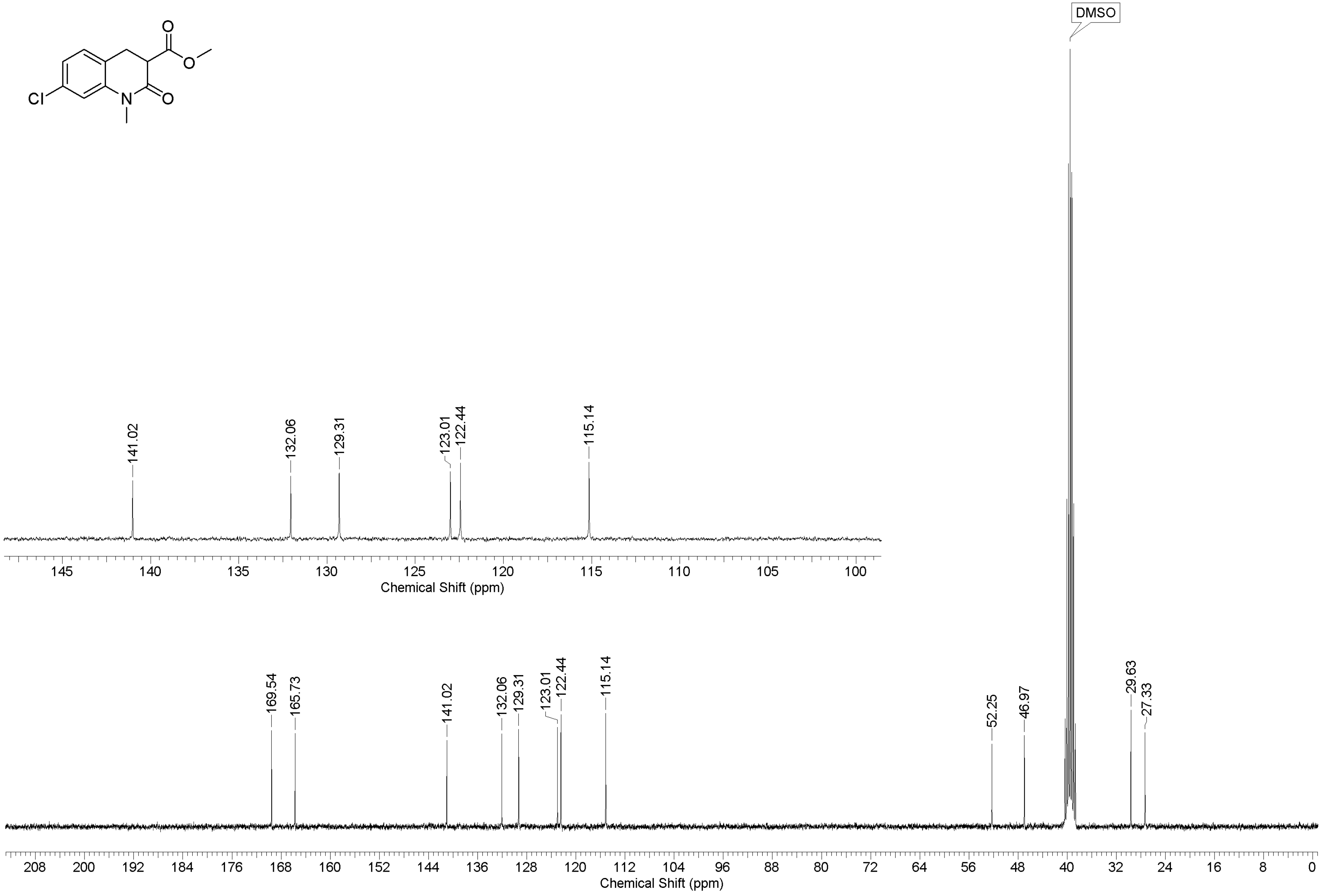

Cl
N
O
O
O
DMSO
141.02
132.06
129.31
123.01
122.44
115.14
145 140 135 130 125 120 115 110 105 100
Chemical Shift (ppm)
169.54
165.73
141.02
132.06
129.31
123.01
122.44
115.14
52.25
46.97
29.63
27.33
208 200 192 184 176 168 160 152 144 136 128 120 112 104 96 88 80 72 64 56 48 40 32 24 16 8 0
Chemical Shift (ppm)

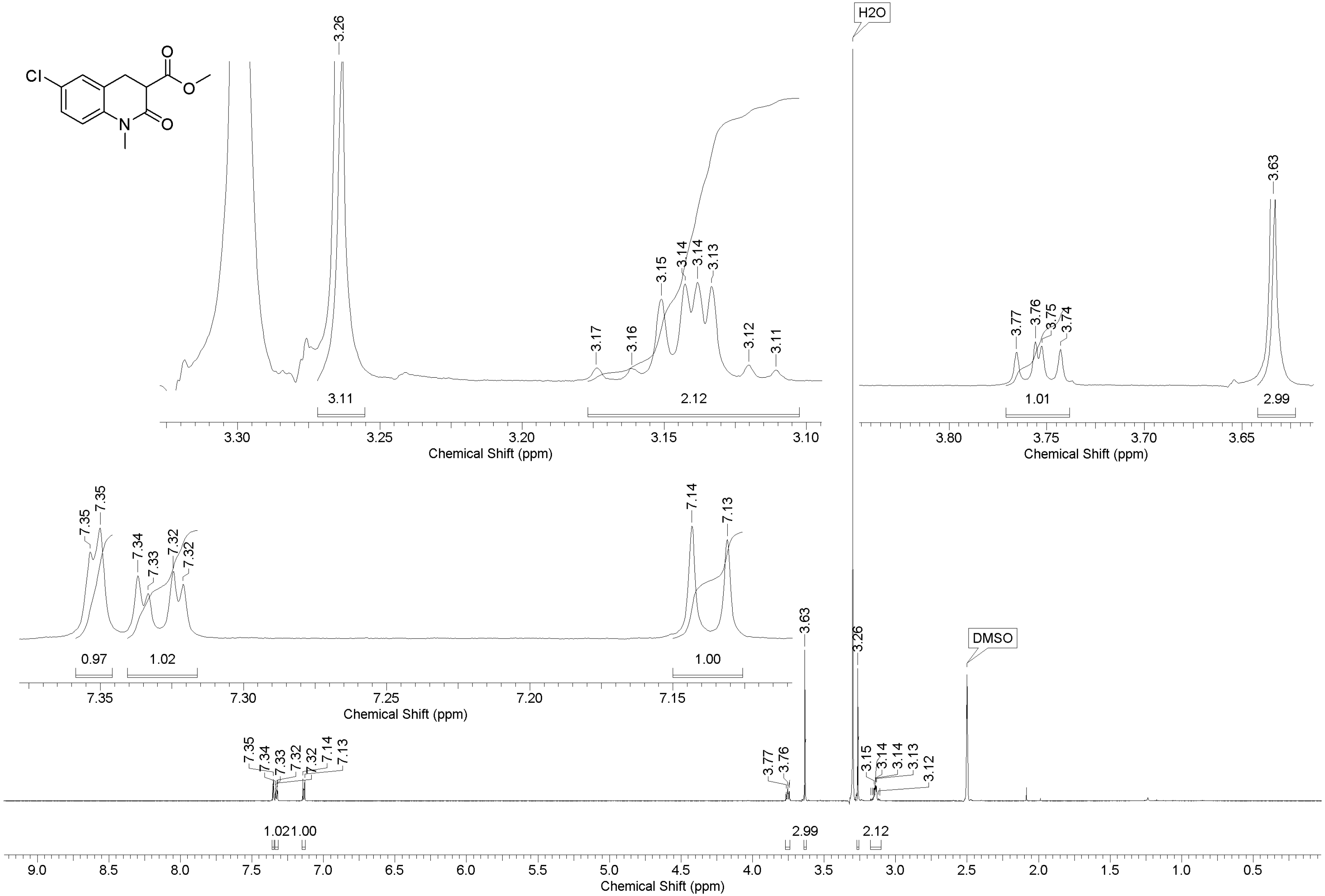
Cl
N
O
O
O
H2O
DMSO
3.26
3.17
3.16
3.15
3.14
3.14
3.13
3.12
3.11
3.11
2.12
3.30
3.25
3.20
3.15
3.10
Chemical Shift (ppm)
3.77
3.76
3.75
3.74
3.63
1.01
2.99
3.80
3.75
3.70
3.65
Chemical Shift (ppm)
7.35
7.35
7.34
7.33
7.32
7.32
7.14
7.13
0.97
1.02
1.00
7.35
7.30
7.25
7.20
7.15
Chemical Shift (ppm)
3.63
3.26
7.35
7.34
7.33
7.32
7.32
7.14
7.13
3.77
3.76
3.15
3.14
3.14
3.13
3.12
1.02
1.00
2.99
2.12
9.0
8.5
8.0
7.5
7.0
6.5
6.0
5.5
5.0
4.5
4.0
3.5
3.0
2.5
2.0
1.5
1.0
0.5
Chemical Shift (ppm)

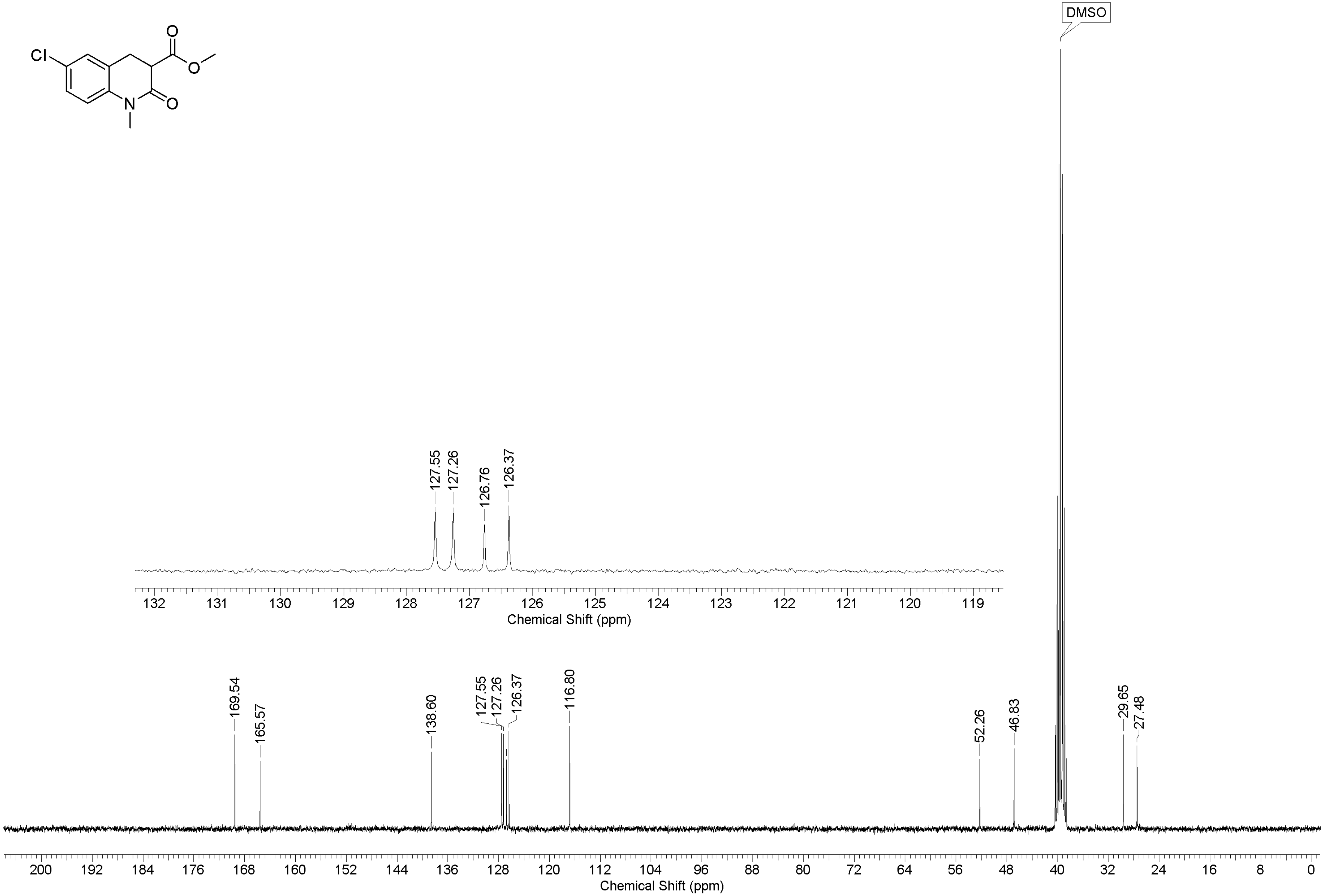
DMSO
127.55
127.26
126.76
126.37
132 131 130 129 128 127 126 125 124 123 122 121 120 119
Chemical Shift (ppm)
169.54
165.57
138.60
127.55
127.26
126.37
116.80
52.26
46.83
29.65
27.48
200 192 184 176 168 160 152 144 136 128 120 112 104 96 88 80 72 64 56 48 40 32 24 16 8 0
Chemical Shift (ppm)

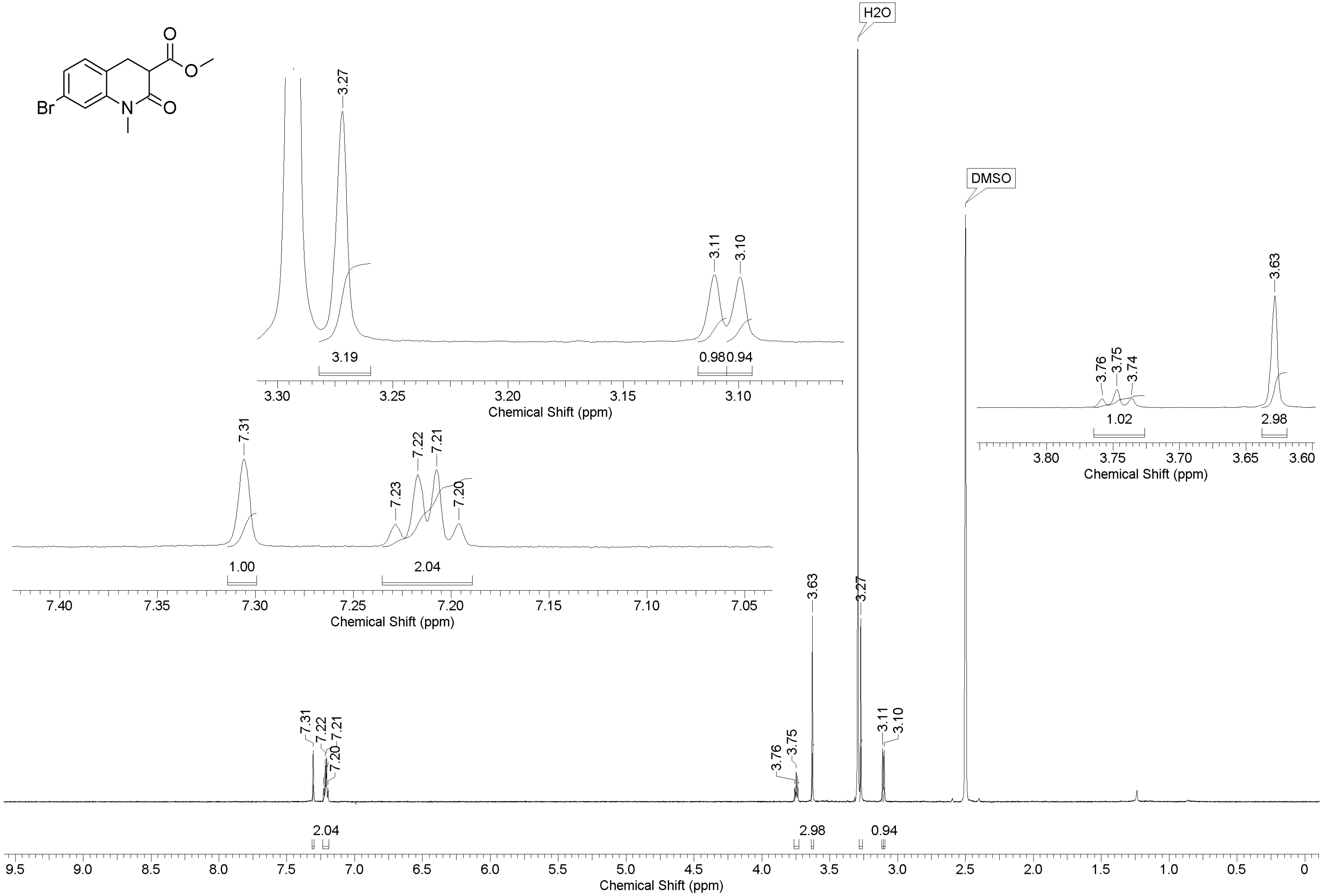
Br
N
O
O
O
H2O
DMSO
3.27
3.11
3.10
3.19
0.98
0.94
3.30
3.25
3.20
3.15
3.10
Chemical Shift (ppm)
3.76
3.75
3.74
3.63
1.02
2.98
3.80
3.75
3.70
3.65
3.60
Chemical Shift (ppm)
7.31
7.23
7.22
7.21
7.20
1.00
2.04
7.40
7.35
7.30
7.25
7.20
7.15
7.10
7.05
Chemical Shift (ppm)
7.31
7.22
7.21
7.20
3.76
3.75
3.63
3.27
3.11
3.10
2.04
2.98
0.94
9.5
9.0
8.5
8.0
7.5
7.0
6.5
6.0
5.5
5.0
4.5
4.0
3.5
3.0
2.5
2.0
1.5
1.0
0.5
0
Chemical Shift (ppm)

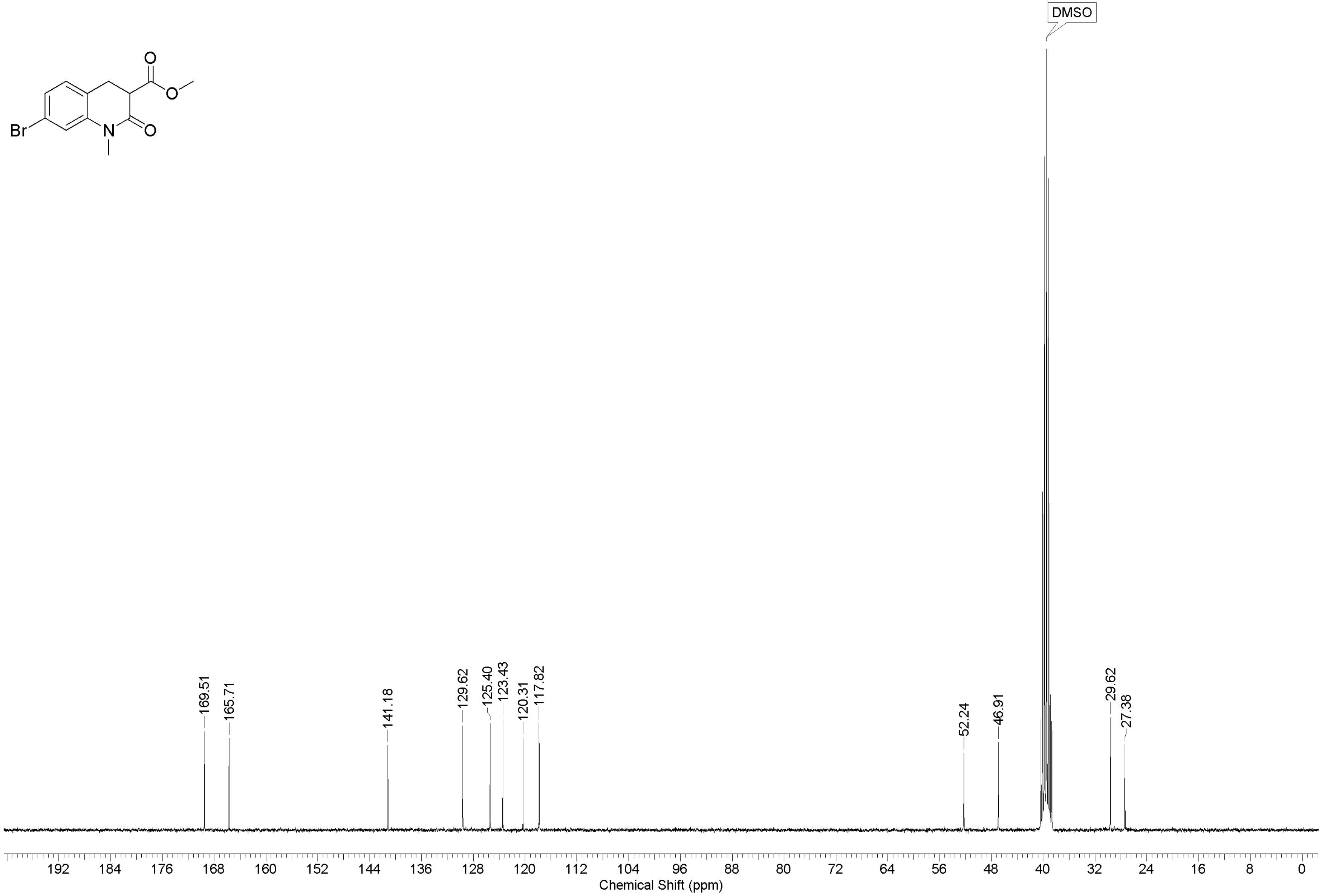
Br
N
O
O
O
DMSO
169.51
165.71
141.18
129.62
125.40
123.43
120.31
117.82
52.24
46.91
29.62
27.38
192 184 176 168 160 152 144 136 128 120 112 104 96 88 80 72 64 56 48 40 32 24 16 8 0
Chemical Shift (ppm)

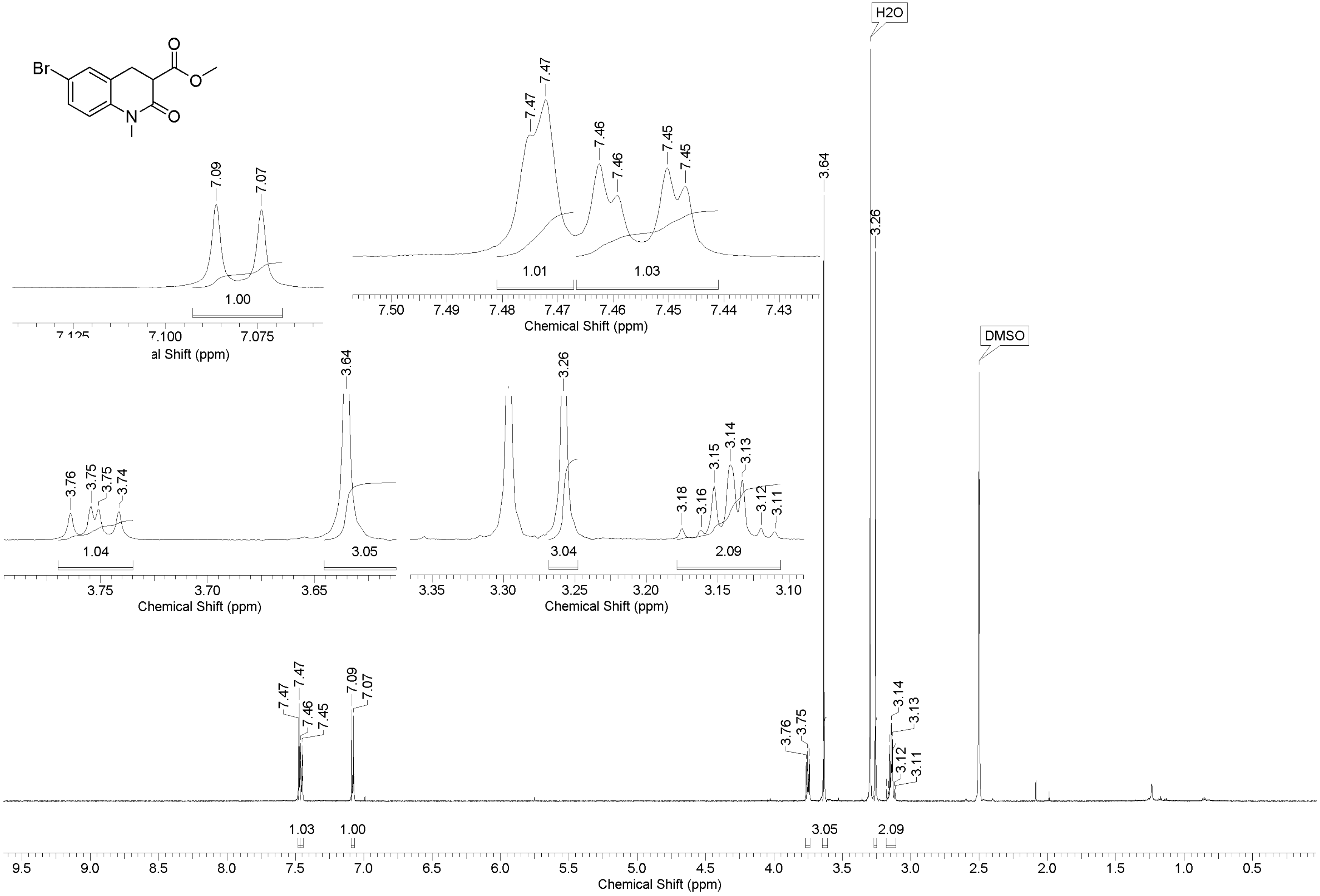
Br
N
O
O
O
7.09
7.07
1.00
7.125
7.100
7.075
al Shift (ppm)
7.47
7.47
7.46
7.46
7.45
7.45
1.01
1.03
7.50
7.49
7.48
7.47
7.46
7.45
7.44
7.43
Chemical Shift (ppm)
H2O
3.64
3.26
DMSO
3.76
3.75
3.75
3.74
1.04
3.05
3.75
3.70
3.65
Chemical Shift (ppm)
3.18
3.16
3.15
3.14
3.13
3.12
3.11
3.04
2.09
3.35
3.30
3.25
3.20
3.15
3.10
Chemical Shift (ppm)
1.03
1.00
3.05
2.09
9.5
9.0
8.5
8.0
7.5
7.0
6.5
6.0
5.5
5.0
4.5
4.0
3.5
3.0
2.5
2.0
1.5
1.0
0.5
Chemical Shift (ppm)

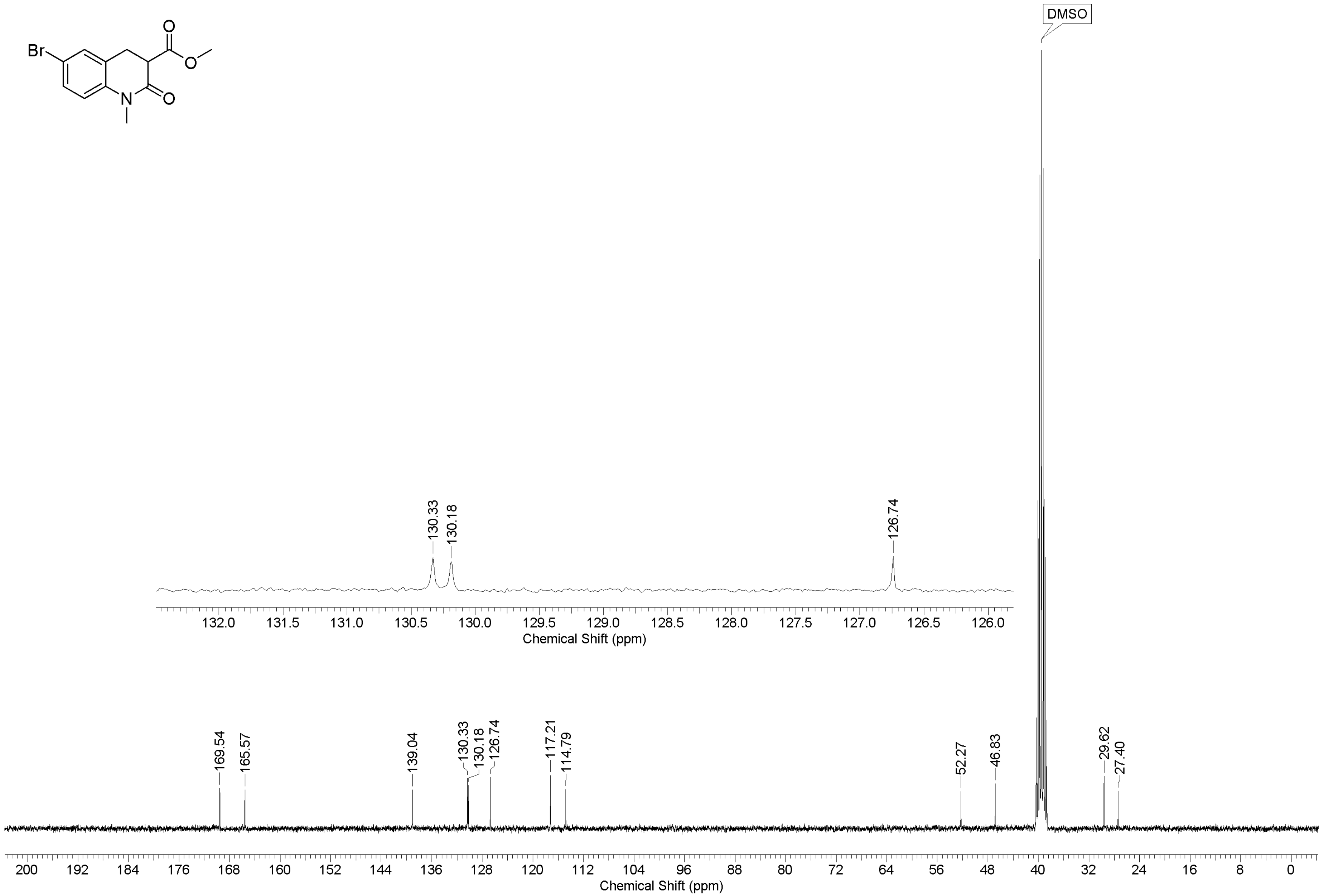
Br
N
O
O
O
DMSO
130.33
130.18
126.74
132.0 131.5 131.0 130.5 130.0 129.5 129.0 128.5 128.0 127.5 127.0 126.5 126.0
Chemical Shift (ppm)
169.54
165.57
139.04
130.33
130.18
126.74
117.21
114.79
52.27
46.83
29.62
27.40
200 192 184 176 168 160 152 144 136 128 120 112 104 96 88 80 72 64 56 48 40 32 24 16 8 0
Chemical Shift (ppm)

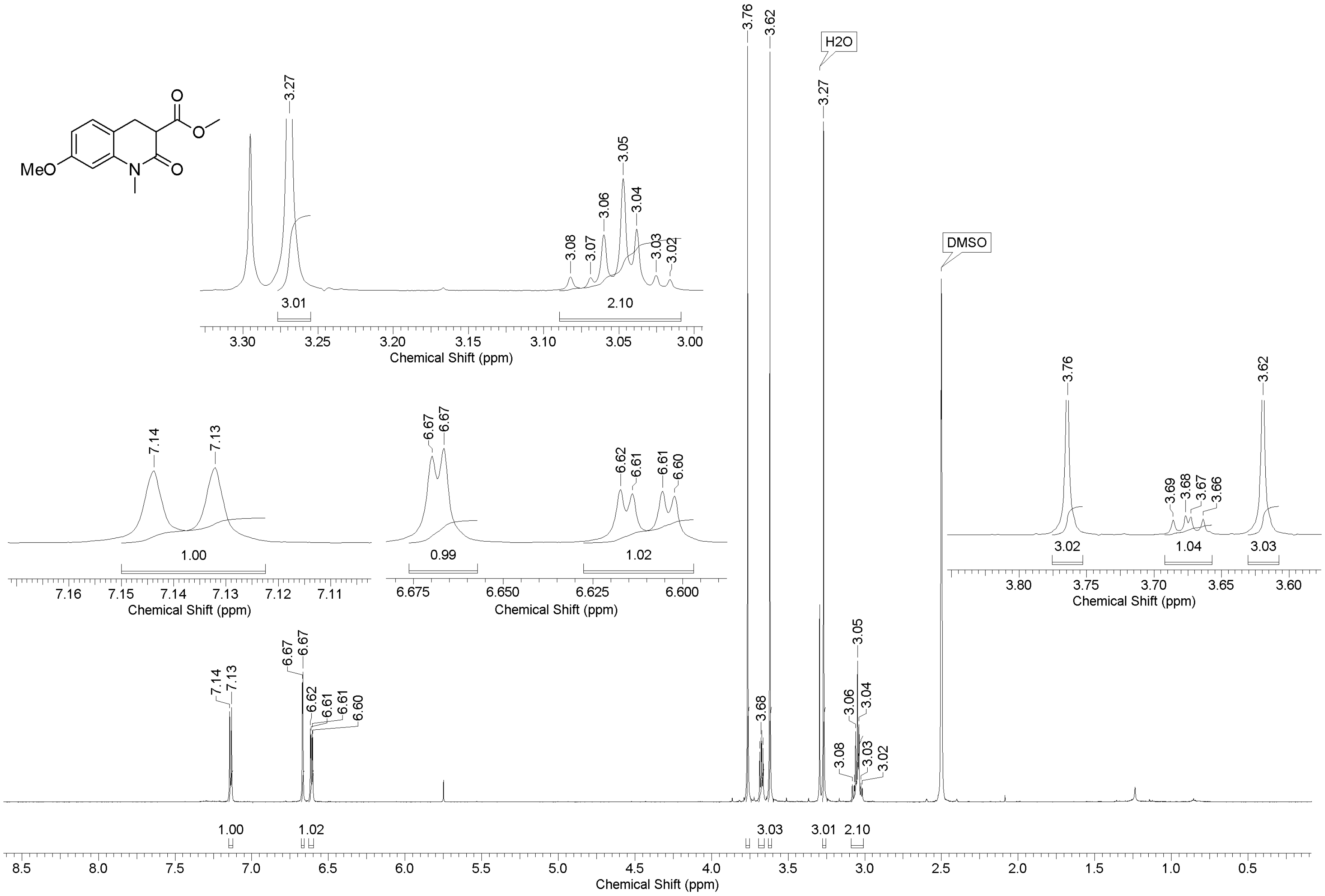
MeO
N
O
O
O
3.27
3.05
3.06
3.04
3.08
3.07
3.03
3.02
3.01
2.10
3.30 3.25 3.20 3.15 3.10 3.05 3.00
Chemical Shift (ppm)
7.14
7.13
1.00
7.16 7.15 7.14 7.13 7.12 7.11
Chemical Shift (ppm)
6.67
6.67
6.62
6.61
6.61
6.60
0.99
1.02
6.675 6.650 6.625 6.600
Chemical Shift (ppm)
3.76
3.62
3.69
3.68
3.67
3.66
3.02
1.04
3.03
3.80 3.75 3.70 3.65 3.60
Chemical Shift (ppm)
H2O
DMSO
3.76
3.62
3.27
3.68
3.05
3.06
3.04
3.08
3.03
3.02
7.14
7.13
6.67
6.67
6.62
6.61
6.61
6.60
1.00
1.02
3.03
3.01
2.10
8.5 8.0 7.5 7.0 6.5 6.0 5.5 5.0 4.5 4.0 3.5 3.0 2.5 2.0 1.5 1.0 0.5
Chemical Shift (ppm)

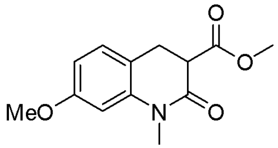
MeO
N
O
O
O

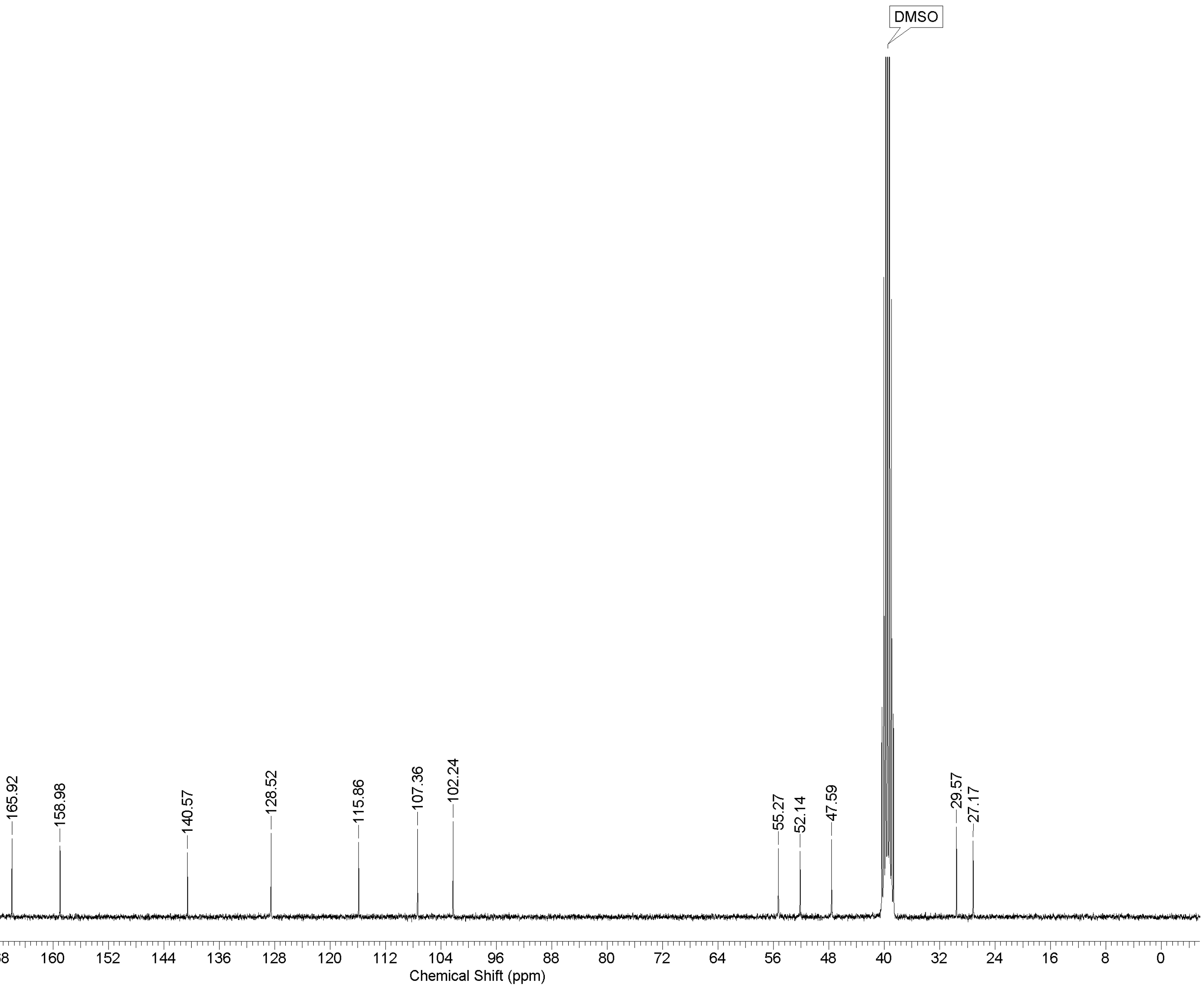
DMSO
169.79
165.92
158.98
140.57
128.52
115.86
107.36
102.24
55.27
52.14
47.59
29.57
27.17
200 192 184 176 168 160 152 144 136 128 120 112 104 96 88 80 72 64 56 48 40 32 24 16 8 0
Chemical Shift (ppm)

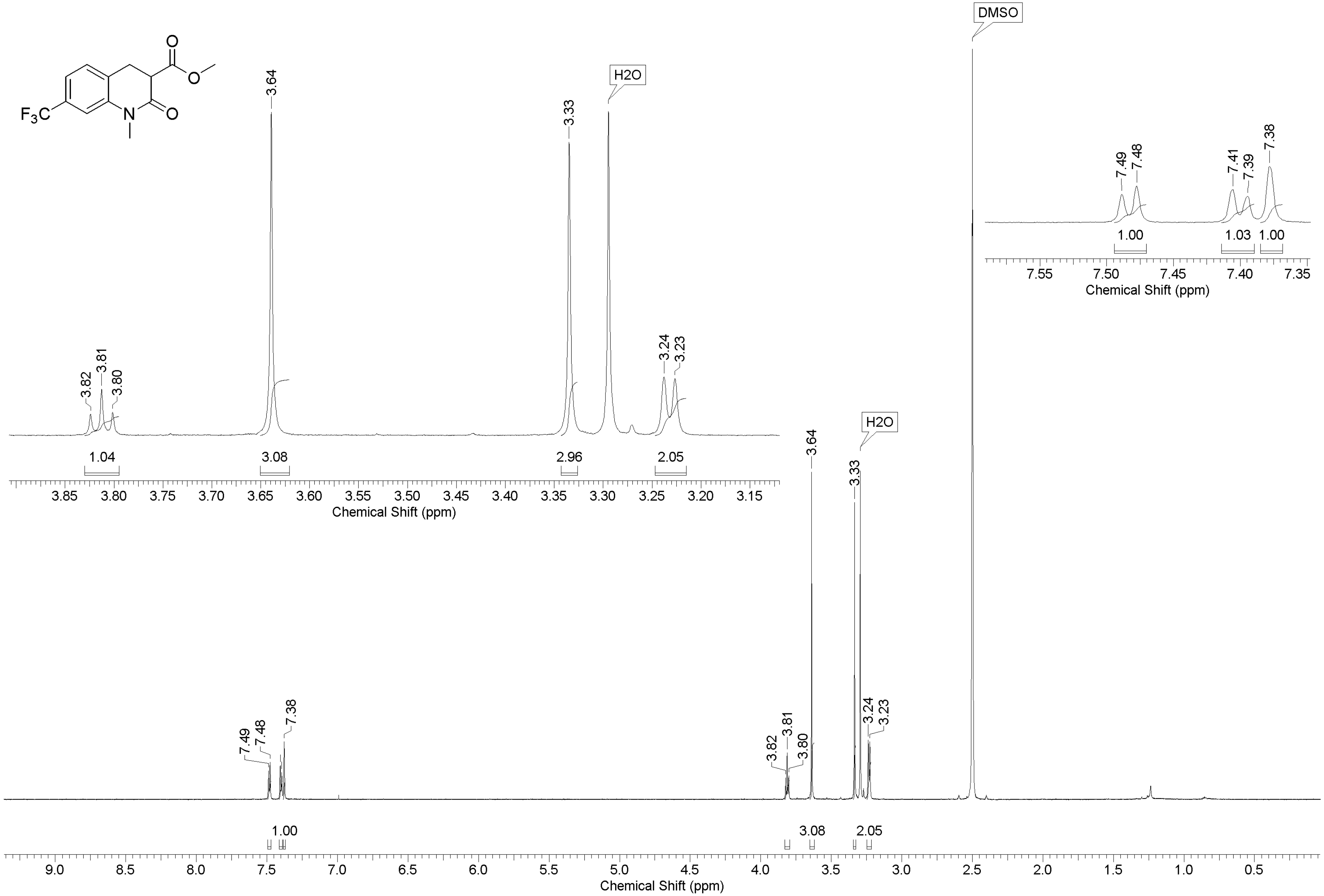
F3C
N
O
O
O
DMSO
H2O
3.64
3.33
3.82
3.81
3.80
3.24
3.23
1.04
3.08
2.96
2.05
Chemical Shift (ppm)
7.49
7.48
7.41
7.39
7.38
1.00
1.03
1.00
Chemical Shift (ppm)
Chemical Shift (ppm)

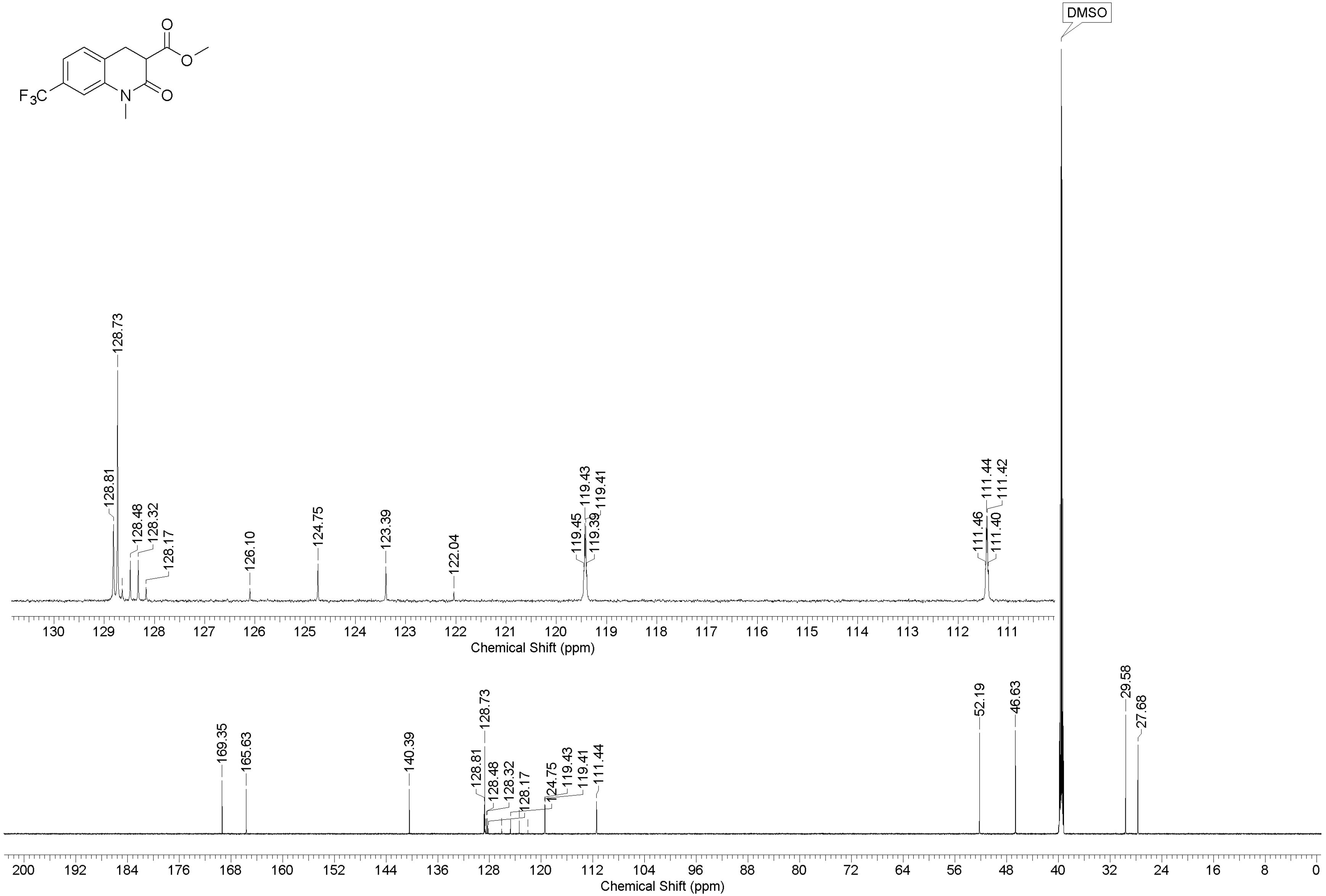
DMSO
128.81
128.73
128.48
128.32
128.17
126.10
124.75
123.39
122.04
119.45
119.43
119.41
119.39
111.46
111.44
111.42
111.40
Chemical Shift (ppm)
169.35
165.63
140.39
52.19
46.63
29.58
27.68
Chemical Shift (ppm)

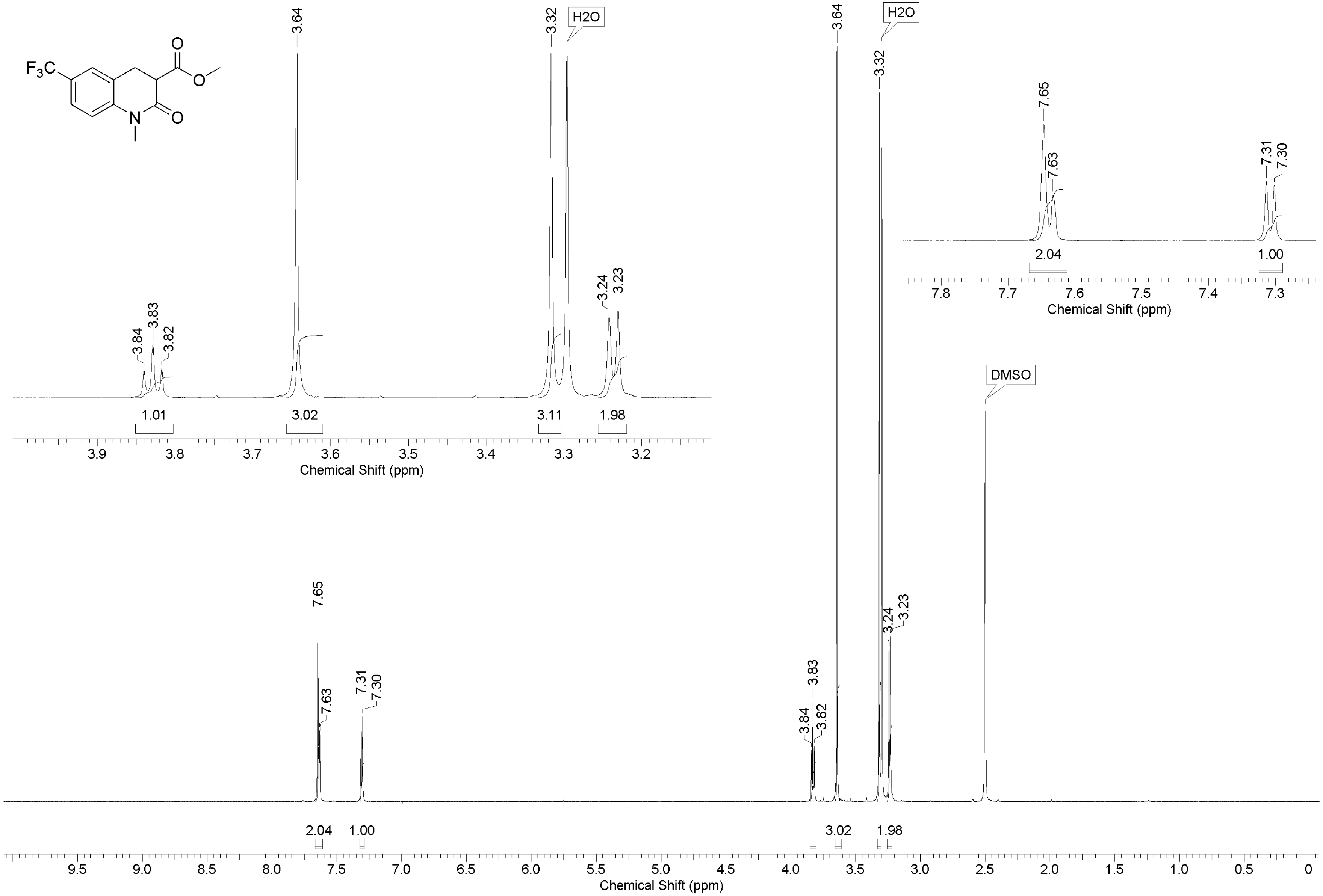

F3C
O
O
N
O
3.64
3.32
H2O
3.84
3.83
3.82
3.24
3.23
1.01
3.02
3.11
1.98
3.9
3.8
3.7
3.6
3.5
3.4
3.3
3.2
Chemical Shift (ppm)
7.65
7.63
7.31
7.30
2.04
1.00
7.8
7.7
7.6
7.5
7.4
7.3
Chemical Shift (ppm)
DMSO
2.04
1.00
3.02
1.98
9.5
9.0
8.5
8.0
7.5
7.0
6.5
6.0
5.5
5.0
4.5
4.0
3.5
3.0
2.5
2.0
1.5
1.0
0.5
0
Chemical Shift (ppm)

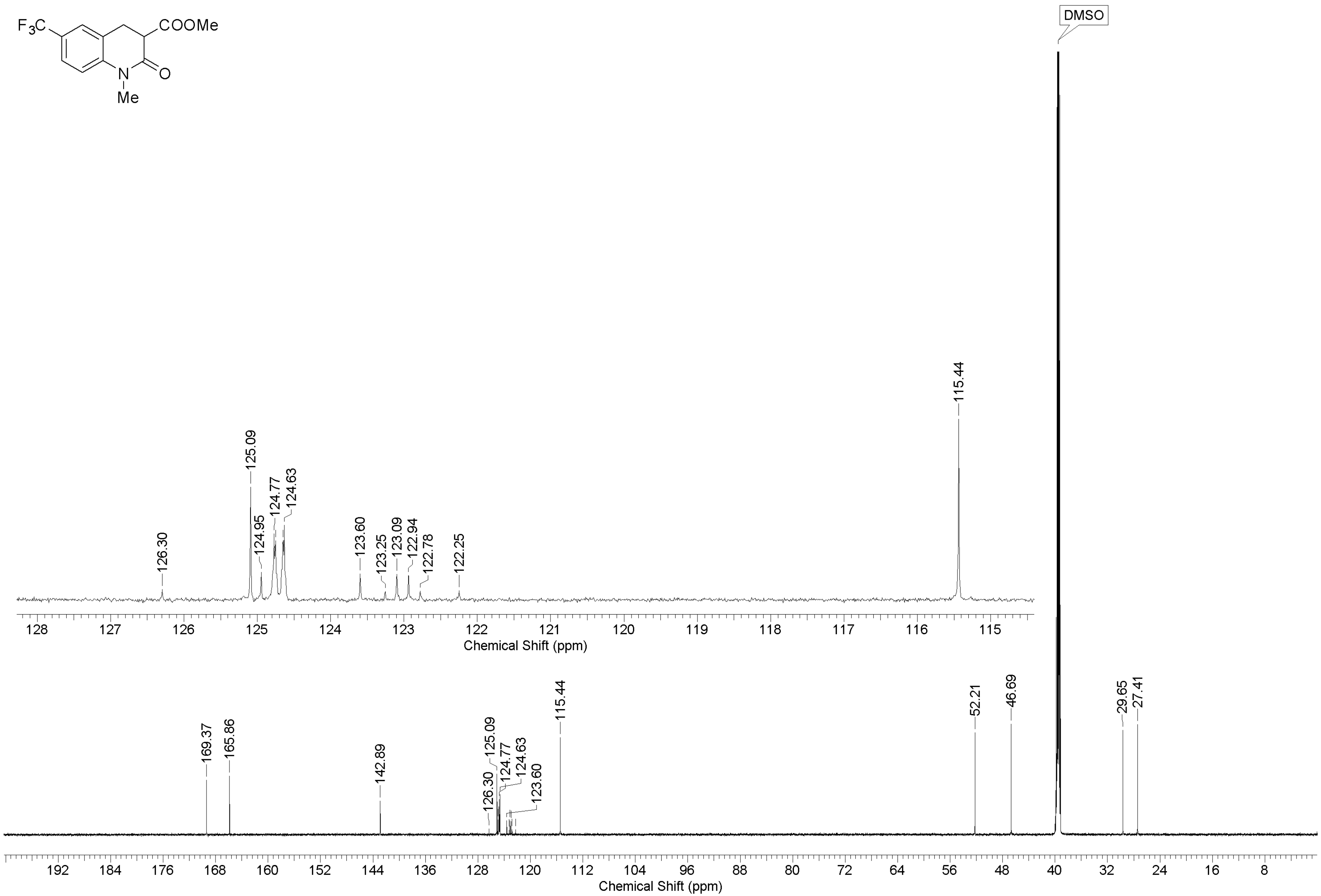
F3C
COOMe
N
O
Me
DMSO
115.44
125.09
124.95
124.77
124.63
126.30
123.60
123.25
123.09
122.94
122.78
122.25
128 127 126 125 124 123 122 121 120 119 118 117 116 115
Chemical Shift (ppm)
169.37
165.86
142.89
126.30
125.09
124.77
124.63
123.60
115.44
52.21
46.69
29.65
27.41
192 184 176 168 160 152 144 136 128 120 112 104 96 88 80 72 64 56 48 40 32 24 16 8
Chemical Shift (ppm)

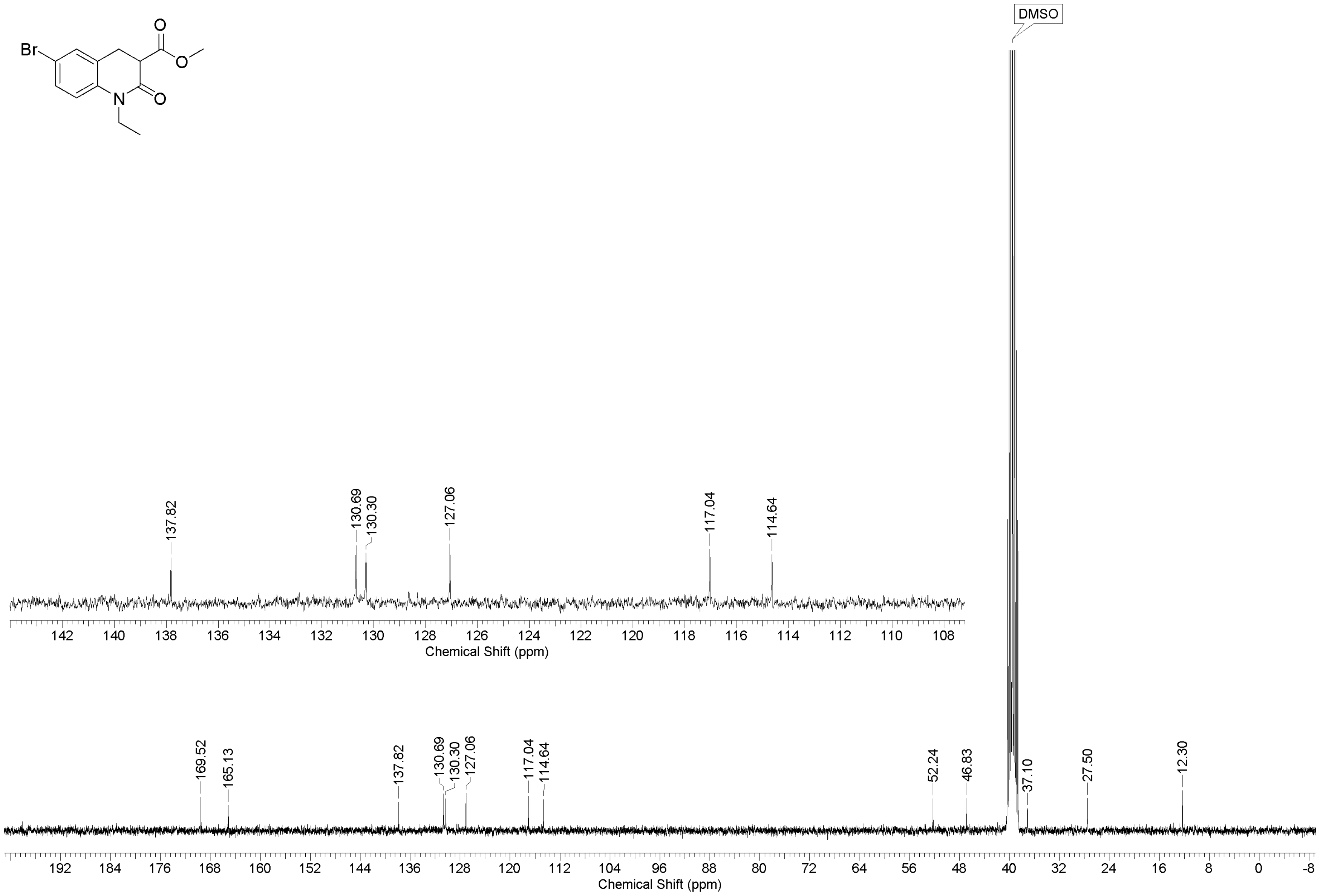
Br
O
O
N
O
DMSO
137.82
130.69
130.30
127.06
117.04
114.64
142 140 138 136 134 132 130 128 126 124 122 120 118 116 114 112 110 108
Chemical Shift (ppm)
169.52
165.13
137.82
130.69
130.30
127.06
117.04
114.64
52.24
46.83
37.10
27.50
12.30
192 184 176 168 160 152 144 136 128 120 112 104 96 88 80 72 64 56 48 40 32 24 16 8 0 -8
Chemical Shift (ppm)

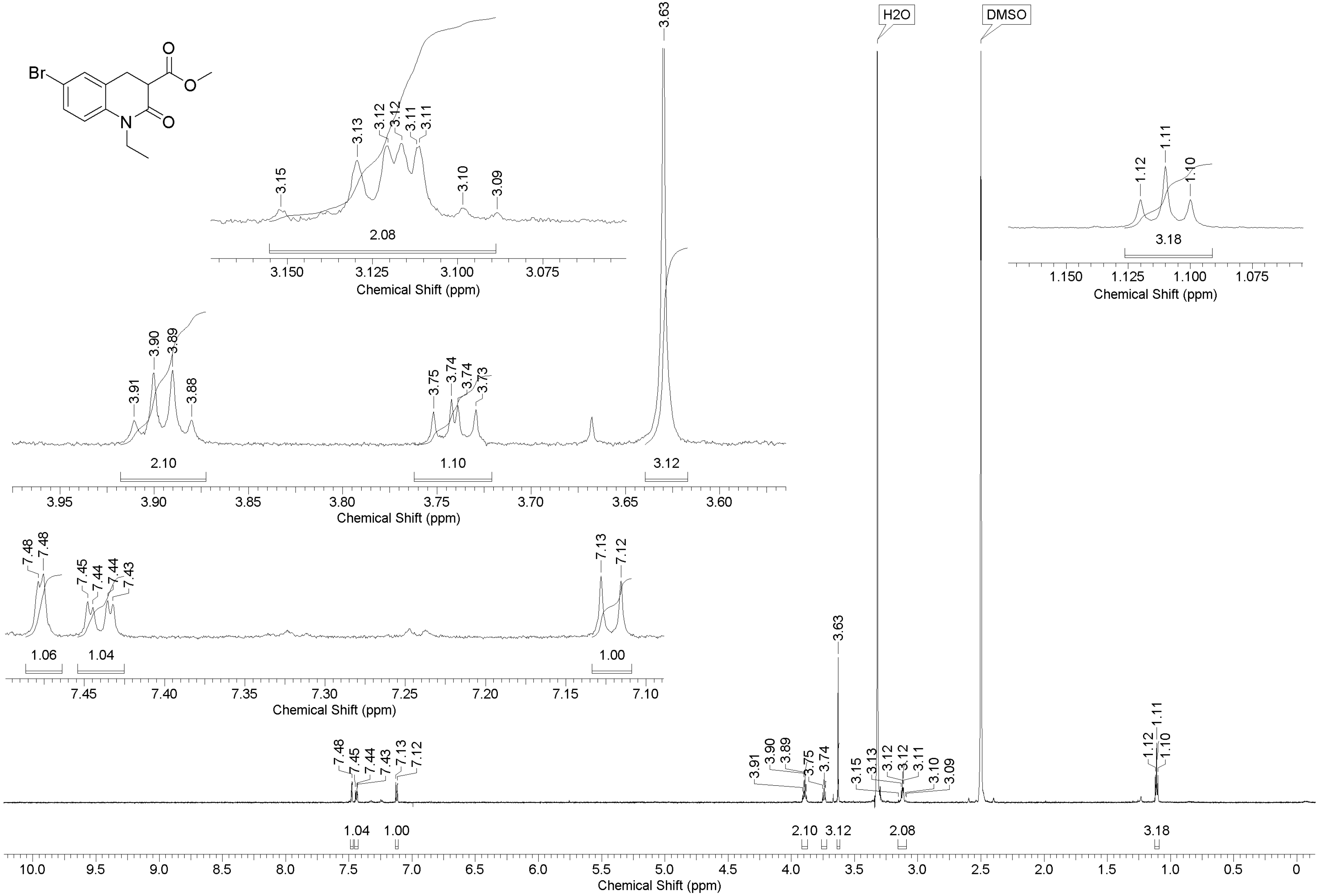
3.15
3.13
3.12
3.12
3.11
3.11
3.10
3.09
2.08
3.150
3.125
3.100
3.075
Chemical Shift (ppm)
3.63
H2O
DMSO
1.12
1.11
1.10
3.18
1.150
1.125
1.100
1.075
Chemical Shift (ppm)
3.91
3.90
3.89
3.88
3.75
3.74
3.74
3.73
2.10
1.10
3.12
3.95
3.90
3.85
3.80
3.75
3.70
3.65
3.60
Chemical Shift (ppm)
7.48
7.48
7.45
7.44
7.44
7.43
7.13
7.12
1.06
1.04
1.00
7.45
7.40
7.35
7.30
7.25
7.20
7.15
7.10
Chemical Shift (ppm)
1.04
1.00
2.10
3.12
2.08
3.18
10.0
9.5
9.0
8.5
8.0
7.5
7.0
6.5
6.0
5.5
5.0
4.5
4.0
3.5
3.0
2.5
2.0
1.5
1.0
0.5
0
Chemical Shift (ppm)

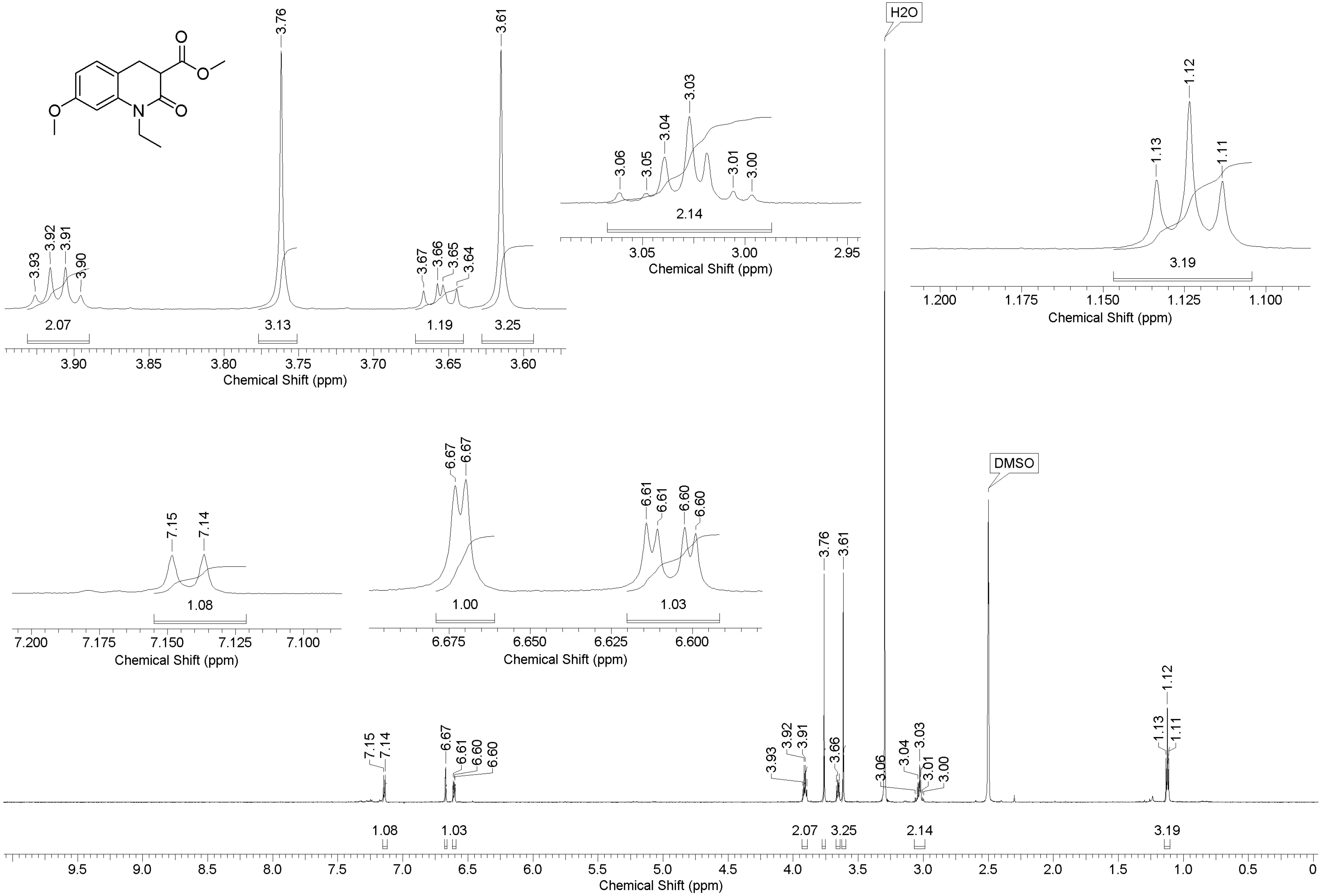

O
N
H2O
DMSO
2.07
3.13
1.19
3.25
2.14
3.19
1.08
1.00
1.03
Chemical Shift (ppm)

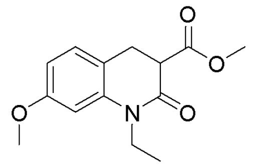

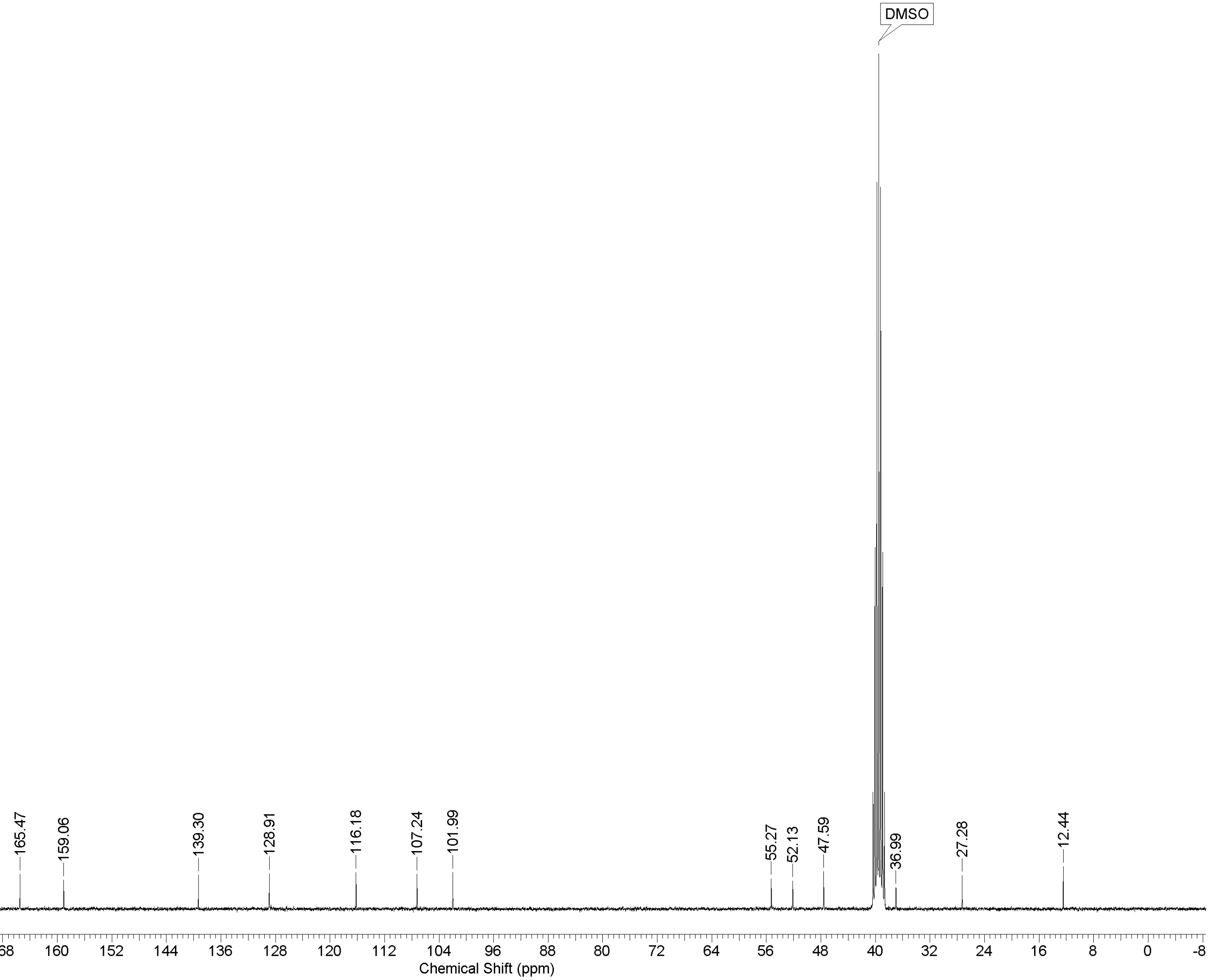
DMSO
169.78
165.47
159.06
139.30
128.91
116.18
107.24
101.99
55.27
52.13
47.59
36.99
27.28
12.44
200 192 184 176 168 160 152 144 136 128 120 112 104 96 88 80 72 64 56 48 40 32 24 16 8 0 -8
Chemical Shift (ppm)

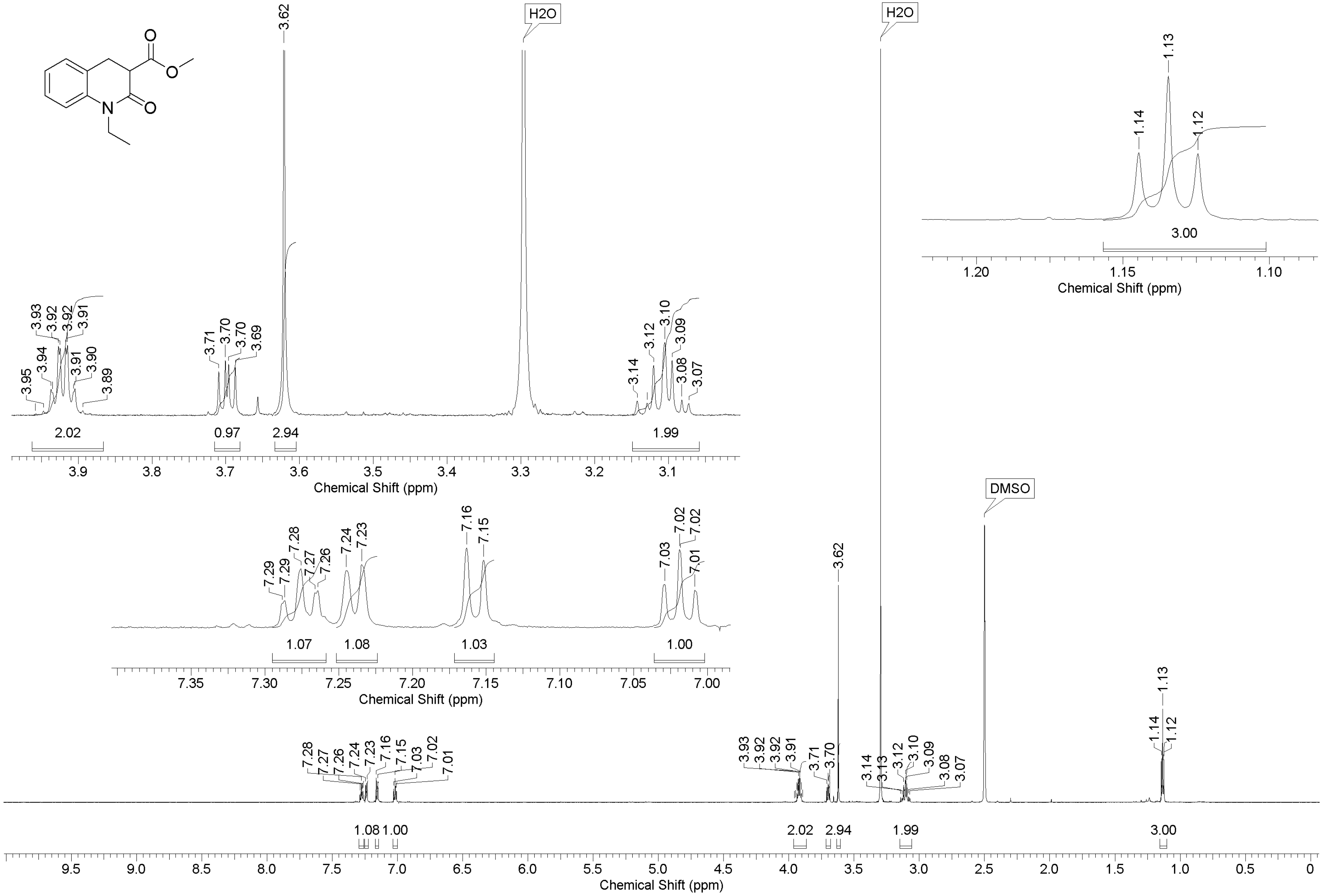
O
O
N
O
H2O
H2O
DMSO
3.62
1.13
1.14
1.12
3.00
1.20
1.15
1.10
Chemical Shift (ppm)
3.95
3.94
3.93
3.92
3.92
3.91
3.91
3.90
3.89
3.71
3.70
3.70
3.69
3.14
3.12
3.10
3.09
3.08
3.07
2.02
0.97
2.94
1.99
3.9
3.8
3.7
3.6
3.5
3.4
3.3
3.2
3.1
Chemical Shift (ppm)
7.29
7.29
7.28
7.27
7.26
7.24
7.23
7.16
7.15
7.03
7.02
7.02
7.01
1.07
1.08
1.03
1.00
7.35
7.30
7.25
7.20
7.15
7.10
7.05
7.00
Chemical Shift (ppm)
7.28
7.27
7.26
7.24
7.23
7.16
7.15
7.03
7.02
7.01
3.93
3.92
3.92
3.91
3.71
3.70
3.62
3.14
3.13
3.12
3.10
3.09
3.08
3.07
1.14
1.13
1.12
1.08
1.00
2.02
2.94
1.99
3.00
9.5
9.0
8.5
8.0
7.5
7.0
6.5
6.0
5.5
5.0
4.5
4.0
3.5
3.0
2.5
2.0
1.5
1.0
0.5
0
Chemical Shift (ppm)

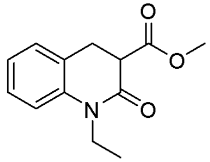

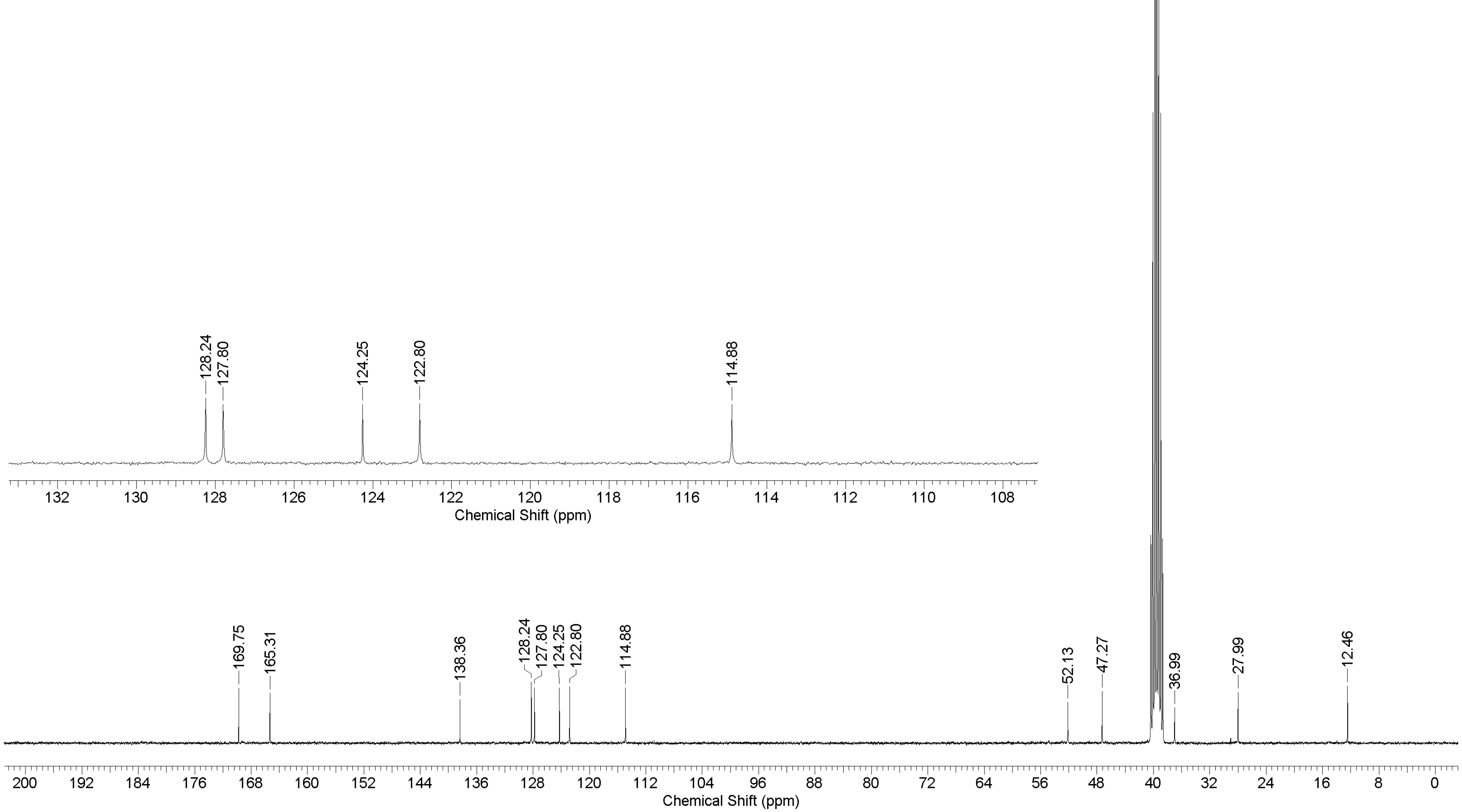
DMSO
128.24
127.80
124.25
122.80
114.88
132 130 128 126 124 122 120 118 116 114 112 110 108
Chemical Shift (ppm)
169.75
165.31
138.36
128.24
127.80
124.25
122.80
114.88
52.13
47.27
36.99
27.99
12.46
200 192 184 176 168 160 152 144 136 128 120 112 104 96 88 80 72 64 56 48 40 32 24 16 8 0
Chemical Shift (ppm)

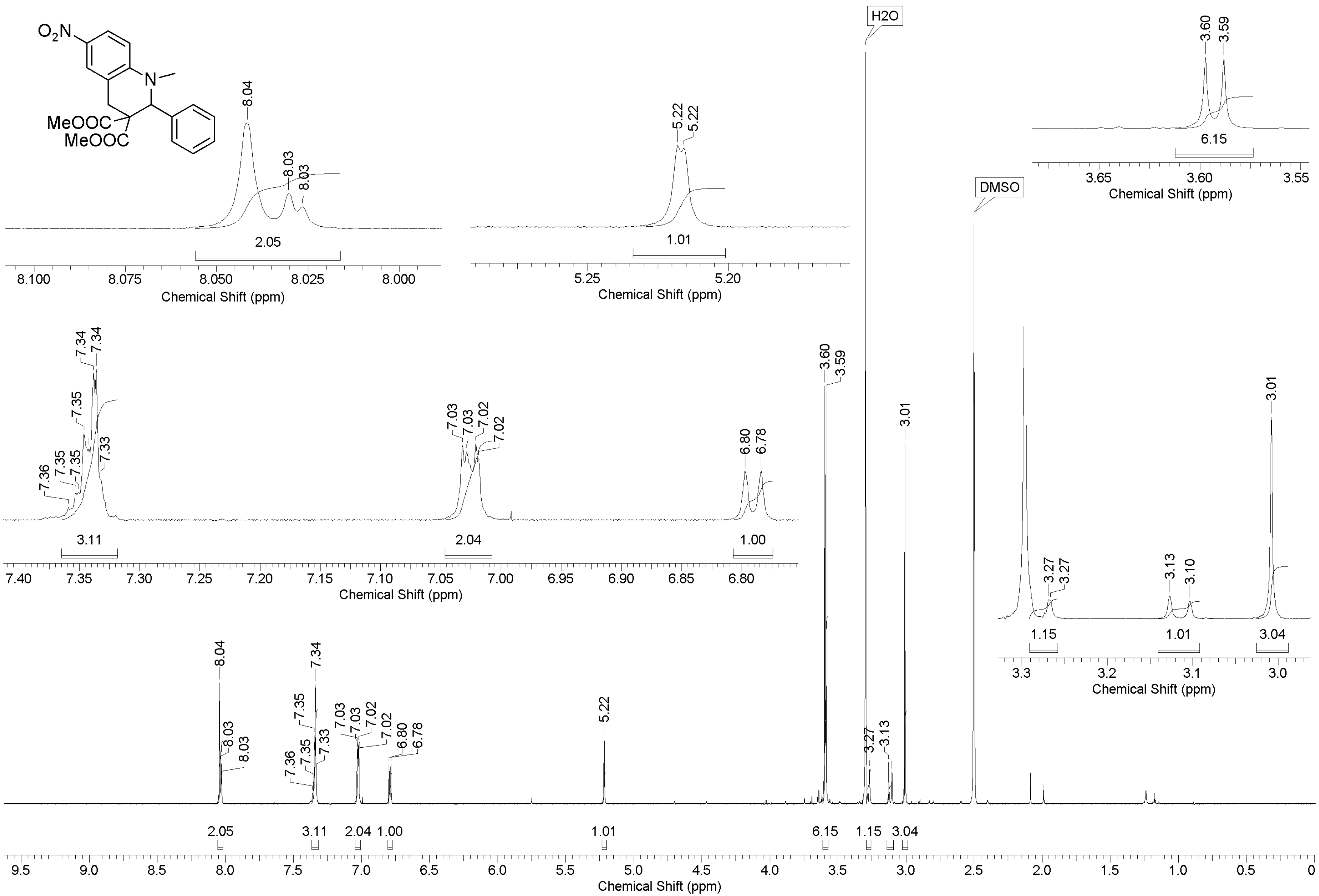

O_2N
N
MeOOC
MeOOC
H2O
DMSO
Chemical Shift (ppm)

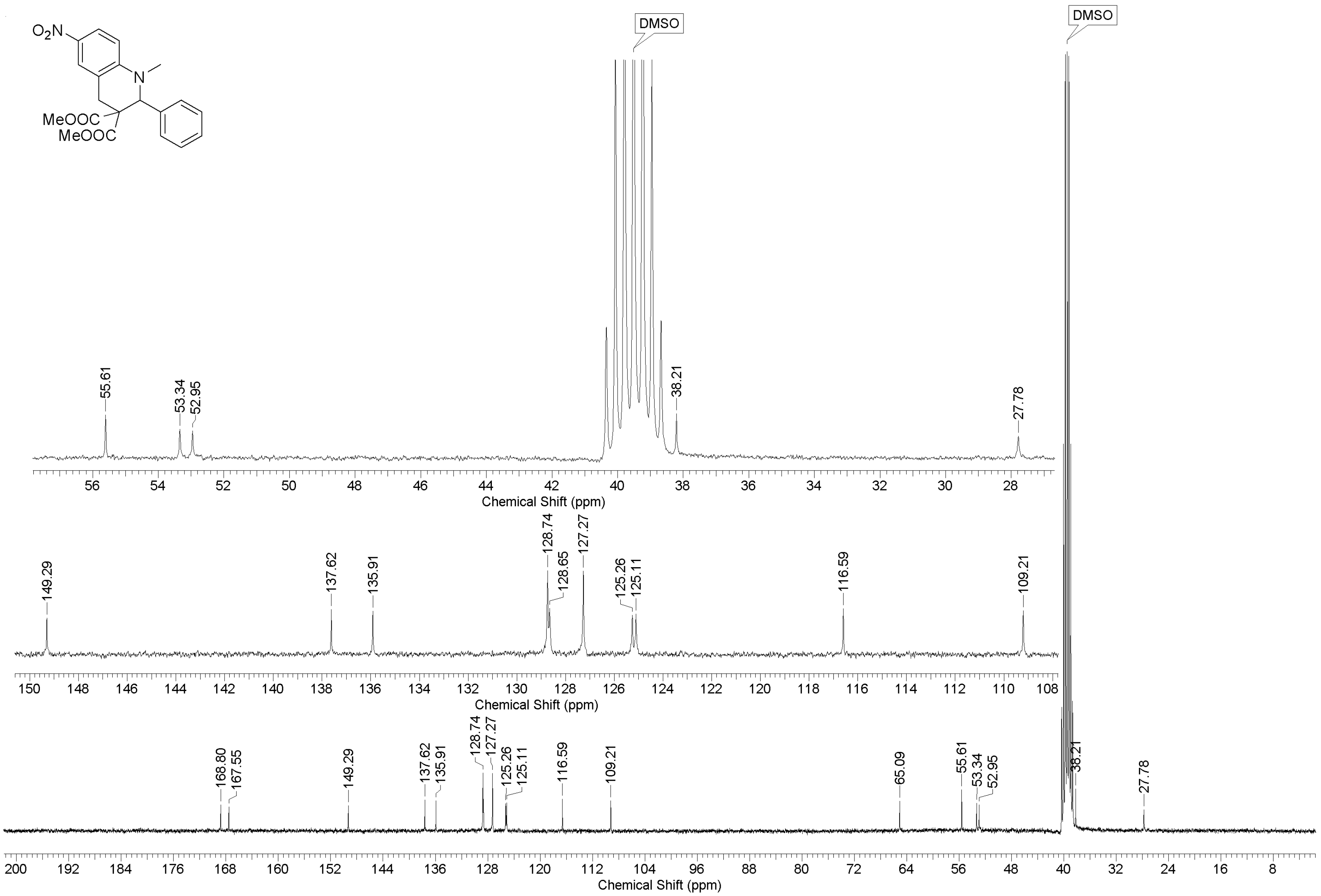

O_2N
N
MeOOC
MeOOC
DMSO
DMSO
55.61
53.34
52.95
38.21
27.78
Chemical Shift (ppm)
149.29
137.62
135.91
128.74
128.65
127.27
125.26
125.11
116.59
109.21
Chemical Shift (ppm)
168.80
167.55
149.29
137.62
135.91
128.74
127.27
125.26
125.11
116.59
109.21
65.09
55.61
53.34
52.95
38.21
27.78
Chemical Shift (ppm)

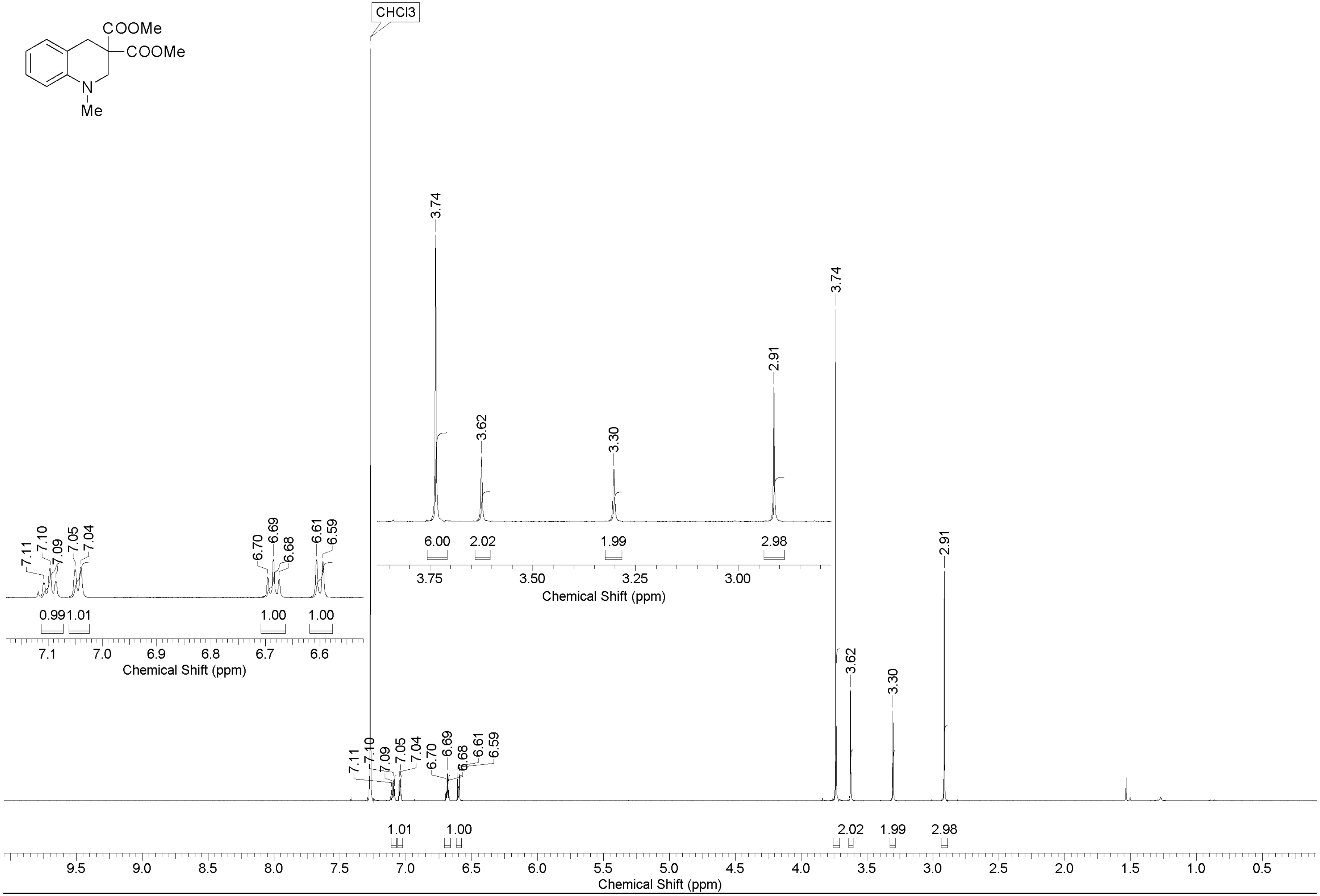

COOMe
COOMe
N
Me
CHCl3
3.74
3.62
3.30
2.91
6.00
2.02
1.99
2.98
3.75
3.50
3.25
3.00
Chemical Shift (ppm)
7.11
7.10
7.09
7.05
7.04
6.70
6.69
6.68
6.61
6.59
0.99
1.01
1.00
1.00
7.1
7.0
6.9
6.8
6.7
6.6
9.5
9.0
8.5
8.0
7.5
7.0
6.5
6.0
5.5
5.0
4.5
4.0
3.5
3.0
2.5
2.0
1.5
1.0
0.5

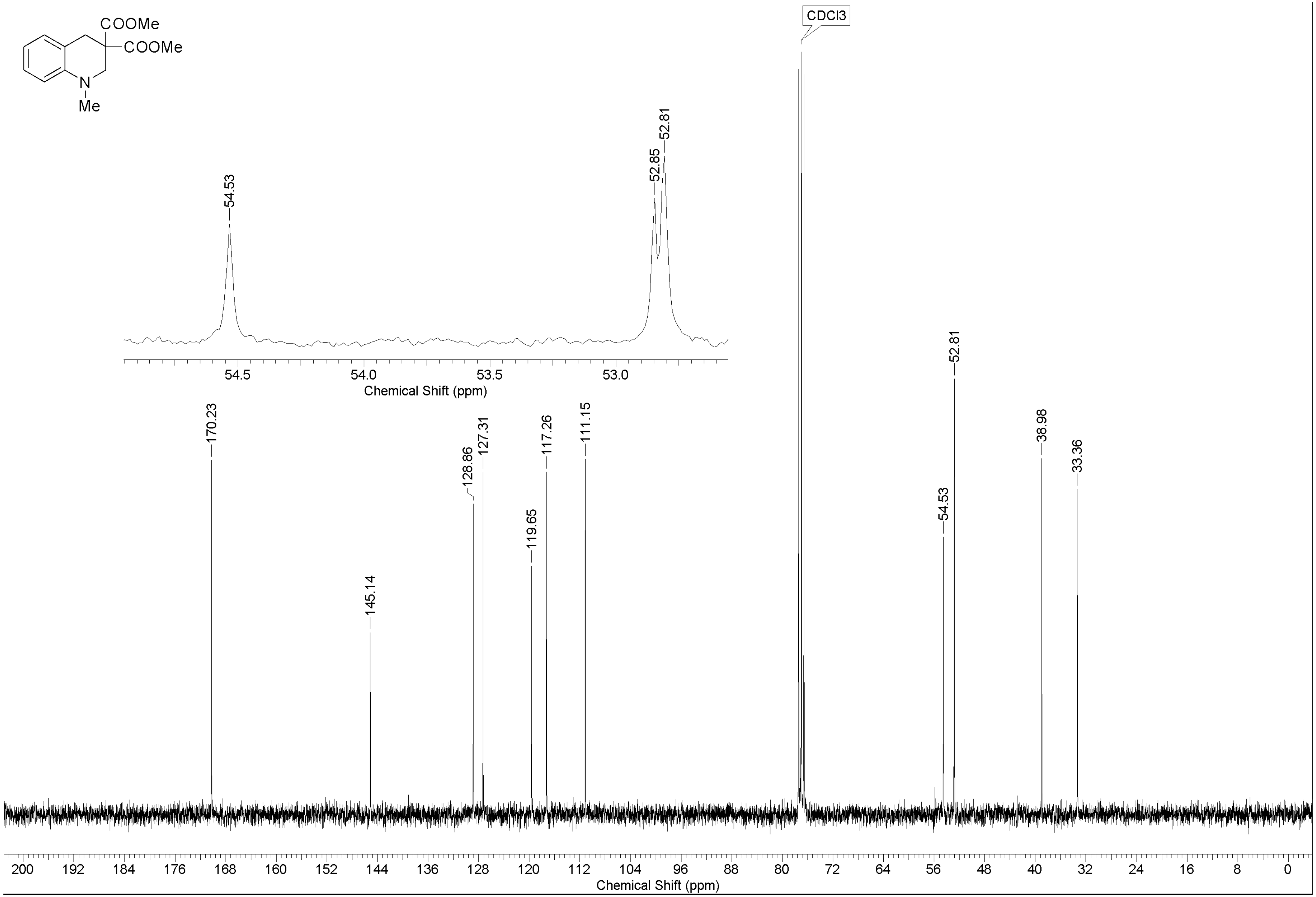
COOMe
COOMe
N
Me
CDCl3
54.53
52.85
52.81
54.5
54.0
53.5
53.0
Chemical Shift (ppm)
170.23
145.14
128.86
127.31
119.65
117.26
111.15
54.53
52.81
38.98
33.36
200 192 184 176 168 160 152 144 136 128 120 112 104 96 88 80 72 64 56 48 40 32 24 16 8 0
Chemical Shift (ppm)
